# Characterization of Constituents with Potential Anti-Inflammatory Activity in Chinese Lonicera Species by UHPLC-HRMS Based Metabolite Profiling

**DOI:** 10.3390/metabo12040288

**Published:** 2022-03-25

**Authors:** Eva-Maria Pferschy-Wenzig, Sabine Ortmann, Atanas G. Atanasov, Klara Hellauer, Jürgen Hartler, Olaf Kunert, Markus Gold-Binder, Angela Ladurner, Elke H. Heiß, Simone Latkolik, Yi-Min Zhao, Pia Raab, Marlene Monschein, Nina Trummer, Bola Samuel, Sara Crockett, Jian-Hua Miao, Gerhard G. Thallinger, Valery Bochkov, Verena M. Dirsch, Rudolf Bauer

**Affiliations:** 1 Institute of Pharmaceutical Sciences, University of Graz, 8010 Graz, Austria; eva-maria.wenzig@uni-graz.at (E.-M.P.-W.); ortmann.sabin@gmail.com (S.O.); klara.hellauer@uni-graz.at (K.H.); juergen.hartler@uni-graz.at (J.H.); olaf.kunert@uni-graz.at (O.K.); pia.g.raab@gmail.com (P.R.); marlene.monschein@icloud.com (M.M.); n.trummer1@gmail.com (N.T.); bola.samuel@hotmail.de (B.S.); sara.crockett@uni-graz.at (S.C.); valery.bochkov@uni-graz.at (V.B.); 2 Department of Pharmaceutical Sciences, University of Vienna, 1090 Vienna, Austria; atanas.atanasov@dhps.lbg.ac.at (A.G.A.); angela.ladurner@univie.ac.at (A.L.); elke.heiss@univie.ac.at (E.H.H.); slatkolik@gmail.com (S.L.); verena.dirsch@univie.ac.at (V.M.D.); 3 Ludwig Boltzmann Institute for Digital Health and Patient Safety, Medical University of Vienna, 1090 Vienna, Austria; 4 Institute of Genetics and Animal Biotechnology of the Polish Academy of Sciences, 05-552 Magdalenka, Poland; 5 Field of Excellence BioHealth, University of Graz, 8010 Graz, Austria; 6 Center for Physiology and Pharmacology, Medical University of Vienna, 1090 Vienna, Austria; markus.gold-binder@meduniwien.ac.at; 7 Guangxi Botanical Garden of Medicinal Plants, Nanning 530023, China; zh_ym2009@126.com (Y.-M.Z.); mjh1962@vip.163.com (J.-H.M.); 8 Institute of Biomedical Informatics, Graz University of Technology, 8010 Graz, Austria; gerhard.thallinger@tugraz.at; 9 OMICS Center Graz, BioTechMed-Graz, 8010 Graz, Austria

**Keywords:** *Lonicera*, honeysuckle, anti-inflammatory, metabolite profiling, UHPLC-HRMS, multivariate data analysis, OPLS-DA, traditional Chinese medicine

## Abstract

This study centered on detecting potentially anti-inflammatory active constituents in ethanolic extracts of Chinese Lonicera species by taking an UHPLC-HRMS-based metabolite profiling approach. Extracts from eight different Lonicera species were subjected to both UHPLC-HRMS analysis and to pharmacological testing in three different cellular inflammation-related assays. Compounds exhibiting high correlations in orthogonal projections to latent structures discriminant analysis (OPLS-DA) of pharmacological and MS data served as potentially activity-related candidates. Of these candidates, 65 were tentatively or unambiguously annotated. 7-Hydroxy-5,3′,4′,5′-tetramethoxyflavone and three bioflavonoids, as well as three C_32_- and one C_34_-acetylated polyhydroxy fatty acid, were isolated from *Lonicera hypoglauca* leaves for the first time, and their structures were fully or partially elucidated. Of the potentially active candidate compounds, 15 were subsequently subjected to pharmacological testing. Their activities could be experimentally verified in part, emphasizing the relevance of Lonicera species as a source of anti-inflammatory active constituents. However, some compounds also impaired the cell viability. Overall, the approach was found useful to narrow down the number of potentially bioactive constituents in the complex extracts investigated. In the future, the application of more refined concepts, such as extract prefractionation combined with bio-chemometrics, may help to further enhance the reliability of candidate selection.

## 1. Introduction

Lonicera species are an indispensable part of the therapeutic armamentarium of traditional Chinese medicine (TCM). Lonicera species listed in the Chinese Pharmacopoeia include *Lonicera japonica* Thunb., known as the source of Lonicerae japonicae flos (Jinyinhua) and Lonicerae caulis (Rendongteng), as well as *L. hypoglauca* Miq., *L. fulvotomentosa* Hsu et S.C. Cheng (accepted name: *L. macrantha* (D. Don) Spreng) and *L. macranthoides* Hand. Mazz. (accepted name: *L. macrantha* (D. Don) Spreng). The latter three species are the sources of the drug Lonicerae flos (Shanyinhua) [1,2].

Extensive research has been performed on *Lonicera japonica* [3,4]. *L. japonica* extracts and constituents have shown antibacterial, antiviral, anti-inflammatory, immunoregulatory, antitumor and hepatoprotective effects in vitro and in animal experiments [4,5]. The plant has also entered the spotlight in recent research due to its potential activity against SARS-CoV-2 infection. It is among the most commonly used herbs in TCM formulae recommended by Chinese health authorities in COVID-19 prevention programs [6,7]. Extensive phytochemical investigations of the plant have been performed, and more than 200 secondary metabolites have been identified from *L. japonica* flowers and aerial parts [3,4]. The plant parts mainly used for medicinal purposes are the flowers, followed by the stems [3,4]. The leaves have not commonly been used for medical purposes in the past but have recently attracted an increasing amount of attention, since their chemical profile has been found to be similar to that of *L. japonica* flowers [8]. The other species listed in the Chinese Pharmacopoeia and many other Lonicera species that occur as native or endemic representatives in China have been much less thoroughly investigated than *L. japonica*. 

Classically, the discovery of bioactive natural products from plant sources involves activity-guided fractionation of active extracts. This is a time- and labor-intensive process with major shortcomings. Frequently, the bioactive constituents cannot be identified due to irreversible binding on chromatographic material or due to degradation during the separation process. The isolation of constituents that occur in low amounts also presents a challenge, since material amounts decrease with each fractionation step. Moreover, in many cases, already-known active constituents are isolated [9,10,11]. Through the development and broader application of advanced analytical instruments, metabolomics-based approaches have emerged as alternative tools for identifying active constituents present in complex herbal extracts [11,12,13]. One frequently used strategy is the metabolite profiling of bioactive natural extracts by means of ultra-high-performance liquid chromatography coupled to high-resolution mass spectrometry (UHPLC-HRMS). Since structural information can be obtained by taking this approach, the active constituents can be dereplicated (i.e., annotated) prior to isolation. In addition, linking metabolite profiling data with bioactivity data can enable the researcher to prioritize potentially bioactive natural products [12]. Chemometric analytical tools are currently being used to identify putative active constituents by correlating phytochemical and bioactivity data. These tools include partial least squares projections to latent structures (PLS), orthogonal PLS (OPLS) and orthogonal projections to latent structures-discriminant analysis (OPLS-DA) [9,14].

Our group previously applied attenuated total reflectance Fourier-transform infrared spectroscopy (ATR-FTIR) successfully in order to classify Lonicera samples displaying different degrees of in vitro anti-inflammatory activity [15]. However, ATR-FTIR cannot be used to trace back the active compounds in extracts of known activity. Thus, the aim of this study was to use UHPLC-HRMS and chemometrics to identify constituents in these extracts that could be associated with in vitro anti-inflammatory activity. We correlated UHPLC-HRMS phytochemical profiles with bioactivity data from the leaf extracts of eight different Lonicera species native to China by means of OPLS-DA. The candidate compounds derived from the OPLS-DA models were annotated on the basis of HRMS data. By taking this approach, we could annotate 65 potentially bioactive compounds and subsequently isolate eight of these candidate compounds. The in vitro anti-inflammatory properties of 15 candidate compounds present in different active Chinese Lonicera species were evaluated.

## 2. Results

### 2.1. Lonicera Extracts Displayed High Chemical Diversity

The assignment of the plant samples to eight different Lonicera species by morphological analysis and DNA barcoding has been previously described [15] (see Supplementary Table S1). The ethanolic leaf extracts were analyzed by UHPLC-HRMS. HESI-negative ion mode detection was chosen based on the results of pilot experiments, which indicated that the best metabolite coverage could be obtained under these conditions (data not shown). UHPLC-HRMS analysis results revealed that the extracts are complex mixtures containing constituents with a wide polarity range and that their composition was highly diverse (Supplementary Figure S1).

**Figure 1:**
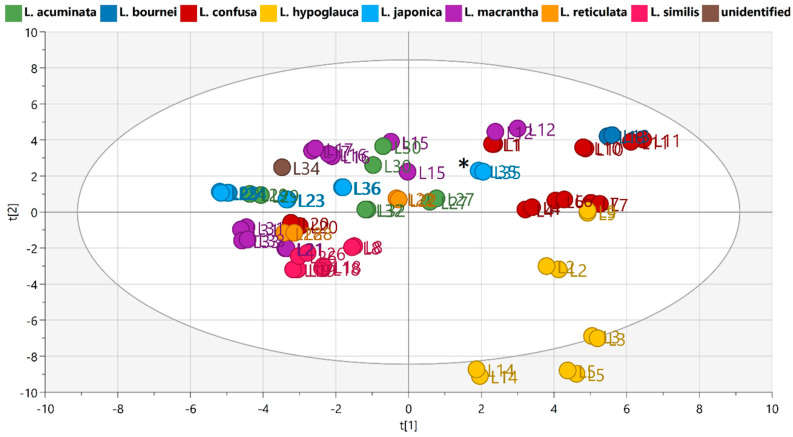
PCA score scatter plot [t1]/[t2] of preprocessed, normalized UHPLC-HRMS HESI-negative ion mode data (14 principal components: R2X (cum) = 0.796, Q2 (cum)= 0.519; R2X [t1]= 0.147, R2X [t2]= 0.126; Model window, see Supplementary Table S3). Samples are colored according to species: green = *L. acuminata*; dark blue = *L. bournei*; red = *L. confusa*; yellow = *L. hypoglauca*; light blue = *L. japonica* [*: samples L35_1 and L35_2, *L. japonica* flower bud extracts]; violet = *L. macrantha*; orange = *L. reticulata*; pink = *L. similis*; dark brown = unidentified.

In order to obtain an overview of the chemical diversity of the studied extracts, the preprocessed normalized UHPLC-HRMS data were subjected to the principal component analysis (PCA). In the PCA score scatter plot (Figure 1), 14 principal components altogether explained 79.6% of the observed variation. Replicate extracts consistently clustered together, indicating the high reproducibility of the extraction method and UHPLC-HRMS analysis. All extracts derived from *L. similis* accessions formed a dense cluster, indicating that they had similar chemical compositions, while extracts from *L. hypoglauca*, *L. acuminata*, *L. confusa* and *L. japonica* formed broad clusters, indicating that these species had highly variable phytochemical profiles. *Lonicera macrantha* extracts tended to form two distinct clusters. The variation between these two clusters could clearly be seen in the UHPLC-HRMS chromatograms (see Supplementary Figure S2). In the case of *L. japonica*, the flower bud extract (L35) showed a clear separation from the *L. japonica* leaf extracts (L23-25 and L35) in the PCA score scatter plot [t1]/[t2] (Figure 1), indicating phytochemical differences between leaves and flower buds; such differences were also revealed by the UHPLC-HRMS chromatograms (Supplementary Figure S3).

Overall, the results of PCA indicated the broad phytochemical variability between and within the Lonicera species under investigation.

### 2.2. Lonicera Extracts Displayed Distinct In Vitro Anti-Inflammatory Activities

**Figure 2:**
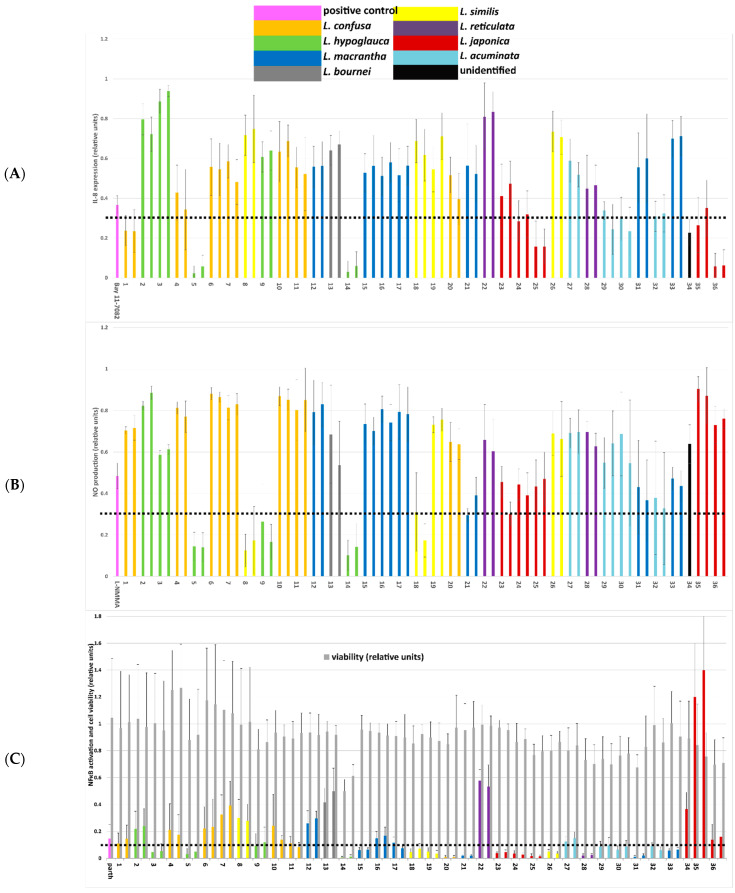
Results of activity testing of Lonicera ethanolic leaf extracts 1–36 (colored according to species) on the production of inflammation mediators. (**A**) Effect on IL-8 expression in HUVECtert cells stimulated with LPS. Extract concentration: 50 µg/mL; positive control: Bay 11-7082 (7.5 µM); results are given as the means ± SD of 6 replicates. (**B**) Effect on NO production in LPS/IFNγ-stimulated RAW macrophages. Extract concentration: 50 µg/mL; Positive control: L-NMMA (100 µM); results are given as the means ± SD of three experiments performed in duplicates. (**C**) Effect on NF-κB transactivation and on cell viability in HEK/NFκB-luc cells stimulated with TNF-α; extract concentration: 50 µg/mL; positive control: parthenolide (5 µM). Results are given as the means ± SD of 4 replicates (each in quadruplicate).

To evaluate the pharmacological activity of the extracts, all extracts were assayed in three different cellular in vitro models that are relevant in the context of inflammation. The effects on the expression of IL-8 were screened in LPS-stimulated endothelial HUVECtert cells (Figure 2A); the influence on NO release was investigated in LPS/IFN-γ-stimulated RAW 264.7 mouse macrophages (Figure 2B), and modulation of the activity of the nuclear transcription factor NF-κB (Figure 2C) was examined in HEK293 cells transfected with a luciferase reporter gene. In these cells, the potential impact of the extracts on cell viability was assessed (Figure 2C).

The extracts showed some parallels regarding their influence on the release of NO and IL-8. The most pronounced activity was found for *L. hypoglauca* accessions L5 and L14, whereas *L. macrantha*, *L. reticulata*, *L. acuminata* and *L. bournei* samples showed no or only moderate activity in both assays. Furthermore, the activity of *L. confusa* and *L. similis* accessions was generally low with the exception of L1 and L18, which showed pronounced effects on the IL-8 levels and NO production, respectively. *Lonicera japonica* extracts showed generally higher activity in the IL-8 assay. Many of the extracts showed highly pronounced inhibitory activity on NF-κB activation. Cell viability was not or only moderately affected at screening concentrations of 50 µg/mL; only in the case of L14, a reduction to about 60% of the viability of control samples was observed.

Black dashed lines indicate the threshold below which extracts were considered as highly active in the respective assay.

### 2.3. Candidate Compounds from Different Compound Classes Were Annotated from OPLS-DA Models Correlating UHPLC-HRMS and Activity Data

In order to trace back the constituents that most likely correlate with anti-inflammatory activities, activity data were correlated with processed UHPLC-HRMS data by means of OPLS-DA, a supervised multivariate data analysis (MVDA) method. In OPLS-DA, a regression model is calculated between the multivariate data (i.e., the UHPLC-HRMS dataset) and a response variable (i.e., the class information). The class information is placed in the first predictive component, while the other components describe the variation orthogonal to the predictive component [16]. 

In the score scatter plots of all tree OPLS-DA models, a clear discrimination between active and inactive samples was observed (Supplementary Figures S4–S6), and the quality of the models can be considered as good (Table 1). 

From the OPLS-DA models, S-plots were generated to visualize the covariance and correlation between the scores for the predicted OPLS-DA component and the spectral variables, as well as to derive variables most likely correlated with class separation. High covariance and high correlation to the score on the predictive component indicate the most important variables [17]. Thus, the variables related to active extracts were sorted in a descending order according to covariance, and the 30 most reliable variables plus the interjacent, less reliable ones in the S-plots of the three models were selected as candidates. Since some of these were correlated with more than one activity, this resulted in the identification of 78 candidate features. The S-plots are shown in Supplementary Figures S4–S6 (candidate features marked in red). The complete candidate list is provided in Supplementary Table S4.

Four of the features were found to be dimers or adducts of other candidate compounds, and nine features could not be annotated. Of the remaining 65 features, seven could be unambiguously assigned, and 58 were initially tentatively annotated on the basis of their HRMS data; subsequently, eight of these tentatively annotated compounds were isolated from a *L. hypoglauca* ethanolic extract, and their structures were partially or fully elucidated by NMR spectroscopy (described in detail in Section 2.4). The mass spectrometric data of the annotated candidates are found in Table 2 and in the Supplementary Table S4.

The main compound classes annotated among the candidate compounds ranged from rather polar compounds, such as hydroxycinnamic acid derivatives, flavonoid glycosides and iridoids, to medium-polar compounds, such as flavonoid aglycones, bioflavonoids and fatty acids, and on to rather nonpolar lipids.

Concerning the hydroxycinnamic acid derivatives, dicaffeoylquinic acid isomers (**11** and **12**), coumaroylquinic acid isomers (**2** and **3**), dicoumaroylquinic acid isomers (**15** and **17**) and caffeic acid (**1**) could be annotated. Caffeoylquinic acid derivatives are widespread in the flower buds, stems and leaves of various Lonicera species [21,22,23], and also, several *p*-coumaroylquinic acid isomers have been detected [4]. This is the first time that di-*O*-*p*-coumaroylquinic acid derivatives are reported from this genus.

From the compound class of flavonoids, ten flavonoid-*O*-glycosides were annotated (**5**, **6**, **7**, **8**, **9**, **10**, **13**, **14**, **16** and **20**). Four of these were monoglycosides (**7**, **9**, **13** and **14**); four were diglycosides (**5**, **6**, **8** and **10**) and two were substituted with one sugar and one a caffeoyl (**16**) or coumaroyl (**20**) moiety. Compound **7** was unambiguously identified as luteolin-7-glucoside. In the MS/MS spectra of **5**, **6**, **8**, **10**, **13**, **16** and **20**, *m*/*z* 285.04 was detected as aglycone. However, since no aglycone breakdown fragments were detectable, it was impossible to determine whether the aglycone was luteolin or kaempferol on the basis of the MS/MS spectra generated with QExactive MS. Therefore, extracts containing the respective compounds were additionally analyzed on an LTQ-XL instrument equipped with a linear ion trap analyzer that provides MS^n^ capabilities. Comparison of MS^3^ spectra with reference spectra of luteolin and kaempferol allowed the annotation of the aglycones. The flavonoid monoglycoside **13** was assigned to a kaempferol-3-hexoside. Kaempferol was also detected as the aglycone moiety of diglycoside **8**, for which the MS/MS spectrum displayed two monoglycoside fragments, i.e., *m*/*z* 447.093 [[M-H]^−^ desoxyhexose]^−^ and 431.098 [[M-H]^−^ hexose]^−^, indicating that the sugar moieties were located on two different positions of the aglycone. Comparison with the literature led to a tentative annotation of this compound as kaempferol-3-hexoside-7-desoxyhexoside. Compounds **5**, **6** and **10** were assigned as the dihexoside, hexoside-pentoside and hexoside-desoxyhexoside of luteolin, respectively; the luteolin hexoside desoxyhexoside lonicerin (luteolin-7-neohersperidoside) has been previously identified as possible marker for *L. japonica* [24]. For compounds **16** and **20**, MS/MS fragmentation patterns clearly indicated the presence of caffeoyl (**16**) and coumaroyl (**20**) moieties. The aglycone was annotated as luteolin in both cases. Thus, compound **16** was tentatively identified as a luteolin caffeoyl hexoside, and compound **20**, as luteolin coumaroyl hexoside. This is the first reported detection of these two compounds in Lonicera species. 

Compounds **9** and **14** were annotated as isorhamnetin hexoside isomers. Due to the high abundance of the radical aglycone ion *m*/*z* 314.043 that is formed by homolytic cleavage, compound **14** was annotated as isorhamnetin-3-*O*-hexoside and compound **9** with the predominant aglycone ion *m*/*z* 315.051 as isorhamnetin-7-*O*-hexoside [25,26]. Isorhamnetin-3-*O*-glucoside has been described in Lonicera species previously [27].

In addition to the flavonoid glycosides, the flavone aglycones luteolin (**18**) and apigenin (**21**) were unambiguously identified. Compound **19** was annotated as the flavonol aglycone isorhamnetin. Compound **23** was tentatively identified as methylated flavone, but due to the high level of spectral similarity, it was impossible to discriminate between diosmetin or chrysoeriol on the basis of the MS/MS fragmentation pattern. The MS/MS spectrum of compound **22** indicated the presence of a polymethoxyflavone. This compound was subsequently isolated, and its structure identified by NMR spectroscopy (see Section 2.4).

Furthermore, a series of candidates were annotated as biflavonoids: amentoflavone (**32**) and podocarpusflavone A (**33**) were identified by comparison with authentic reference compounds. The fragmentation patterns of compounds **34** and **35** were highly similar and clearly indicated C-*O*-linked biapigenins, such as the 4′,6″-*O*-biapigenin hinokiflavone [28]. A similar fragmentation can be expected for the 4′,3‴-*O*-biapigenin ochnaflavone, which has previously been isolated from *L. japonica* [29]. Compounds **26**, **27** and **29** were tentatively identified as biapigenin, mono- and dimethylbiapigenin and were subsequently isolated and structurally identified by NMR spectroscopy (see Section 2.4).

From the group of terpenoids, one iridoid glycoside and three triterpene glycosides appeared as candidates. The iridoid glycoside secoxyloganin was unambiguously identified (**4**), which has already been described as a constituent of Lonicera species [8,30]. The triterpene glycosides were tentatively annotated as the hederagenin glycosides Akebia saponin D (**30**), Akebia saponin C (**37**) and Akebia saponin PA (**38**), all of which have been previously isolated from several Lonicera species [8,31,32].

Concerning nitrogen-containing compounds, we were able to annotate the alkaloid aurantiamide acetate (**31**) and to unambiguously identify the chlorophyll degradation product phaeophorbide A (**52**). Both compounds have not been identified in Lonicera species until now.

Finally, numerous OPLS-DA candidates were annotated as fatty acids and assigned to different types of lipids. Two trihydroxy octadecadienoic acid isomers (**24** and **25**) and an oxo-octadecadienoic acid isomer (**36**) were annotated, next to a C_24_ monohydroxy fatty acid (**58**). Furthermore, a series of 12 thus far undescribed long-chain fatty acid derivatives were annotated (**41**–**44**, **46**, **47**, **49**–**51**, **53**, **54** and **56**). Four of these (**43**, **44**, **46** and **54**) were subsequently isolated, and their structures were partially characterized (see detailed description in Section 2.4). Molecular formulas and MS/MS fragmentation patterns indicated that the 12 compounds possessed chain lengths of C_32_ (**41–44** and **46**) or C_34_, two to four hydroxyl groups, as well as one to three acetyl moieties. To our knowledge, these compounds have not previously been described in literature.

With respect to lipids, candidates with phospholipid, glycolipid, cerebroside and ceramide structures were tentatively identified. Compound **39** was annotated as a glycolipid with an octadecatrienoic acid and a dihexoside moiety bound to glycerol, like gingerglycolipid A. Compound **48** was tentatively identified as glycerol bearing a palmitic acid moiety as well as a sulfur-containing sugar moiety, such as palmitoyl-sulfoquinovosylglycerol. Compounds **40**, **45** and **55** were annotated as phospholipids, namely as lysophosphatidylcholine (LPC) bearing a C_18_ fatty acid with three double bonds (**40**), a lysophosphatidylglycerol (LPG) bearing a C_16_-monounsaturated fatty acid (**45**) and an LPG with a saturated C_16_ fatty acid (**55**). The structure of the LPC was additionally verified by the software Lipid Data Analyzer [33] (see Supplementary Figure S7). None of these glycolipids or phospholipids have been previously reported in Lonicera species.

Finally, seven candidate compounds were assigned to ceramides that contained either a hydroxysphingosine (**59**–**63** and **65**) or hydroxysphinganine isomer (**64**) as a long-chain base and saturated C_22_-, C_23_- or C_24_-mono- or dihydroxy fatty acids [34]. Their structures could be additionally verified by Lipid Data Analyzer [35] (see Supplementary Figures S7–S14). Finally, **57** was annotated as a cerebroside composed of a sphingadienine isomer, a saturated C_16_-dihydroxy fatty acid and a hexose moiety, such as soyacerebroside isomers. Soyacerebroside II, as well as several ceramides have been previously isolated from *L. japonica* [36,37]. The ceramide lonijaposide A3 has the same molecular formula as compound **61** (C_42_H_83_O_6_N. However, the MS/MS fragmentation pattern of compound **61** is more similar to that of a ceramide composed of a hydroxysphingosine isomer and a dihydroxytetracosanoic acid [34] than the structure of lonijaposide A3, consisting of a C_26_ long-chain base and an α-hydroxy hexadecanoic acid [37].

### 2.4. Eight OPLS-DA Candidates Were Isolated from *L. hypoglauca* Leaves

**Figure 3:**
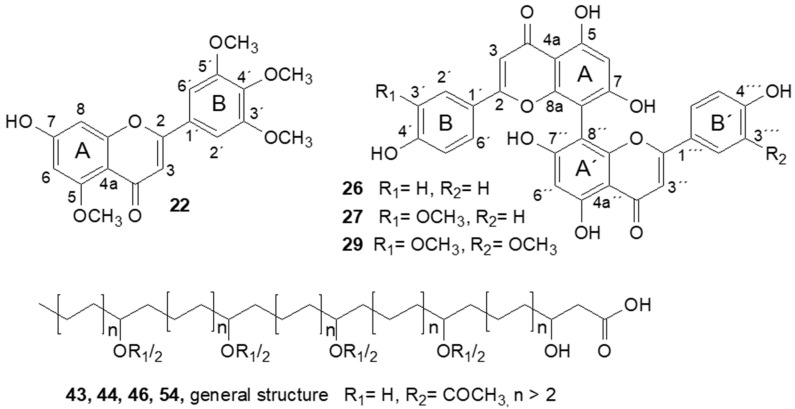
Structures of isolated compounds **22**, **26**, **27** and **29** and general structure of isolated compounds **43**, **44**, **46** and **54**. HMBC correlations; see Supplementary Figure S15.

To identify the structures of some of the candidate compounds that could not be unambiguously identified by UHPLC-HRMS analysis, a large-scale extract was prepared from *L. hypoglauca* leaves, since samples of this species had been found to contain several candidates at high levels (i.e., normalized peak area > average + 2 S.D. in OPLS-DA Xvar plot, Supplementary Table S4). From this extract, eight compounds could be isolated. Compound **22** was obtained as yellow amorphous powder. From HRMS data, the molecular formula C_19_H_18_O_7_ could be deduced, and MS/MS fragments indicated the presence of methyl groups in the molecule. ^1^H and HSQC data indicated the presence of a pentahydroxyflavone with four methyl groups. The number of methin and methyl resonances detected by NMR spectroscopy (Table 3) suggested that the B ring is substituted at four positions symmetrically. HMBC correlations (see Supplementary Figure S15) allowed us to confirm that ring B is substituted with methoxy groups at positions 3′, 4′ and 5′. A further methoxy group was assigned to position 5. On the one hand, no proton resonance was detected for position 5 and, on the other hand, carbon shifts were significantly reduced at positions C-2 and C-4 but significantly enhanced at position C-3. Comparing the ^13^C shifts at these positions with the literature data on flavones with similar substitution pattern [38] allowed us to confirm the substitution of position 5 as a methoxy group. Consequently, **22** was identified as 7-hydroxy-5,3′,4′,5′-tetramethoxyflavone (Figure 3). Only one single patent describes the synthesis of this compound, but without disclosing spectroscopic data [39]. Accordingly, this is the first study reporting the isolation of **22** from a natural source and describing its spectroscopic assignment. Flavones with more than two methoxy groups in the molecule have not previously been described from *L. hypoglauca*; however, various highly methoxylated flavones have been identified in *L. japonica* flower buds [40].

Compounds **26**, **27** and **29** were obtained as yellow amorphous powders with an 8,8″-biapigenin skeleton. HRMS data indicated the presence of three related compounds which differed by the numbers of OCH_3_ units. MS/MS fragmentation patterns indicated that compounds **26** and **29** were symmetrically substituted. Only one retro-Diels–Alder fragment was observed in their MS/MS spectra (*m*/*z* 375.0496 for **26** and *m*/*z* 405.0601 for **29**); in contrast, two retro-Diels–Alder fragments were observed for the asymmetrically substituted compound **27**. The ^1^H NMR data of **26** also indicated the presence of a symmetrically substituted biapigenin. The position of the linkage between the two apigenin moieties was determined by comparing the proton and carbon shifts at positions 6 and 8 with those of apigenin (Supplementary Table S5). Thus, we could assign the A-ring-CH group with δH 7.04 ppm and δC 100.7 ppm to position C-6, and **26** was identified as the known 8,8″-biapigenin cupressuflavone (Figure 3). Cupressuflavone has been identified in several Lonicera species [41,42], but this represents a new report for *L. hypoglauca*.

The ^1^H spectrum of **27** displayed a higher number of resonances than that of **26**. Coupling patterns indicated a different substitution in one of the B-rings, while the other parts of the molecule displayed a pseudosymmetric arrangement. Based on HMBC correlations, the position of the additional OCH_3_ group was identified as C-3′, allowing us to assign compound **27** to 3′-methoxycupressuflavone (Figure 3). In the case of **29**, the low number of proton resonances supported the symmetric substitution pattern indicated by the MS/MS data. HMBC correlations enabled the assignment of two methoxy groups to positions 3″ and 3‴ in the two apigenin subunits, allowing us to identify the compound as 3′,3‴-dimethoxycupressuflavone (Figure 3). Compounds **27** and **29** have only been described once before by Jang et al. [43] reported the isolation of these compounds from *Zabelia tyaihyoni* (Nakai) Hisauti & H. Hara (Caprifoliaceae). Consequently, this is the first report of the isolation of these compounds from a Lonicera species.

Compounds **43**, **44**, **46** and **54** were obtained as amorphous white solids. HR-MS data indicated a molecular formula of C_36_H_68_O_9_ for **43**, **44** and **46**, and of C_38_H_72_O_9_ for **54**, and three double bond equivalents in all four constituents. In the ^1^H NMR spectrum of compound **46**, a terminal CH_3_ group (δ_H_ = 0.89 ppm) could be assigned. Broad signal groups between 1.30 and 1.60 ppm indicated the presence of a saturated alkyl chain with widely overlapping proton signals (Supplementary Figure S16). The HSQC spectrum (Supplementary Figure S17) determined the presence of five hydroxymethine groups. Two of these were found to bear acetyl moieties, the methyl group of which appeared as sharp singlet at a shift of 2.02 ppm in the proton spectrum. HMBC correlations indicated the presence of three carbonyl functional groups; two of these were assigned to the acetyl moieties (δc = 172.9 ppm) and the third to the carboxyl moiety at position 1 (δc = 176.9 ppm) (Supplementary Figure S18 and Table 4). Starting from the carboxyl group at position 1, a CH_2_ group was assigned to position C-2 (δc = 43.7 ppm), and a CHOH group to position C-3 (δc = 69.6 ppm); via their HMBC correlations, the CH_2_ groups at position C-4 (δc = 38.4 ppm) and C-5 (δc = 22.4 ppm) could be assigned as well. The terminal CH_3_ group (δc = 14.5 ppm) displayed typical HMBC correlations for alkyl chains with a length of at least five carbon atoms. The remaining two CHOH groups showed only two HMBC correlations (δc = 38.1 ppm and 22.7 ppm). This indicates that the C atoms that couple to these two groups via two or three bonds display the same or a very similar chemical environment. For the two CHOCOCH_3_ groups, three HMBC correlations (δc = 35.3 ppm, 26.4 ppm and 22.2 ppm) were detectable, indicating a slightly different chemical environment for the two groups (Supplementary Figure S19). The nonoverlapping HMBC correlations for these four groups indicate that at least four CH_2_ groups are present; HSQC-TOCSY experiments did not provide useful data; thus, it was impossible to assign the exact position of the two CHOCOCH_3_ groups and the two remaining CHOH groups in the molecule by means of NMR spectroscopy. GC-MS experiments performed in a similar way as described by Seipold et al. [44] did not allow the unambiguous assignment of these functional groups either (data not shown).

In summary, compound **46** could be tentatively assigned as a saturated C_32_ fatty acid hydroxylated at position 3. There are two more OH groups and two OCOCH_3_ groups between position 3 and the terminal alkyl chain, which consists of at least five CH_2_ groups. The exact positions of these functional groups could not be determined.

^1^H NMR spectra of compounds **43**, **44** and **54** were highly similar to those of compound **46**, i.e., similar shifts were seen for the protons at positions 2, 3 and 32, as well as for the overlapping protons of the acetate groups, the CHOH groups and the acetylated CHOH groups (Supplementary Figures S20–S22). For **43** and **44**, LC-HRMS data indicated the same molecular formula as for **46**. Likewise, the MS/MS fragmentation patterns were highly similar. Therefore, **43** and **44** were assumed to be isomers of **46**, containing a saturated C_32_ alkyl chain as well. The molecular formula for compound **54** was calculated as C_38_H_72_O_9_, and, as for **43**, **44** and **46**, the MS/MS fragmentation pattern indicated the loss of three H_2_O and two CH_3_COOH molecules. Therefore, **54** obviously contains a saturated C_34_ chain with the same functional groups as **43**, **44** and **46**.

### 2.5. Several Candidate Compounds Displayed In Vitro Anti-Inflammatory Activity

In order to assess the potential activities anticipated from the OPLS-DA models, 15 candidates that were available as pure compounds were tested in the same cellular in vitro assays which had been used for extract screening. The test compound set comprised flavonoid glycosides and aglycones, biflavonoids, an iridoid glycoside, fatty acids and the chlorophyll degradation product pheophorbide A. Compounds were tested at a screening concentration of 30 µM; if inhibitory activity was identified, the IC_50_ values were determined (Table 5). Experimental results were compared with the priorities obtained from the respective OPLS-DA models (Table 6). 

In the OPLS-DA models, the flavone aglycone luteolin (**18**) was correlated with inhibitory activity in the NF-κB and IL-8 assay, and apigenin (**21**) was an NF-κB inhibitory candidate. The newly isolated 7-hydroxy-5,3′,4′,5′-tetramethoxyflavone (**22**) was indicated as potential IL-8 and NO inhibitor. Luteolin and apigenin indeed potently inhibited NF-κB activation (IC_50_ 6.84 and 4.11 µM) and moderately inhibited IL-8 production (IC_50_ 27.5 and 18.9 µM). Luteolin was only moderately active in the NO assay (IC_50_ 31.75 µM). 7-Hydroxy-5,3′,4′,5′-tetramethoxyflavone was inactive in all three models. The flavonol-*O*-glycoside luteolin-7-glucoside (**7**), which serves as a quality marker for Lonicerae japonicae flos according to the Chinese Pharmacopoeia [1], was also tested, although it was a candidate that was assigned low priority and reliability. The compound displayed no activity in any of the three assays. Five candidates with biflavonoid structure were subjected to pharmacological testing. While cupressuflavone (**26**) and 3′,3″-dimethoxycupressuflavone (**29**) were inactive in all three assays, 3′-methoxycupressuflavone (**27**), amentoflavone (**32**) and podocarpusflavone A (**33**) displayed moderate to high activity in the NF-κB activation assay (IC_50_ 19.6, 6.45 and 3.44 µM for **23**, **28** and **29**, respectively). Compound **33** also displayed a weak inhibitory effect on IL-8 expression (IC_50_ 47.88 µM). The predicted high priorities concerning inhibitory activity of compounds **26**, **27** and **29** on NO formation could not be verified by the experiments.

An iridoid was also among the candidate compounds, namely, secoxyloganin (**4**). However, its high priority on IL-8 expression could not be experimentally verified in this study. The chlorophyll breakdown product pheophorbide A (**52**) was correlated with inhibition of NF-κB activation, which could be experimentally verified (IC_50_ 3.04 µM). It also inhibited IL-8 production; however, this might be due to impaired HUVECtert cell viability.

Finally, several long-chain polyhydroxylated fatty acids represented high-ranked candidate compounds in particular with regard to their inhibition of IL-8 expression and NO formation. Four of these, compounds **43**, **44**, **46** and **54**, which had been isolated from *L. hypoglauca*, were subjected to pharmacological testing. Compounds **46** and **54** indeed displayed inhibitory activity: compound **46** in all three models and compound **54** in the IL-8 and NO assays. However, all four fatty acids also impaired the viability of the cells used in the respective assays (Table 5 and Table 6).

Taken together, the highest number of candidate compounds could be verified in the NF-κB transactivation assay. The most active compounds were flavonoid aglycones and biflavonoids. Pheophorbide A (**52**) and the newly identified long-chain polyhydroxy fatty acids (**46**, **54**) showed remarkable inhibitory activity both on NF-κB activation and NO formation, as well as IL-8 expression, respectively, but they also impaired the cell viability.

## 3. Discussion

In TCM, flowers and stems of *L. japonica* are most commonly used for anti-inflammatory therapies. In this study, we investigated leaf extracts from *L. japonica,* as well as from other Chinese Lonicera species that are less commonly used in TCM or not used at all. Besides *L. japonica* extracts, other less thoroughly investigated species also displayed considerable in vitro anti-inflammatory activity, such as *L. hypoglauca* and *L. acuminata*. Consequently, the leaves of these species may also be interesting sources for phytomedicines.

Annotation of the OPLS-DA candidate compounds on the basis of UHPLC-HRMS data and the structural elucidation of candidate compounds from *L. hypoglauca* after their isolation led to the tentative or complete identification of 65 compounds in total. Four of these were novel compounds (**43**, **44**, **46** and **54**), one was isolated for the first time from a natural source (**22**), and 28 compounds were identified in the genus *Lonicera* for the first time. Some of these may be new natural products. 

For several annotated candidates, anti-inflammatory activity had been previously reported.

Concerning the flavonoids, aglycones usually display higher activities in inflammation-related cell-based assays than their respective glycosides. In contrast, in animal models after oral treatment, the activity of glycosides has normally been shown to be similar or even higher than that of the respective aglycones [45]. The flavones luteolin and apigenin have shown potent in vitro and in vivo anti-inflammatory activity [46,47,48]. Luteolin-7-glucoside (cynaroside) has shown anti-inflammatory activity in septic [49] psoriatic animals [50]. These data underline the potentially important role of flavonoids and, in particular, of luteolin and its glycosides regarding the anti-inflammatory activity of Lonicera species. In our study, luteolin and apigenin displayed high anti-inflammatory activities, while luteolin-7-glucoside displayed no activity. In addition to these well-known flavonoids, 7-hydroxy-5,3′,4′,5′-tetramethoxyflavone could be isolated as a new natural product from *L. hypoglauca*. For this compound, only a weak inhibition of NF-κB activation was observed.

Biflavonoids such as the biapigenins ochnaflavone, podocarpusflavone A and amentoflavone have exhibited anti-inflammatory effects in vitro [51,52,53], while amentoflavone and cupressuflavone have shown these effects in animal models as well [54,55]. In this study, while cupressuflavone was inactive in all three assays, amentoflavone and podocarpusflavone A potently inhibited NF-κB activation. The rare biflavonoid 3′-methoxycupressuflavone moderately inhibited NF-κB activation, and podocarpusflavone A moderately inhibited IL-8 production. These findings indicate that biflavonoids are also relevant for the anti-inflammatory activity of Lonicera species.

Apart from these compound classes, which are well-known for Lonicera species, other activity-related candidates originated from compound classes that have been less commonly reported in Lonicera species or have not been previously described in this genus at all.

The chlorophyll degradation product pheophorbide A showed pronounced inhibition of NF-κB transactivation, a finding that is in accordance with literature data [56]. However, in contrast to literature reports [56], the compound did not inhibit NO production in RAW264.7 macrophages.

Moreover, we could isolate and partially identify the structures of four novel saturated C_32_- and C_34_-fatty acids containing different numbers of hydroxyl and acetyl moieties. Similar compounds, but with shorter chain lengths and lower numbers of hydroxy and acetyl substituents, have been identified in the floral oil of *Malphigia coccigera* [44]. There, the location of the substituents in the alkyl chains could be determined by interpreting GC-MS fragmentation patterns after methylation and trimethylsilylation. However, the same technique applied to our compounds that contained longer chains and more substituents led to ambiguous GC-MS fragments, which prevented the unequivocal detection of their substituent positions. Two of these displayed remarkable inhibitory activities in the applied cellular test models, but negatively impacted cell viability.

Further candidate compounds were identified from the compound classes of hydroxcinnamic acid derivatives, triterpenes, alkaloids and lipids. For many of these, the in vitro and in vivo anti-inflammatory effects have been previously described, but the absence of pure compounds prevented experimental assessments in this study. Thus, their role in the anti-inflammatory effects of the investigated extracts remains speculative.

Many of the annotated lipids reported in this study were detected in Lonicera species for the first time. They can be roughly stratified in three different categories: glycolipids, phospholipids and sphingolipids (glucocerebrosides and ceramides). One candidate compound was tentatively assigned to an acylglycerol bearing a sulfoquinovose. While diacylglyceryl sulfoquinovosides are associated with photosynthetic membranes of many photosynthetic organisms [57], palmitoyl glycerylsulfoquinovosides have only occasionally been isolated from natural sources, and their pharmacological activities have not been studied thoroughly [58].

Sphingolipids occur in essentially all eukaryotes and in certain prokaryotes. The glucocerebroside soyacerebroside II has been previously isolated from *L. japonica* [36]. An isomeric mixture of soyacerebrosides I and II has been shown to suppress the LPS-induced IL-18 release in human peripheral blood mononuclear cells [59]. 

Ceramides have been previously isolated from *L. japonica* [37]. However, their structures are not in concordance with those annotated in this study. Plant-derived ceramides are considered as interesting nutraceuticals [34], but their anti-inflammatory effects have not been systematically studied yet. Taking into consideration the fact that structural characteristics such as the number of hydroxyl groups determine whether a ceramide exhibits pro- or anti-inflammatory effects [60], the Lonicera ceramides annotated for the first time in this study may deserve a more thorough, systematic investigation to identify their structures and anti-inflammatory activity in the future.

In a subset of 15 OPLS-DA candidates that were available as pure compounds, the potential anti-inflammatory activities deduced from the models could be experimentally verified for several, but not for all, compounds. Some of the compounds exhibited not only anti-inflammatory activity but also impaired cell viability when applied as a pure substance. This side effect may be due to the fact that OPLS-DA-derived candidate compounds are typically among the more highly abundant bioactive compounds as a result of the high covariance criterion. In the complex, crude extracts used herein, obviously only the most powerful compounds are ranked highest; these, in turn, may also be toxic, while more easily tolerated, active trace compounds may remain unidentified [61].

To increase the identification rate of truly active compounds among MVDA-derived candidates and to unravel potential cytotoxic effects, it may be helpful to reduce the complexity of extracts by working with fractions in addition to or instead of crude extracts. For example, Kellogg et al. [44] suggested combining bioassay-guided fractionation with untargeted metabolite profiling by applying bio-chemometric methods. This approach enables the researcher to dereplicate the known bioactive compounds early on in the isolation process and to integrate several fractionation steps and the respective bioactivity data in one bio-chemometric analysis. Our results indicate that the successive addition of fractionation steps improved the quality of the biochemometric analysis [9]. Demarque et al. applied a partial least squares projection to latent structures and molecular networks to predict the presence of larvicidal compounds in pre-fractionated extracts from *Annona crassiflora*, then performed subsequent activity-guided fractionation to confirm this activity. According to these authors, crude extract prefractionation was a crucial step that enabled them to clean up the samples and to create unbalanced chemical profiles, thus facilitating the subsequent chemometric analysis of the samples [13].

## 4. Materials and Methods

### 4.1. Chemicals and Reagents

For HPLC-HRMS analyses, H_2_O was purified with a Barnstead EASYpure RF compact ultrapure water system, and CH_3_OH (VWR International, Rosny-sous-Bois-cedex, France) and HCOOH (Sigma Aldrich, Steinheim, Germany) were LC-MS grade. The other solvents were of analytical grade. Amentoflavone, luteolin and luteolin-7-glucoside were purchased from Carl Roth (Karlsruhe, Germany). Apigenin and pheophorbide A were purchased from Sigma Aldrich (Steinheim, Germany), and podocarpusflavone A, from Carbosynth (Berkshire, UK). Silica gel 60 for VLC (particle size 0.040–0.063 mm) and LiChroprep^®^ RP-18 (0.040–0.063 mm) for RP-18 column chromatography were purchased from Merck (Darmstadt, Germany). For solid-phase extraction, 5 g or 10 g Isolute^®^ C18 (EC) cartridges (Biotage, Uppsala, Sweden) were used.

### 4.2. Plant Material, Authentication and Extraction

Thirty-four (34) samples of the aerial parts of different *Lonicera* species were collected in the Guangxi Province (China) (samples 1–34). One sample of *L. japonica* flower buds was purchased at a TCM herb market in Nanning, China (sample 35), and one sample of *L. japonica* aerial parts was collected at the Botanical Garden in Graz (sample 36). Samples were authenticated by morphological analysis and by DNA barcoding as previously described [15]. Voucher specimens of all samples are deposited in the herbarium of the Guangxi Botanical Garden of Medicinal Plants (Nanning, China). A detailed sample list can be found in Supplementary Table S1. 

The dried leaves were ground, extracted with 96% ethanol by accelerated solvent extraction (ASE) as previously described [15] and dried by vacuum evaporation. Extracts were prepared in duplicates. For sample 34, only a single extract was prepared due to the low amount of plant material. Extract yields are provided in Supplementary Table S1. Samples were frozen at −20 °C. 

### 4.3. UHPLC-HRMS Metabolite Profiling and Data Processing

Dry extracts were dissolved in ethanol (5 mg/mL), and from each sample solution, 5 µL were injected. Subsequent to each six MS runs, a quality control sample (rutin, 1 mg/mL) was injected. UHPLC-HRMS analyses were performed on a Dionex Ultimate 3000 HPLC system hyphenated with a QExactive Hybrid Quadrupole Orbitrap MS (Thermo Fisher Scientific, Waltham, MA, USA). The stationary phase consisted of a Kinetex^®^ C18 column (1.7 µm, 100 Å, 2.1 × 100 mm, Phenomenex, Aschaffenburg, Germany), and the mobile phase was H_2_O + 0.4% HCOOH (A) and CH_3_OH + 0.4% HCOOH (B). The following parameters were applied: column temperature: 25 °C; flow rate: 0.2 mL/min; gradient: 0–6 min, 5–20% B in A; 6–25 min, 20–45% B in A; 25–50 min, 45–100% B in A; 50–60 min, 100% B; 60–61 min, 100–5% B in A; 61–71 min, re-equilibration. MS spectra were acquired in HESI-negative mode; probe heater temperature was set to 250 °C, capillary temperature to 350 °C, spray voltage to 3 kV, sheath gas flow to 30 arbitrary units, and auxiliary gas flow to 10 arbitrary units. 

Raw data files were processed with Compound Discoverer 3.1 (Thermo Scientific) (CD), using the following workflow: retention time window: 0–60 min; total intensity threshold: 12,000,000. Alignment was performed with adaptive curve model. Maximum RT shift was 2 min, and maximum mass tolerance 5 ppm. For detecting and grouping unknown compounds, S/N threshold: 3; minimum intensity threshold: 12,000,000; RT tolerance: 1 min; S/N threshold for gap filling: 20. Processing resulted in a data matrix consisting of retention time and intensity of every feature in every sample that was exported for further data treatment and subsequent multivariate data analysis.

### 4.4. Cellular In Vitro Assays

Assays were performed as previously described [15,62]. Extracts were dissolved in dimethyl sulfoxide (DMSO) and further diluted in cell culture medium to obtain a final concentration of 50 µg/mL; DMSO at the respective final concentration (<0.1%) was used as vehicle control in each assay. OPLS-DA candidate compounds were also dissolved in DMSO to obtain a final screening concentration of 30 µM. For compounds active at this concentration, IC_50_ values were determined. 

Transactivation activity of a NF-κB-driven luciferase reporter gene was determined in TNF-α (2 ng/mL)-stimulated HEK293/NF-κB-luc cells (Panomics, RC0014), using 5 µM parthenolide (Sigma-Aldrich, St. Louis, MO, USA) as positive control. Differences in cell viability were detected by comparing the fluorescence of vehicle-treated cells and cells treated with sample solutions upon staining with CellTracker Green CMFDA (C2925; Invitrogen), a fluorescent probe that is retained inside living cells [63]. Screening samples were tested in three independent experiments, each performed in quadruplicate.

Release of nitric oxide (NO) from RAW 264.7 macrophages stimulated with LPS (0.5 µg/mL; *E. coli* 055:B5; Sigma-Aldrich) and interferon-γ (IFNγ; 50 U/mL, mouse recombinant *E. coli*, Roche Diagnostics) were tested as previously described [64], using the non-specific NOS-inhibitor L-NMMA at 100 µM as the positive control. After removing 50 µL of the supernatants to react with Griess reagent (Sigma Aldrich), cells were incubated with XTT reagent (XTT proliferation kit II, Sigma Aldrich). NO production (Griess reaction) and metabolic activity of the cells (XTT formazan product) were photometrically measured. Tests were performed in three independent experiments, each in duplicate. 

Effects on IL-8 expression were tested in hTERT-immortalized HUVECtert cells stimulated with lipopolysaccharide (LPS, 100 ng/mL, Sigma-Aldrich), with BAY 11-7082 (5 µM) as positive control [15,62]. Activity screening was performed in sextuplicate. Impact of test samples on cell viability was determined after 6 h of stimulation by XTT. Incubation medium was removed, and 40 µL serum-free medium, containing XTT (Thermo Fisher Scientific, final concentration 200 µg/mL) and phenazine methyl sulfate (TCI Europe N.V., final concentration: 25 µM) were added to each well. After incubation for 2 h, absorbance was measured at 450 nm. Samples decreasing cell viability by more than 30% were considered to negatively influence cell viability.

To determine the IC_50_ values, pure compounds showing significant inhibitory activity at the screening concentration were tested at 5–10 concentrations, each with 4 replicates. IC_50_ values were calculated by applying the four-parameter logistic curve algorithm in Sigma Plot 13.0 (Systat Software Inc., Palo Alto, CA, USA) or GraphPad Prism 4.03 (GraphPad Software, Inc., San Diego, CA, USA).

### 4.5. Data Treatment and Multivariate Data Analysis (MVDA)

In order to compensate shifts in the sensitivity of the MS detector that possibly occurred in the course of the UHPLC-HRMS sequence, preprocessed UHPLC-HRMS data were normalized to the peak area of the external QC sample that had been injected after every series of six runs. Preprocessed, normalized UHPLC-HRMS data were imported to SIMCA 16.0.1 (Sartorius Stedim Data Analytics AB, Umeå, Sweden), pareto-scaled and log-transformed. Prior to OPLS-DA, bioactivity data were classified (active, medium-active and inactive; classification details are given in Supplementary Table S2). Moderately active samples were excluded from OPLS-DA models. To assess the reliability of the OPLS-DA models and to test the risk of overfitting, a cross-validated analysis of variance (CV-ANOVA) was performed [65]. Variables most likely correlated with bioactivity were deduced from the S-plots of the OPLS-DA models, and their reliability was deduced from the jackknife-based confidence interval in the loading plot of the S-plot [17]. Samples containing high levels of the candidate compounds were identified from the Xvar plots of the respective variables.

### 4.6. Annotation of OPLS-DA Candidates

OPLS-DA candidate features were annotated either by comparing their retention time, molecular formula and MS/MS fragmentation pattern with authentic reference standards (unambiguous assignment) or by comparing the data with data from the literature or mass spectral databases (tentative annotation). In cases where MS/MS fragmentation was not sufficient for tentative annotation, samples were additionally analyzed with the same UHPLC instrument and parameters as described above but while using the ion trap mass spectrometer (LTQ XL linear ion trap mass spectrometer equipped with H-ESI II probe, Thermo Fisher Scientific), which supports MS^n^ experiments. Analyses were performed in the HESI-negative mode. Heater temperature was 250 °C, capillary temperature was 330 °C, sheath gas flow was 20 and aux gas flow 8 arbitrary units. Source voltage was 3 kV, capillary voltage −48 V and tube lens −147 V. Normalized collision energy for data-dependent MS^2^ and MS^2^ was 35, activation Q was 0.25 and activation time was 30 ms.

### 4.7. Isolation and Structure Elucidation of OPLS-DA Candidates

Aerial parts of *L. hypoglauca* were collected on 17 July 2013 in Guangxi Province, China and authenticated as described in Section 2.2. Voucher specimens are kept in the herbarium of Guangxi Botanical Garden of Medicinal Plants (GXMG, Nanning, China) and at the Department of Pharmacognosy in Graz (Austria) (voucher specimen Nr. L-102). One and a half kilograms of the dried, powdered material were extracted by percolation with CH_3_OH at room temperature. The extract was concentrated on a rotary evaporator and dried under nitrogen to yield 113 g (7.5%) CH_3_OH extract. 

Of this, 108 g of the dried extract were subjected to liquid–liquid extraction, and the ethyl acetate subfraction was fractionated by performing several vacuum liquid chromatography (VLC) and solid-phase extraction (SPE) separation steps. Compounds were purified by means of semipreparative HPLC. The details of the isolation procedure are provided in the Supplementary Information. Taken together, 1.8 mg of compound **22**, 1.8 mg of compound **26**, 2.6 mg of compound **27**, 1.4 mg of compound **29**, 1.3 mg of compound **43**, 1.8 mg of compound **44**, 6.0 mg of compound **46** and 4.5 mg of compound **54** were isolated.

NMR spectra were recorded on a Bruker Avance III 700 MHz instrument; pyridine-d5 or MeOH-d4 (Eurisotop, France) were used as solvents; spectra were recorded at 25 °C; chemical shifts δ are relative to Me4Si as internal standard, *J* is in Hz. 

#### Experimental Data

**7-hydroxy-5-methoxy-2-(3**,**4**,**5-trimethoxyphenyl)-chromen-4-one (7-hydroxy-3**′,**4**′,**5**′**-tetramethoxyflavone**, **22).** Yellow powder. UV (MeOH): 222 (100%), 269 (65%), 310 sh, 330 (75%). ^1^H-NMR (700 MHz) and ^13^C-NMR (175 MHz): see Table 3. HR-ESIMS: 357.0959 ([M-H]^−^; C_19_H_17_O_7_^−^; calc: 357.0974); MS/MS: 342.0731 ([M-H-CH_3_]^−^; C_18_H_14_O_7_; calc. 342.0740); 327.0482 ([M-H-2CH_3_]^−^; C_17_H_11_O_7_; calc. 327.0505); 299.0563 ([M-H-2CH_3_-CO]^−^; C_16_H_11_O_6_; calc. 299.0556).

**5**,**7**,**5**′,**7**,**-Tetrahydroxy-2**,**2**′**-bis-(4-hydroxy-phenyl)-[8**,**8**′**]bichromenyl-4**,**4**′**-dione (cupressuflavone**, **26).** Yellow powder $\left[\alpha \right]\begin{array}{c} 25\\ D \end{array}=-156\;\left(c=0.08,\;CH3OH\right);$ UV (MeOH): 27 (1.7) 329 (1.5). ^1^H-NMR (700 MHz) and ^13^C-NMR (175 MHz): See Supplementary Table S5. HR-ESIMS: 537.0822 ([M-H]^−^; C_30_H_17_O_10_^−^; calc: 537.0822); MS/MS: 375.0496 (retro-Diels–Alder fragment [M-H-C_9_H_6_O_3_]^−^; C_21_H_11_O_7_^−^; calc: 375.0505).

**5**,**7**,**5**′,**7**,**-Tetrahydroxy-2-(4-hydroxy3-methoxy-phenyl)-2**′**-(4-hydroxy-phenyl)- [8**,**8**′**]bichromenyl-4**,**4**′**-dione (3**′**-methoxycupressuflavone**, **27).** Yellow powder. $\left[\alpha \right]\begin{array}{c} 25\\ D \end{array}=-68\;\left(c=0.18,\;CH3OH\right);$ UV (MeOH): 273 (1.3) 336 (1.2). ^1^H-NMR (700 MHz) and ^13^C-NMR (175 MHz): see Supplementary Table S5. HR-ESIMS: 567.0931 ([M-H]^−^; C_31_H_19_O_11_^−^; calc: 567.0927); MS/MS: 405.0598 (retro-Diels–Alder fragment subunit I [M-H-C_9_H_6_O_3_]^−^; C_22_H_13_O_8_^−^; calc: 405.0610), 375.0494(retro-Diels–Alder fragment subunit II [M-H-C_10_H_8_O_4_]^−^; C_21_H_11_O_7_^−^; calc: 375.0505).

**5**,**7**,**5**′,**7**,**-Tetrahydroxy-2**,**2**′**-bis-(4-hydroxy-3-methoxy-phenyl)-[8**,**8**′**]bichromenyl-4**,**4**′**-dione (3**′,**3**‴**-dimethoxycupressuflavone**, **29).** Yellow powder. $\left[\alpha \right]\begin{array}{c} 25\\ D \end{array}=-121\;\left(c=0.07,\;CH3OH\right);$ UV (MeOH): 274 (1.3) 344 (1.35). ^1^H-NMR (700 MHz) and ^13^C-NMR (175 MHz): see Supplementary Table S5. HR-ESIMS: 597.1041 ([M-H]^−^; C_32_H_21_O_12_^−^; calc: 597.1033); MS/MS: 405.0601 (retro-Diels–Alder fragment [M-H-C_10_H_8_O_4_]^−^; C_22_H_13_O_8_^−^; calc: 405.0610).

**Compounds 43 and 44:** white amorphous solids, UV (MeOH no absorption); ^1^H-NMR (700 MHz) spectra see Supplementary Figures S21 and S22. HR-ESIMS (neg): 643.4804 ([M-H]^−^ C_36_H_67_O_9_; calc: 643.4785); MS/MS: 583.4604 [M-H-CH_3_COOH]^−^; 59.0125 [CH_3_COO]^−^; HR-ESIMS (pos): 645.4880 ([M+H]^+^ C_36_H_69_O_9_; calc: 645.4942); MS/MS (main fragments): 507.4407 [M+H-2CH_3_COOH-H_2_O]^+^; 489.4295 [M+H-2CH_3_COOH-2H_2_O]^+^; 471.4190 [M+H-2CH_3_COOH-3H_2_O]^+^.

**Compound 46:** white amorphous solid, UV (MeOH) no absorption; ^1^H-NMR (700 MHz) and ^13^C-NMR (175 MHz)**: Table 4**; HR-ESIMS (neg): 643.4804 ([M-H]^−^ C_36_H_71_O_9_; calc: 643.4785); MS/MS: 583.4604 [M-H-CH_3_COOH]^−^; 59.0125 [CH_3_COO]^−^; HR-ESIMS (pos): 645.4880 ([M+H]^+^ C_36_H_69_O_9_; calc: 645.4942); MS/MS (main fragments): 507.4407 [M+H-2CH_3_COOH-H_2_O]^+^; 489.4295 [M+H-2CH_3_COOH-2H_2_O]^+^; 471.4190 [M+H-2CH_3_COOH-3H_2_O]^+^.

**Compound 54:** white amorphous solid, UV (MeOH no absorption); ^1^H-NMR (700 MHz) spectrum see Supplementary Figure S22. HR-ESIMS (neg): 671.5063 ([M-H]^−^ C_38_H_71_O_9_; calc: 671.5098); MS/MS: 611.4861 [M-H-CH_3_COOH]^−^; 59.0119 [CH_3_COO]^−^; HR-ESIMS (pos): 673.5253 ([M+H]^+^ C_38_H_73_O_9_; calc: 673.5255); MS/MS (main fragments): 535.4731 [M+H-2CH_3_COOH-H_2_O]^+^; 517.4624 [M+H-2CH_3_COOH-2H_2_O]^+^; 499.4509 [M+H-2CH_3_COOH-3H_2_O]^+^.

## 5. Conclusions

Correlating pharmacological activity and UHPLC-HRMS data by means of OPLS-DA enabled us to narrow down the incredibly broad and diverse spectrum of secondary metabolites present in the investigated *Lonicera* species to a panel of candidates with putative pharmacological activity that could subsequently be annotated and further investigated. Among these candidates, we identified several new natural products and compounds that had been previously undiscovered in the genus *Lonicera*. In a set of tested candidate compounds, pharmacological activities could be verified in some but not in all cases. These findings confirm that the applied MVDA approach is feasible, enabling researchers to preselect a panel of potentially active candidates in active herbal extracts. However, when this approach is applied to complex crude extracts, the most potent and thus potentially toxic candidates fall predominantly within the top ranks. Therefore, we anticipate that this approach will need to be optimized by fractionating *Lonicera* extracts rich in putative anti-inflammatory active compounds, accompanied by pharmacological testing, UHPLC-HRMS analysis, chemometric methods and molecular networks.

## Figures and Tables

**Table 1:** OPLS-DA model parameters and *p*-values of CV ANOVA of the OPLS-DA models generated from CD-processed LC-HRMS data and pharmacological assay data classified according to the parameters given in Supplementary Table S2 (extracts classified as moderately active were excluded from the models). *A*: number of principal components; *N*: number of observations; *R2X* (*cum*): cumulative sum of squares of the entire *X* explained by all extracted components. *R2Y* (*cum*): cumulative sum of squares of all the y-variables explained by the extracted components. *Q2* (*cum*): cumulative *Y* variation predicted by the *X* model for the extracted components, according to cross-validation.

Assay	OPLS-DA Model Parameters					*p*-Value of CV ANOVA
	*A*	*N*	R2X (*cum*)	R2Y (*cum*)	Q2 (*cum*)	
**NF-κB**	1 + 3 + 0	51	0.384	0.959	0.837	3.45 × 10^−14^
**IL-8**	1 + 4 + 0	57	0.409	0.984	0.894	4.75 × 10^−19^
**NO**	1 + 6 + 0	57	0.540	0.996	0.978	3.99 × 10^−30^

**Table 2:** List of annotated OPLS-DA candidates; compounds written in italics were isolated from *L. hypoglauca*, and their structures were elucidated fully or partially by NMR spectroscopy (Section 2.4); priority: deduced from S-plots of respective OPLS-DA models; low number represents high priority; numbers written in gray represent candidates with low levels of reliability (jackknife-based confidence interval, including 0); annotation: phospholipid and sphingolipid nomenclature, see Reference [18], fragmentation patterns and identification sources and Supplementary Table S4. ID level ([19,20]): ^0^, isolated pure compound, unambiguous 3D structure; ^1^, confident 2D structure, identified by reference standard match (retention time and MS/MS); ^2^, tentative annotation by comparison of structural formula and MS/MS fragmentation pattern with data from databases or literature. ^3^, no data available in literature or databases; compound tentatively assigned on the basis of the MS/MS fragmentation pattern (theoretical interpretation and/or comparison to related compounds).

Nr	Monoisotopic Mass (Calculated)	RT (min)	*m*/*z* (Experimental)	Molecular Formula	∆ (ppm)	IL-8 Priority	NFκB Priority	NO Priority	Annotation ^ID level^	Compound Class
**1**	180.042	10.71	179.033	C_9_H_8_O_4_	−3.5		**8**		**caffeic acid ^2^**	hydroxycinnamic acid derivative
**2**	338.100	12.63	337.092	C_16_H_18_O_8_	1.7			**12**	**coumaroylquinic acid ^2^**	hydroxycinnamic acid derivative
**3**	338.100	13.42	337.093	C_16_H_18_O_8_	2.2			**20**	**coumaroylquinic acid ^2^**	hydroxycinnamic acid derivative
**4**	404.132	15.62	403.124	C_17_H_24_O_11_	1.5	**9**			**Secoxyloganin ^1^**	iridoid glycoside
**5**	610.153	18.27	609.145	C_27_H_30_O_16_	0.7		**30**		**luteolin-dihexoside ^2^**	flavonoid-O-glycoside
**6**	580.143	19.99	579.134	C_26_H_28_O_15_	1.7	**10**	**13**		**luteolin-hexoside-pentoside ^2^**	flavonoid-O-glycoside
**7**	448.101	20.91	447.092	C_21_H_20_O_11_	−0.9		**27**		**luteolin-7-*O*-glucoside ^1^**	flavonoid-O-glycoside
**8**	594.159	21.59	593.150	C_27_H_30_O_15_	0.0		**15**	**2**	**kaempferol-3-hexoside-7-deoxyhexoside ^2^**	flavonoid-O-glycoside
**9**	478.111	21.99	477.104	C_22_H_22_O_12_	1.7			**26**	**isorhamnetin-7-*O*-hexoside ^2^**	flavonoid-O-glycoside
**10**	594.159	22.00	593.150	C_27_H_30_O_15_	−0.3	**25**			**lonicerin (luteolin hexoside deoxyhexoside) isomer ^2^**	flavonoid-O-glycoside
**11**	516.127	22.19	515.189	C_25_H_24_O_12_	0.1	**24**			**dicaffeoylquinic acid ^2^**	hydroxycinnamic acid derivative
**12**	516.127	24.18	515.118	C_25_H_24_O_12_	−1.5		**11**		**dicaffeoylquinic acid ^2^**	hydroxycinnamic acid derivative
**13**	448.101	25.16	447.092	C_21_H_20_O_11_	0.6		**2**		**kaempferol-3-hexoside (astragalin isomer) ^2^**	flavonoid-O-glycoside
**14**	478.111	25.50	477.104	C_22_H_22_O_12_	1.6			**30**	**isorhamnetin-3-*O*-hexoside ^2^**	flavonoid-O-glycoside
**15**	484.137	26.69	483.129	C_25_H_24_O_10_	1.8			**22**	**3,5-di-*O*-*p*-coumaroylquinic acid ^2^**	hydroxycinnamic acid derivative
**16**	610.132	28.94	609.124	C_30_H_26_O_14_	0.8			**29**	**luteolin-*O*-caffeoyl-*O*-hexoside ^3^**	flavonoid-O-glycoside derivative
**17**	484.137	29.84	483.129	C_25_H_24_O_10_	0.8			**16**	**4,5-di-*O*-*p*-coumaroylquinic acid ^2^**	hydroxycinnamic acid derivative
**18**	286.048	30.06	285.040	C_15_H_10_O_6_	2.3	**28**	**3**		**luteolin ^1^**	flavonoid aglycone
**19**	316.058	30.89	315.050	C_16_H_12_O_7_	2.1	**39**		**21**	**isorhamnetin ^2^**	flavonoid aglycone
**20**	594.137	31.73	593.129	C_30_H_26_O_13_	0.7			**27**	**luteolin-*O*-coumaroyl-*O*-hexoside ^3^**	flavonoid-O-glycoside derivative
**21**	270.053	32.50	269.045	C_15_H_10_O_5_	2.7		**25**		**apigenin ^1^**	flavonoid aglycone
***22***	*358.105*	*32.64*	*357.098*	*C_19_H_18_O_7_*	*3.0*	***8***		***7***	***7-hydroxy-5*,*3*′,*4*′,*5*′*-tetramethoxyflavone*^0^**	*flavonoid aglycone*
**23**	300.063	32.79	299.056	C_16_H_12_O_6_	1.6		**26**		**diosmetin/chrysoeriol ^2^**	flavonoid aglycone
**24**	328.225	33.76	327.217	C_18_H_32_O_5_	−0.7		**23**		**trihydroxyoctadecadienoic acid isomer I ^2^**	fatty acid
**25**	328.225	33.98	327.217	C_18_H_32_O_5_	−0.8	**19**	**1**		**trihydroxyoctadecadienoic acid isomer II ^2^**	fatty acid
***26***	*538.090*	*35.07*	*537.083*	*C_30_H_18_O_10_*	*1.6*		***17***	***1***	***cupressuflavone* ^0^**	*biflavonoid*
***27***	*568.101*	*35.29*	*567.093*	*C_31_H_20_O_11_*	*1.1*	***6***	***14***	***6***	***3*′*-methoxycupressuflavone*^0^**	*biflavonoid*
**28**	330.241	35.42	329.233	C_18_H_34_O_5_	−0.9		**32**		**trihydroxyoctadecenoic acid ^2^**	fatty acid
***29***	*598.111*	*35.51*	*597.104*	*C_32_H_22_O_12_*	*1.5*	***36***	***20***	***10***	***3*′*,3*′′*-dimethoxy-cupressuflavone*^0^**	*biflavonoid*
**30**	928.503	36.92	973.502 [M+HCOO]-	C_47_H_76_O_18_	1.4	**2**			**Akebia saponin D ^2^**	triterpene glycoside
**31**	444.205	36.99	443.197	C_27_H_28_O_4_N_2_	−0.5		**9**		**aurantiamide acetate ^2^**	alkaloid
**32**	538.090	37.24	537.082	C_30_H_18_O_10_	−0.9		**4**	**31**	**amentoflavone ^1^**	biflavonoid
**33**	552.106	40.28	551.098	C_31_H_20_O_10_	−0.5		**18**	**25**	**podocarpusflavone A ^1^**	biflavonoid
**34**	538.0900	40.96	537.081	C_30_H_18_O_10_	−2.5		**12**		**hinokiflavone ^2^/ochnaflavone ^2^**	biflavonoid
**35**	538.09	41.43	537.082	C_30_H_18_O_10_	−0.4		**29**		**hinokiflavone ^2^/ochnaflavone ^2^**	biflavonoid
**36**	294.430	41.93	293.212	C_18_H_30_O_3_	2.8		**5**		**oxo-octadecadienoic acid ^2^**	fatty acid
**37**	766.450	41.95	765.440	C_41_H_66_O_13_	−2.8	**34**			**Akebia saponin C ^2^**	triterpene glycoside
**38**	604.398	42.77	603.389	C_35_H_56_O_8_	−2.0	**16**			**Akebia saponin PA ^2^**	triterpene glycoside
**39**	676.367	43.50	721.363 [M+HCOO]^−^	C_33_H_56_O_14_	−0.7	**26**			**gingerglycolipid A isomer ^2^**	glycolipid
**40**	517.317	44.07	562.314 [M+HCOO]^−^	C_26_H_48_NO_7_P	−1.6	**23**			**LPC 18:3 ^2^**	phospholipid
**41**	602.476	47.19	601.468	C_34_H_66_O_8_	0.3	**5**	**14**	**4**	**trihydroxy-monoacetoxy-dotriacontanoic acid I ^3^**	fatty acid
**42**	602.476	47.55	601.468	C_34_H_66_O_8_	0.4	**15**		**13**	**trihydroxy-monoacetoxy-dotriacontanoic acid II ^3^**	fatty acid
***43***	*644.486*	*47.96*	*643.469*	*C_36_H_68_O_9_*	*0.6*	***6***	***22***	***5***	***trihydroxy-diacetoxy-dotriacontanoic acid I* ^2^**	*fatty acid*
***44***	*644.486*	*48.24*	*643.479*	*C_36_H_68_O_9_*	*0.4*	***17***	***31***	***9***	***trihydroxy-diacetoxy-dotriacontanoic acid II* ^2^**	*fatty acid*
**45**	482.265	48.42	481.256	C_22_H_43_O_9_P	−0.4	**35**			**LPG 16:1 ^2^**	phospholipid
***46***	*644.486*	*48.45*	*643.479*	*C_36_H_68_O_9_*	*0.1*	***11***	***6***	***3***	***trihydroxy-diacetoxy-dotriacontanoic acid III* ^2^**	*fatty acid*
**47**	630.507	48.60	629.499	C_36_H_70_O_8_	1.2	**32**		**14**	**tetrahydroxy-monoacetoxy-tetratriacontanoic acid I ^3^**	fatty acid
**48**	556.291	48.91	555.283	C_25_H_48_O_11_S	−0.7		**21**		**palmitoyl-sulfoquinovosyl-glycerol ^2^**	glycolipid
**49**	630.507	48.96	629.499	C_36_H_70_O_8_	1.1	**13**		**11**	**tetrahydroxy-monoacetoxy-tetratriacontanoic acid II ^3^**	fatty acid
**50**	686.497	49.16	685.489	C_38_H_70_O_10_	1.3	**40**		**18**	**dihydroxy-triacetoxy-dotriacontanoic acid ^3^**	fatty acid
**51**	672.518	49.24	671.510	C_36_H_72_O_9_	0.6			**17**	**trihydroxy-diacetoxy- tetratriacontanoic acid I ^3^**	fatty acid
**52**	592.269	49.32	591.260	C_35_H_36_N_4_O_5_	−0.0		**7**		**pheophorbide A ^1^**	chlorophyll breakdown product
**53**	672.518	49.52	671.510	C_38_H_72_O_9_	1.5	**31**		**15**	**trihydroxy-diacetoxy-tetratriacontanoic acid II ^3^**	fatty acid
***54***	*672.518*	*49.75*	*671.510*	*C_38_H_72_O_9_*	*1.3*		***10***	***6***	***trihydroxy-diacetoxy-tetratriacontanoic acid III* ^2^**	*fatty acid*
**55**	484.280	49.77	483.272	C_22_H_45_O_9_P	0.5	**7**			**LPG 16:0 ^2^**	phospholipid
**56**	714.528	50.34	713.521	C_40_H_74_O_10_	1.4			**24**	**dihydroxy-triacetoxy-tetratriacontanoic acid ^3^**	fatty acid
**57**	713.544	51.99	712.535	C_40_H_75_O_9_N	−2.0	**21**			**soyacerebroside isomer (HexCer 18:2;O2/16:0;O) ^2^**	glucocerebroside
**58**	384.360	52.47	383.354	C_24_H_48_O_3_	1.4		**19**		**hydroxytetracosanoic acid ^2^**	fatty acid
**59**	669.591	54.33	668.582	C_40_H_79_O_6_N	−0.8	**39**			**Cer 18:1;O3/22:0;O2 ^2^**	ceramide
**60**	653.596	55.44	652.587	C_40_H_79_O_5_N	−1.1	**14**			**Cer 18:1;O3/22:0;O ^2^**	ceramide
**61**	697.622	55.60	696.613	C_42_H_83_O_6_N	−1.1	**4**	**16**		**Cer 18:1;O3/24:0;O2 ^2^**	ceramide
**62**	667.612	56.15	780.595 [M+CF_3_COO]^−^	C_41_H_81_O_5_N	−1.1	**30**			**Cer 18:1;O3/23:0;O ^2^**	ceramide
**63**	681.627	56.95	680.618	C_42_H_83_O_5_N	−1.1	**1**			**Cer 18:1;O3/24:0;O ^2^**	ceramide
**64**	683.643	57.83	796.627 [M+CF_3_COO]^−^	C_42_H_85_O_5_N	−0.4	**20**			**Cer 18:0;O3/24:0;O ^2^**	ceramide
**65**	709.659	58.85	822.642 [M+CF_3_COO]^−^	C_44_H_87_O_5_N	−1.5	**37**			**Cer 18:1;O3/26:0;O ^2^**	ceramide

**Table 3:** ^1^H and ^13^C NMR shifts for compound **22** (pyridine-d_5_, 700 and 175 MHz, respectively); HMBC correlations, see Supplementary Figure S15.

Position	δ_C_, Type	δ_H_ (*J* in Hz)
2	160.3, C	-
3	109.2, CH	6.99 s
4	176.7, C	-
4a	108.6, C	-
5	161.9, C	-
6	97.8, CH	6., d (1.8)
7	164.6, C	-
8	96.5, CH	6.99, brs
8a	n. d.	-
1′	127.7, C	-
2′	104.4, CH	7.29, s
3′	154.2, C	-
4′	141.5, C	-
5′	154.2, C	-
6′	104.4, CH	7.29, s
5-CH_3_	56.22, CH_3_	3.87, s
3′-CH_3_	56.3, CH_3_	3.82, s
4′-CH_3_	60.7, CH_3_	3.94, s
5′-CH_3_	56.3, CH_3_	3.82, s

**Table 4:** ^1^H and ^13^C NMR shifts for compound **46** (MeOH-d_4_, 700 and 175 MHz, respectively).

Position	δ_C_, Type	δ_H_
1	176.9, C	-
2	43.7, CH_2_	2.36 m, 2.42 m
3	69.6, CH	3.97 m
4	38.2, CH_2_	1.49, 1.49
5	22.4	1.61, 1.61
29	30.7, CH_2_	n.d.
30	33.1, CH_2_	n.d.
31	23.8, CH_2_	1.31
32	14.5, CH_3_	0.89 t (6.6)
2 positions with CH_3_COO moieties	75.4, CH	4.85
2 CH_3_COO moieties	172.9, C 21.2, CH_3_	- 2.02 s
2 additional positions containing OH moieties	72.3, CH 72.3, CH	3.50 3.54

**Table 5:** Inhibitory activities of selected OPLS-DA hit compounds in cellular inflammation assays. ^#^ Compound numbers and identification; see Table 2. Effect on NF-κB activation was assessed in HEK/NFκB-luc cells stimulated with TNF-α; positive control: parthenolide (5 µM); results are given as means ± SD of 4 replicates (each in quadruplicate); IL-8 expression was assessed in HUVECtert cells stimulated with LPS; positive control: Bay 11-7082 (7.5 µM); results are given as means ± SD of 5 replicates; ^b^ in compounds significantly impairing cell viability, IL8 expression was determined one sample prepared by pooling the 5 replicates.; ^+^ compound significantly impaired cell viability at concentration of 30 µM; Effect on NO production was assessed in LPS/IFNγ-stimulated RAW macrophages. Extract concentration: 50 µg/mL; positive control: L-NMMA (100 µM); results are given as the means ± SD of 3 experiments performed in duplicates.

Compound Nr. ^#^	Identity	IL-8		NFκB		NO	
		% Inhibition (30 µM)	IC_50_ (µM)	% Inhibition (30 µM)	IC_50_ (µM)	% Inhibition (30 µM)	IC_50_ (µM)
**4**	**secoxyloganin**	−0.96 ± 6.01	nd	−17.25 ± 9.25	nd	5.85 ± 4.58	nd
**7**	**luteolin-7-glucoside**	7.42 ± 2.04	nd	0.68 ± 4.29	nd	4.68 ± 9.82	nd
**18**	**luteolin**	**74.84 ± 2.15**	**13.5**	**95.83 ± 0.01**	**6.84**	**56.39 ± 12.68**	**31.75**
**21**	**apigenin**	**64.79 ± 3.03**	**18.9**	**100.01 ± 0.01**	**4.11**	24.28 ± 10.85	nd
**22**	**7-hydroxy-5,3′,4′,5′-tetramethoxyflavone**	13.16 ± 2.49	nd	**51.11 ± 2.86**	nd	8.22 ± 11.91	nd
**26**	**cupressuflavone**	−2.05 ± 3.02	nd	3.42 ± 3.15	nd	9.61 ± 11.44	nd
**27**	**3′-methoxycupressuflavone**	3.50 ± 4.58	nd	**78.11 ± 8.86**	**19.6**	1.45 ± 7.78	nd
**29**	**3′, 3″-dimethoxycupressuflavone**	2.97 ± 9.92	nd	**31.25 ± 15.51**	nd	−0.85 ± 3.84	nd
**32**	**amentoflavone**	9.35 ± 2.78	nd	**90.83 ± 3.43**	**6.45**	7.06 ± 10.62	nd
**33**	**podocarpusflavone A**	**40.24 ± 29.78**	**47.88**	**99.23 ± 1.25**	**3.44**	3.42 ± 3.94	nd
**43**	**trihydroxy-diacetoxydotriacontanoic acid I**	3.84 ^+,b^	nd	−27.52 ± 9.75	nd	2.73 ± 6.84	nd
**44**	**trihydroxy-diacetoxydotriacontanoic acid II**	**31.36 ^+,b^**	nd	10.12 ± 12.01	nd	12.82 ± 8.10	nd
**46**	**trihydroxy-diacetoxydotriacontanoic acid III**	**84.92 ^+,b^**	nd	**66.25 ± 3.75 ^+^**	nd	**47.6 ± 14.62 ^+^**	nd
**52**	**pheophorbide A**	**95.8 ^+,b^**	nd	**74.68 ± 13.64**	**3.04**	5.48 ± 7.55	nd
**54**	**trihydroxy-diacetoxytetracontanoic acid II**	**89.32 ^+,b^**	nd	8.25 ± 13.74	nd	**78.35 ± 7.19 ^+^**	nd

**Table 6:** Comparison of experimentally determined in vitro anti-inflammatory activities of selected OPLS-DA candidate compounds with priorities deduced from OPLS-DA model; green fields indicate correctly predicted active compounds, and yellow fields indicate correctly predicted active compounds with negative impact on cell viability; Compound numbers, identification and priorities from OPLS-DA models; see Table 1. Numbers written in gray indicate low reliability (jackknife-based confidence interval including 0); activities determined in cellular inflammation assays; see Table 5. **i** = inactive; **a** = active; **a/ct** = active, but cytotoxic.

Nr	Identity	IL-8 Priority	Activity	NF-κB Priority	Activity	NO Priority	Activity
**4**	**secoxyloganin**	**9**	**i**	**-**	**i**	**-**	**i**
**7**	**luteolin-7-glucoside**	**-**	**i**	**27**	**i**	**-**	**i**
**18**	**luteolin**	**28**	**a**	**3**	**a**	**-**	**a**
**21**	**apigenin**	**-**	**a**	**25**	**a**	**-**	**i**
**22**	**7-hydroxy-5,3′,4′,5′-tetramethoxyflavone**	**8**	**i**	**-**	**a**	**7**	**i**
**26**	**cupressuflavone**	**-**	**i**	**17**	**i**	**1**	**i**
**27**	**3′-methoxycupressuflavone**	**-**	**i**	**24**	**a**	**8**	**i**
**29**	**3′,3″-dimethoxycupressuflavone**	**36**	**i**	**20**	**(a)**	**10**	**i**
**32**	**amentoflavone**	**-**	**i**	**4**	**a**	**31**	**i**
**33**	**podocarpusflavone A**	**-**	**a**	**18**	**a**	**25**	**i**
**43**	**trihydroxy-diacetoxydotriacontanoic acid I**	**6**	**i/ct**	**22**	**i**	**5**	**i**
**44**	**trihydroxy-diacetoxydotriacontanoic acid II**	**17**	**i/ct**	**31**	**i**	**9**	**i**
**46**	**trihydroxy-diacetoxydotriacontanoic acid III**	**11**	**a/ct**	**6**	**a/ct**	**3**	**a/ct**
**52**	**phaeophorbide A**		**a/ct**	**7**	**a**	**-**	**i**
**54**	**trihydroxy-diacetoxytetracontanoic acid III**		**a/ct**	**10**	**i**	**6**	**a/ct**

## Data Availability

UHPLC-HRMS raw data were deposited in MetaboLights [66] (Study Nr MTBLS3112; www.ebi.ac.uk/metabolights/MTBLS3112 (accessed on 1 March 2022).

## Supplementary Materials

The following supporting information can be downloaded at https://www.mdpi.com/article/10.3390/metabo12040288/s1: Table S1: List of Lonicera samples included in the metabolic profiling study, their collection dates and origins, and extract yields obtained by ASE extraction with 96% ethanol. Table S2: Classification of Lonicera methanolic extracts according to the observed in vitro pharmacological activities. Table S3: model window of PCA of preprocessed, normalized UHPLC-HRMS HESI-negative ion mode data (score scatter plot [t1]/[t2] shown in Figure 2). Table S4: Annotation of candidate compounds. Table S5: ^13^C and ^1^H NMR shifts of compounds **26**, **27**, and **29** and of apigenin (pyridine-d_5_, 700 and 175 MHz, respectively). Figure S1. Superposition of HESI-negative ion mode base peak chromatograms (*m*/*z* 133–2000) of five representative ethanolic leaf extracts from different Lonicera species. black: L3_1 (*L. hypoglauca*), red: L10_1 (*L. confusa*), blue: L32_1 (*L. acuminata*), green: L24_1 (*L. japonica*) and yellow: L15_1 (*L. macrantha*). Superposition of LC-HRMS raw data was generated using MzMine 2 (doi:10.1186/1471-2105-11-395). Figure S2: Superposition of UHPLC-HRMS HESI-negative ion mode base peak chromatograms (*m*/*z* 132–2000) of (A) all *Lonicera macrantha* samples (L12, 15, 16, 17, 21, 31 and 33); (B) *L. macrantha* samples of cluster 1 ([t1]/[t2] = −0.5/−0.5; samples L12, 15, 16 and 17) and; (C) *L. macrantha* samples of cluster 2 (t1]/[t2] = −0.5/+0.5; samples 31, 31 and 33) in a PCA score scatter plot [t1]/[t2] (Figure 2). Generated with MzMine 2 (doi:10.1186/1471-2105-11-395). Figure S3: Superposition of UHPLC-HRMS HESI-negative ion mode base peak chromatograms (*m*/*z* 132–2000) of (A) all *Lonicera japonica* samples (L23, 24, 25, 35 and 36) and (B) *L. japonica* leaf samples only (L23, 24, 25 and 36). Generated with MzMine 2 (doi:10.1186/1471-2105-11-395). Figure S4: OPLS-DA model of CD-processed UHPLC-HRMS data in correlation with activity on transactivation of an NF-κB-driven luciferase reporter gene in TNF-α-stimulated HEK293/NF-κB-luc cells. (A) Score scatter plot. Blue: highly active samples; green: samples with low activity; moderately active samples were excluded from the model. (B) S-Plot of the OPLS-DA model. Variables most likely correlated with bioactivity are marked in red and listed in Table 2 and Table S3. Figure S5: OPLS-DA model of CD-processed UHPLC-HRMS data in correlation with activity on the IL-8 expression in LPS-stimulated HUVECtert cells. (A) Score scatter plot. Blue: active samples; green: inactive samples; moderately active samples were excluded from the model. (B) S-Plot of the OPLS-DA model. Variables most likely correlated with bioactivity are marked in red and listed in Table 2 and Table S3. Figure S6: OPLS-DA model of CD-processed UHPLC-HRMS data in correlation with the activity on NO production in LPS/IFNγ-stimulated RAW 264.7 mouse macrophages. (A) Score scatter plot. blue: active samples; green: inactive samples; moderately active samples were excluded from the model. (B) S-Plot of the OPLS-DA model. Variables most likely correlated with bioactivity are marked in red and listed in Table 2 and Table S3. Figure S7: MS/MS spectra of LPC 18:3 (**40**), assigned with LDA. (A) Precursor: *m*/*z* 562.314 [M+HCOO]^−^, retention time: 44.18 min, file: Lonicera-L25-fl-1_1; (B) precursor *m*/*z* 502.290 [M-CH_3_]^−^, retention time: 44.08 min; file: Lonicera-L25-fl-1_1. Figure S8: MS/MS spectra of Cer 18:1;O3/22:0;O2 (**59**), assigned with LDA. (A) Precursor: *m*/*z* 668.580 [M-H]^−^, retention time: 54.32 min, file: Lonicera-jap-fl-1_1; (B) precursor *m*/*z* 714.590 [M+HCOO]^−^, retention time: 54.27 min, file: Lonicera-jap-fl-1_1. Figure S9: MS/MS spectra of Cer 18:1;O3/22:0;O (**60**), assigned with LDA. (A) Precursor: *m*/*z* 652.590 [M-H]^−^, retention time: 55.50 min, file: Lonicera-jap-fl-1_1; (B) precursor *m*/*z* 688.560 [M+Cl]^−^, retention time: 55.49 min; file: Lonicera-jap-fl-1_1. Figure S10: MS/MS spectra of Cer 18:1;O3/24:0;O2 (**61**), assigned with LDA. (A) Precursor: *m*/*z* 696.610 [M-H]^−^, retention time: 55.55 min, file: Lonicera-jap-fl-1_1; (B) precursor *m*/*z* 742.619 [M+HCOO]^−^, retention time: 55.64 min, file: Lonicera-jap-fl-1_1. Figure S11: MS/MS spectra of Cer 18:1;O3/23:0;O; (**62**) assigned with LDA. (A) Precursor: *m*/*z* 666.600 [M-H]^−^, retention time: 56.20 min, file: Lonicera-jap-fl-1_1; (B) precursor *m*/*z* 712.610 [M+HCOO]^−^ retention time: 56.18 min, file: Lonicera-jap-fl-1_1; (C) precursor: 702.580 [M+Cl]^−^, retention time: 56.19 min, file: Lonicera-jap-fl-1_1. Figure S12: MS/MS spectra of Cer 18:1;O3/24:0;O; (**63**), assigned with LDA. (A) Precursor: *m*/*z* 680.620 [M-H]^−^, retention time: 56.98 min; file: Lonicera-jap-fl-1_1; (B) precursor *m*/*z* 726.624 [M+HCOO]^−^, retention time: 56.98 min, file: Lonicera-jap-fl-1_1; (C) precursor: 716.598 [M+Cl]^−^, retention time: 56.92 min; file: Lonicera-jap-fl-1_1. Figure S13: MS/MS spectra of Cer 18:0;O3/24:0;O (**64**), assigned with LDA. (A) Precursor: *m*/*z* 682.630 [M-H]^−^, retention time: 57.95 min, file: Lonicera-jap-fl-1_1; (B) precursor *m*/*z* 728.640 [M+HCOO]^−^, retention time: 57.88 min, file: Lonicera-jap-fl-1_1; (C) precursor: *m*/*z* 718.610 [M+Cl]^−^, retention time: 57.73 min; file: Lonicera-jap-fl-1_1. Figure S14: MS/MS spectra of Cer 18:1;O3/26:0;O (**65**), assigned with LDA. (A) Precursor: *m*/*z* 708.650 [M-H]^−^, retention time: 58.88; file: Lonicera-jap-fl-1_1; (B) precursor *m*/*z* 754.650 [M+HCOO]^−^, retention time: 58.87 min; file: Lonicera-jap-fl-1_1; (C) precursor: *m*/*z* 744.627 [M+Cl]^−^, retention time: 58.86 min; file: Lonicera-jap-fl-1_1. Figure S15: HMBC correlations observed for compounds **22** and **26**. Figure S16: ^1^H NMR spectrum of compound 46 in MeOH-d_4_ (700 MHz, TMS, 298 K). Figure S17: HSQC spectrum of compound **46** in MeOH-d_4_ (TMS, 298 K). Figure S18: Correlations to carbonyl functions in the HMBC spectrum of compound **46** in MeOH-d4 (TMS, 298 K). Figure S19: Non-carbonyl HMBC correlations of CHOH and CHOCOCH3 groups in the HMBC spectrum of compound **46** in MeOH-d4 (TMS, 298 K). Figure S20: ^1^H NMR spectrum of compound **43** in MeOH-d_4_ (700 MHz, TMS, 298 K). Figure S21: ^1^H NMR spectrum of compound **44** in MeOH-d_4_ (700 MHz, TMS, 298K). Figure S22: ^1^H NMR spectrum of compound **54** in MeOH-d_4_ (700 MHz, TMS, 298 K).
