# Supplementary material for: Characterization of Constituents with Potential Anti-Inflammatory Activity in Chinese Lonicera Species by UHPLC-HRMS Based Metabolite Profiling
Source: Metabolites. 2022 Mar 25;12(4):288. doi: 10.3390/metabo12040288 (PMC9027581; doi:10.3390/metabo12040288)
Supplement: Supplementary file 1 [file metabolites-12-00288-s001.zip › metabolites-1637908-supplementary.pdf]

**Supplementary materials**

# Characterization of Constituents with Potential Anti-Inflammatory Activity in Chinese Lonicera Species by UHPLC-HRMS Based Metabolite Profiling

## Isolation procedure of OPLS-DA candidate compounds from *L. hypoglauca* methanolic extract

108 g of *L. hypoglauca* $CH_3OH$ extract were suspended in 500 ml $CH_3OH:H_2O$ 9:1 and subjected to subsequent liquid-liquid extraction with 4x 500 ml n-hexane, 4x 500 ml ethyl acetate (EtOAc) and 4x 500 ml n-butanol. The extracts were dried in vacuo to yield 10.2 g *n*-hexane subextract, 25.5 g EtOAc subextract (EA), 16.2 g n-butanol subextract and 54 g $H_2O$ residue.

25 g EA were subjected to VLC (vacuum liquid chromatography) on silica gel (300 g; column diameter 10 cm; stationary phase height 10 cm), using gradient elution with n-hexane- EtOAc – $CH_3OH$ mixtures of increasing polarity. VLC eluates were combined based on similarities in their TLC profiles to achieve 8 subfractions (EA1-8). EA3 (*n*-hexane: EtOAc:$CH_3OH$ 10:85:5, 2.55 g), EA4 (EtOAc:$CH_3OH$ 90:10, 6.30 g) and EA5 (EtOAc:$CH_3OH$ 90:10-75:25, 6.38 g) were separately dissolved in a mixture of $CH_3OH$ and $H_2O$ (9:1), and the solvent was gently evaporated by a rotary evaporator. As the $CH_3OH$ concentration decreases by evaporation, lipophilic constituents precipitate and attach to the wall of the round bottom flask, while the other compounds stay in solution, allowing to separate the more polar subfractions EA3_1, EA4_1 and EA5_1 from the non-polar subfractions EA3_2, EA4_2 and EA5_2. EA3_1 and EA4_1 were combined to EAH (3.05 g), and EA4_2, EA5_2 as well as EA6 were combined to EAL (7.5 g).

EAL was subjected to further silica gel VLC fractionation (100 g; column diameter 7 cm, stationary phase height 6.5 cm) using gradient elution with $CH_2Cl_2$ - $CH_3OH$ mixtures of increasing polarity. Eluates were combined to subfractions EAL_1-EAL-14. EAL_6 ($CH_2Cl_2$:$CH_3OH$ 95:5-94:6, 560 mg) was purified by solid phase extraction (SPE) on a 5 g cartridge with $CH_3OH:H_2O$ mixtures (40:60-100:0). EAL6_6 ($CH_3OH:H_2O$ 90:10) was separated by semipreparative HPLC on a Shimadzu HPLC system, using a LiChrospher® 100, RP-18 (10 µm) LichroCART® 250-10, and HPLC column (Merck) as stationary phase. Column

temperature was 25 °C and $CH_3CN$:$H_2O$ 33:67 at a flow rate of 3 ml/min was used as mobile phase. Separation furnished compound **22** (1.8 mg).

EAL_10 ($CH_2Cl_2$:$CH_3OH$ 91:9-98:11, 437 mg) was purified on a 10 g SPE cartridge using $CH_3OH$:$H_2O$ mixtures (50:50-100:0). EAL_10_8 ($CH_3OH$:$H_2O$ 85:15, 39 mg) was separated by semipreparative HPLC on a Luna® C18 (2) 10 µm 100 Å LC column (10x250 mm) (Phenomenex) at a column temperature of 40 °C. The gradient of the mobile phase comprised of solvents A ($H_2O$ + 0.2 % HCOOH) and B ($CH_3CN$/$CH_3OH$ 7/3 + 0.2% HCOOH), where B in A is gradually increased from 75 to 95 % in 0-40 min at a flow rate of 5 ml/min. By this approach, we isolated compound **43** (0.8 mg) and compound **44** (1.0 mg).

EAL_11 ($CH_2Cl_2$:$CH_3OH$ 89:11, 280 mg) was purified too by a 10 g SPE cartridge using MeOH:$H_2O$ mixtures (50:50-100:0). Purified subfraction EAL11_10 (MeOH:$H_2O$ 95:5, 38 mg) was separated by semipreparative HPLC with the same conditions as for EAL_10_8, yielding 1.9 mg of compound **54**.

EAL_12 and EAL_13 were combined, and fraction EAL_12/13 ($CH_2Cl_2$:$CH_3OH$ 86:14-50:50, 2.24 g) was fractionated by another VLC on silica gel (100 g, column diameter 3.5 cm, stationary phase height 10 cm). Mixtures of $CH_2Cl_2$:$CH_3OH$:$H_2O$ with increasing polarity (90:10:1-60:40:4) served as mobile phase, resulting in 24 subfractions. Subfraction EAL12/13_15 ($CH_2Cl_2$:$CH_3OH$:$H_2O$ 85:15:1.5 340 mg) was further purified on a 10 g SPE cartridge using $CH_3OH$:$H_2O$ mixtures (50:50-100:0). Purified subfraction EAL12/13_15_8 (MeOH/$H_2O$ 85:15, 39 mg) was separated by semipreparative HPLC using the same conditions as for EAL_10_8, furnishing further 0.5 mg of compound **43** and further 0.8 mg of compound **44,** and 6 mg of compound **46**. EAL12/13_15_9 ($CH_3OH$/$H_2O$ 90/10, 80 mg) was separated according to the same semipreparative HPLC protocol, allowing to isolate another 2.6 mg of compound **54**. Taken together, 1.8 mg of compound **22**, 1.3 mg of compound **43**, 1.8 mg of compound **44**, 6.0 mg of compound **46** and 4.5 mg of compound **54** have been isolated from EAL.

EAH was further separated by column chromatography on RP-18 material (36 g, column diameter 5.2 cm, stationary phase height 3 cm), with a $CH_3OH$/$H_2O$ gradient (10/90-90:10), resulting in subfractions EAH1-12. Subfraction EAH_11 ($CH_3OH$/$H_2O$ 70:30-80:20, 104 mg) was separated by semipreparative HPLC on the Luna® C18 column (column temperature: 25°C, mobile phase: $CH_3CN$ +0.1% HCOOH: $H_2O$ +0.1% HCOOH 35:65; flow rate: 4.0 ml/min). By this method, we isolated compound **26** (1.8 mg), **27** (2.6 mg), and **29** (1.4 mg).

**Supplementary Table S1:** List of Lonicera samples included in the metabolic profiling study, their species assignment, collection date and origin, and extract yields obtained by ASE extraction with 96 % ethanol.

| **Sample ID** | **Species Name** | **Collection date** | **Origin** | **Yield (%) Aliquot_1** | **Yield (%) Aliquot_2** |
|---|---|---|---|---|---|
| 1_1, 1_2 | *L. confusa* DC. | 2011-11-07 | Guangxi | 16.65 | 16.88 |
| 2_1, 2_2 | *L. hypoglauca* Miquel | 2011-11-07 | Guangxi | 19.36 | 18.61 |
| 3_1, 3_2 | *L. hypoglauca* Miquel | 2011-11-07 | Guangxi | 17.53 | 17.08 |
| 4_1, 4_2 | *L. confusa* DC. | 2011-11-08 | Guangxi | 18.18 | 18.03 |
| 5_1, 5_2 | *L. hypoglauca* Miquel | 2011-11-08 | Guangxi | 20.21 | 20.21 |
| 6_1, 6_2 | *L. confusa* DC. | 2011-11-08 | Guangxi | 21.30 | 20.83 |
| 7_1, 7_2 | *L. confusa* DC. | 2011-11-08 | Guangxi | 19.99 | 19.55 |
| 8_1, 8_2 | *L. similis* Hemsley | 2011-11-07 | Guangxi | 13.15 | 13.17 |
| 9_1, 9_2 | *L. hypoglauca* Miquel | 2011-11-09 | Guangxi | 19.39 | 19.57 |
| 10_1, 10_2 | *L. confusa* DC. | 2011-11-09 | Guangxi | 26.10 | 24.49 |
| 11_1, 11_2 | *L. confusa* DC. | 2011-11-09 | Guangxi | 32.29 | 32.46 |
| 12_1, 12_2 | *L. macrantha* (D. Don) Sprengel | 2011-11-04 | Guangxi | 32.55 | 32.05 |
| 13_1, 13_2 | *L. bournei* Hemsley | 2011-11-05 | Guangxi | 39.61 | 40.29 |
| 14_1, 14_2 | *L. hypoglauca* Miquel | 2011-11-09 | Guangxi | 12.37 | 12.20 |
| 15_1, 15_2 | *L. macrantha* (D. Don) Sprengel | 2011-11-04 | Guangxi | 21.43 | 21.24 |
| 16_1, 16_2 | *L. macrantha* (D. Don) Sprengel | 2011-11-11 | Guangxi | 15.67 | 15.74 |
| 17_1, 17_2 | *L. macrantha* (D. Don) Sprengel | 2011-11-11 | Guangxi | 14.15 | 14.21 |

| | | | | | |
|---|---|---|---|---|---|
| 18_1, 18_2 | L. similis Hemsley | 2011-11-11 | Guangxi | 12.99 | 12.76 |
| 19_1, 19_2 | *L. similis* Hemsley | 2011-11-13 | Guangxi | 14.33 | 14.45 |
| 20_1, 20_2 | *L. confusa* DC. | 2011-11-13 | Guangxi | 6.88 | 6.39 |
| 21_1, 21_2 | *L. macrantha* (D. Don) Sprengel | 2011-11-15 | Guangxi | 8.73 | 8.91 |
| 22_1, 22_2 | *L. reticulata* Champion | 2011-11-05 | Guangxi | 24.19 | 24.27 |
| 23_1, 23_2 | *L. japonica* Thunb. | 2011-11-05 | Guangxi | 17.06 | 17.35 |
| 24_1, 24_2 | *L. japonica* Thunb. | 2011-11-07 | Guangxi | 8.59 | 8.40 |
| 25_1, 25_2 | *L. japonica* Thunb. | 2011-11-07 | Guangxi | 7.33 | 7.55 |
| 26_1, 26_2 | *L. similis* Hemsley | 2011-11-10 | Guangxi | 13.89 | 14.02 |
| 27_1, 27_2 | *L. acuminata* Wallich | 2011-11-11 | Guangxi | 17.30 | 19.77 |
| 28_1, 28_2 | *L. reticulata* Champion | 2011-11-13 | Guangxi | 14.60 | 14.41 |
| 29_1, 29_2 | *L. acuminata* Wallich | 2011-11-13 | Guangxi | 10.30 | 10.13 |
| 30_1, 30_2 | *L. acuminata* Wallich | 2011-11-13 | Guangxi | 18.97 | 18.43 |
| 31_1, 31_2 | *L. macrantha* (D. Don) Sprengel | 2011-11-13 | Guangxi | 10.01 | 10.17 |
| 32_1, 32_2 | *L. acuminata* Wallich | 2011-11-16 | Guangxi | 15.50 | 15.30 |
| 33_1, 33_2 | *L. macrantha* (D. Don) Sprengel | 2011-11-09 | Guangxi | 8.20 | 8.22 |
| 34_1 | unidentified | 2011-11-09 | Guangxi | 12.90 | - |
| 35_1, 35_2 | *L. japonica* flower buds | NA | Purchased on TCM herb market | 31.16 | 31.22 |
| 36_1, 36_2 | *L. japonica* Thunb. | 2011-05-30 | Graz | 14.17 | 14.47 |

**Supplementary Table S2:** Classification of *Lonicera* methanolic extracts according to the observed in vitro pharmacological activities.

| Assay | Class | Activity | Readout | # samples |
|---|---|---|---|---|
| NO | 1 | Active | 0-0.3 relative units | 10 |
| | 2 | Inactive | 0.5->1 relative units | 47 |
| | 3 | Moderately active | 0.3-0.5 relative units | 14 |
| NF-κB | 1 | High activity | 0-0.1 relative units | 42 |
| | 2 | Low activity | 0.3->1 relative units | 9 |
| | 3 | Moderate activity | 0.1-0.3 relative units | 20 |
| IL-8 | 1 | Active | 0-0.3 relative units | 15 |
| | 2 | Inactive | 0.5->1 relative units | 42 |
| | 3 | Moderately active | 0.3-0.5 relative units | 14 |

**Supplementary Table S3:** model window of PCA of preprocessed, normalized UHPLC-HRMS HESI negative ion mode data (score scatter plot [t1]/[t2] shown in Figure 1)

| Component | R2X | R2X(cum) | Eigenvalue | Q2 | Limit | Q2(cum) | Significance | Iterations |
|---|---|---|---|---|---|---|---|---|
| | Cent. | | | | | | | |
| 1 | 0.147 | 0.147 | 10.4 | 0.114 | 0.0146 | 0.114 | R1 | 53 |
| 2 | 0.126 | 0.272 | 8.94 | 0.114 | 0.0148 | 0.215 | R1 | 34 |
| 3 | 0.0879 | 0.36 | 6.24 | 0.074 | 0.015 | 0.273 | R1 | 68 |
| 4 | 0.0767 | 0.437 | 5.44 | 0.0639 | 0.0152 | 0.32 | R1 | 84 |
| 5 | 0.0689 | 0.506 | 4.89 | 0.0817 | 0.0154 | 0.375 | R1 | 28 |
| 6 | 0.0475 | 0.553 | 3.37 | 0.0371 | 0.0156 | 0.398 | R1 | 200 |
| 7 | 0.0468 | 0.6 | 3.32 | 0.0482 | 0.0159 | 0.427 | R1 | 41 |
| 8 | 0.0375 | 0.638 | 2.66 | 0.0291 | 0.0161 | 0.444 | R1 | 93 |
| 9 | 0.0328 | 0.671 | 2.33 | 0.0278 | 0.0164 | 0.459 | R1 | 93 |
| 10 | 0.0308 | 0.701 | 2.19 | 0.0368 | 0.0166 | 0.479 | R1 | 90 |
| 11 | 0.0268 | 0.728 | 1.9 | 0.0127 | 0.0169 | 0.486 | R2 | 122 |
| 12 | 0.0248 | 0.753 | 1.76 | 0.03 | 0.0171 | 0.501 | R1 | 110 |
| 13 | 0.0231 | 0.776 | 1.64 | -0.00254 | 0.0174 | 0.5 | R2 | 67 |
| 14 | 0.0203 | 0.796 | 1.44 | 0.0378 | 0.0177 | 0.519 | R1 | 85 |

**Supplementary Table S4: Annotation of candidate compounds**

Compounds shown in italics have been isolated from *L. hypoglauca* and their structure has been fully or partially elucidated by NMR spectroscopy; [a]Nr: Identified compounds are numbered in the same way as in Table 2; unidentified compounds or adducts of identified compounds are not numbered; [b]RT: retention time in minutes; [c]*m/z*: *m/z* of the base peak, used for molecular formula prediction; [d]*m/z* (adduct): *m/z* of adduct used for LDA verification; [e]MS/MS: *m/z* values of MS/MS fragments (% of base peak intensity); priority: deduced from S-plots of respective OPLS-DA models; low number represents high priority; numbers written in grey represent candidates with low reliability (jack-knife based confidence interval including 0); ID levels [1][2]: 0, isolated pure compound, unambiguous 3D structure; 1, confident 2D structure, identified by reference standard match (retention time and MS/MS); 2, tentative annotation by comparison of structural formula and MS/MS fragmentation pattern with data from databases or literature. 3, no data available in literature or databases; compound tentatively assigned on the basis of the MS/MS fragmentation pattern (theoretical interpretation and/or comparison to related compounds); 4, unknown feature of interest; [f] structure additionally verified by Lipid Data analyzer (LDA)[3],[4]; Phospholipid and sphingolipid nomenclature, see [5]

| Nr[a] | Monoiso-topic mass | RT[b] | *m/z*[c] | *m/z* (adduct)[d] | MS/MS[e] | Molecular formula | Δ (ppm) | IL8 priority | NFkB priority | NO priority | extract with high abundance (> average+2 S.D. in Xvar plot) | Annotation [iD level] | Reference |
|---|---|---|---|---|---|---|---|---|---|---|---|---|---|
| **1** | **180.0423** | **10.71** | **179.0333** | | **179.0333 (25)**<br>**135.0433 (100)** | **C9H8O4** | **-3.49** | | **8** | | **23_1, 23_2** | **caffeic acid [2]** | **MZCloud [No 337]** |
| | 676.20000 | 11.81 | 675.1924 | | 675.1918 (15)<br>327.0873 (10)<br>191.0550 (100)<br>173.0443 (10) | C32H36O16 | 0.09 | | | 19 | 18_1, 18_2 | unknown quinic acid derivative [4] | |
| **2** | **338.1002** | **12.63** | **337.0924** | | **173.0441 (100)**<br>**163.0386 (25)**<br>**155.0334 (10)**<br>**137.0227 (10)**<br>**119.0486 (15)**<br>**93.0328 (15)** | **C16H18O8** | **1.74** | | | **12** | **7_1, 7_2; 9_1, 9_2; 18_1, 18_2** | **coumaroylquinic acid [2]** | **literature** [6] |
| **3** | **338.1002** | **13.42** | **337.0925** | | **191.0549 (100)**<br>**173.0442 (15)**<br>**163.0387 (15)**<br>**93.0329 (20)**<br>**87.0070 (10)** | **C16H18O8** | **2.19** | | | **20** | **9_1, 9_2; 18_1, 18_2** | **coumaroylquinic acid [2]** | **literature** [6] |

| | | | | | | | | | | | | | |
|---|---|---|---|---|---|---|---|---|---|---|---|---|---|
| **4** | **404.1319** | **15.62** | **403.1241** | | **371.0993 (10)<br>165.0543 (25)<br>139.0023 (15)<br>123.0436 (15)<br>121.0279 (100)<br>113.0229 (15)<br>101.0228 (45)<br>95.0486 (30)<br>89.0227 (45)<br>71.0122 (40)<br>69.0329 (25)<br>59.0122 (50)** | **C17H24O11** | **1.54** | **9** | | | **35_1, 35_2; 36_1, 36_3** | **Secoxyloganin** [1] | **authentic reference** |
| **5** | **610.1534** | **18.27** | **609.1454** | | **609.1456 (25)<br>447.0958 (5)<br>285.0401 (100)** | **C27H30O16** | **0.72** | | 30 | | **11_1, 11_2; 15_1, 15_2** | **luteolin-dihexoside** [2] | **literature** [7]<br>**MS$^n$ experiments** |
| **6** | **580.1428** | **19.99** | **579.1335** | | **579.1339 (25)<br>447.0884 (1)** | **C26H28O15** | **1.66** | 10 | **13** | | **23_1, 23_2; 30_1, 30_2** | **luteolin hexoside pentoside** [2] | **literature** [7]<br>**MS$^n$ experiments** |
| **7** | **448.101** | **20.91** | **447.0918** | | **447.0920 (40)<br>285.0396 (100)<br>284.0318 (25)** | **C21H20O11** | **-0.93** | | 27 | | **23_1, 23_2** | **luteolin-7-glucoside** [1] | **authentic reference** |
| **8** | **594.1585** | **21.59** | **593.1501** | | **593.1511 (15)<br>447.0928 (35)<br>431.0975 (35)<br>430.0897 (75)<br>285.0400 (100)<br>284.0323 (10)<br>283.0244 (90)** | **C27H30O15** | **-0.01** | | 15 | **2** | **2_1, 2_2; 18_1, 18_2** | **kaempferol-3-hexoside-7-deoxyhexoside** [2] | **literature** [8], [9]<br>**MS$^n$ experiments** |
| **9** | **478.1111** | **21.99** | **477.1036** | | **477.1035 (50)<br>462.0803 (100)<br>315.0510 (75)<br>300.0273 (50)<br>299.0197 (70)<br>271.0238 (10)** | **C22H22O12** | **1.74** | | | **26** | **5_1, 5_2** | **isorhamnetin-7-O-hexoside** [2] | **literature** [10] |
| **10** | **594.1585** | **22.00** | **593.1499** | | **593.1501 (100)<br>447.0918 (10)<br>285.0398 (85)<br>284.0320 (50)** | **C27H30O15** | **-0.33** | 25 | | | **25_1, 25_2** | **lonicerin (luteolin hexoside deoxyhexoside) isomer** [2] | **literature** [11], [12]<br>**MS$^n$ experiments** |

| | | | | | | | | | | | | | |
|---|---|---|---|---|---|---|---|---|---|---|---|---|---|
| **11** | **516.1268** | **22.19** | **515.1885** | | **353.0873 (45)<br>191.0549 (100)<br>179.0337 (80)<br>173.0442 (25)<br>161.0230 (20)<br>135.0435 (20)** | **C25H24O12** | **0.13** | 24 | | | **15_1, 30_2, 33_1** | **dicaffeoylquinic acid ²** | **literature** [13] |
| | 758.2633 | 23.38 | 757.2536 | | 689.3260 (5),<br>525.1597 (55)<br>493.1704 (15)<br>293.0659 (10)<br>261.0389 (15)<br>233.0450 (10)<br>181.0489 (15)<br>119.0330 (35)<br>113.0225<br>(40)101.0226<br>(100) 89.0226<br>(100) 85.0279<br>(10) 71.0210<br>(80) 59.0121<br>(100) | C34H46O19 | -1.79 | 33 | | | 35_1 | UNKNOWN ⁴ | |
| **12** | **516.1268** | **24.18** | **515.1176** | | **353.0867 (25)<br>191.0547 (30)<br>179.034 (75)<br>173.0439 (100)<br>135.0434 (15)** | **C25H24O12** | **-1.53** | | **11** | | **32_1, 32_2** | **dicaffeoylquinic acid ²** | **literature** [13] |
| **13** | **448.1006** | **25.16** | **447.0924** | | **447.0926 (35)<br>285.0400 (25)<br>284.0322 (100)<br>255.0292 (30)<br>227.0340 (15)** | **C21H20O11** | **0.56** | | **2** | | **19_1, 19_2** | **kaempferol-3-hexoside (astragalin isomer) ²** | **literature**[14], [12]<br>**MSⁿ experiments** |
| **14** | **478.1111** | **25.50** | **477.1035** | | **477.1035 (50)<br>314.0431 (100)<br>299.0199 (10)<br>285.0403 (15)<br>271.0247 (20)<br>257.0454 (5)<br>243.0294 (15)** | **C22H22O12** | **1.61** | | | **30** | **5_1, 5_2** | **isorhamnetin-3-O-hexoside ²** | **literature** [10], [15], [16] |

| | | | | | | | | | | | | | |
|---|---|---|---|---|---|---|---|---|---|---|---|---|---|
| 15 | 484.1369 | 26.69 | 483.1294 | | 337.0927 (5)<br>319.0820 (10)<br>191.0552 (20)<br>173.0443 (25)<br>163.0388 (100)<br>145.0281 (5)<br>119.0487 (25) | C25H24O10 | 1.77 | | | 22 | 3_1, 3_2; 5_1, 5_2 | 3,5-di-O-p-coumaroylquinic acid [2] | literature [17] |
| 16 | 610.1323 | 28.94 | 609.1244 | | 609.1243 (30)<br>447.0934 (5)<br>323.0766 (5)<br>285.0401 (100)<br>179.0338 (5)<br>161.0230 (15) | C30H26O14 | 0.83 | | | 29 | 9_1, 9_2 | luteolin-O-caffeoyl-O-hexoside [3] | Fragmentation and MS$^n$ experiments |
| 17 | 484.1369 | 29.84 | 483.1290 | | 337.0926 (15)<br>191.0550 (5)<br>137.0230 (5)<br>119.0487 (5)<br>173.0442 (100)<br>163.0387 (25) | C25H24O10 | 0.83 | | | 16 | 7_1, 7_2; 18_1, 18_2 | 4,5-di-O-p-coumaroylquinic acid [2] | literature [17] |
| 18 | 286.0477 | 30.06 | 285.040 | | 285.0400 (100)<br>151.0021 (5)<br>133.0279 (5) | C15H10O6 | 2.26 | 28 | 3 | | 15_1, 15_2; 25_1, 25_2 | luteolin [1] | authentic reference |
| 19 | 316.0583 | 30.89 | 315.0506 | | 315.0505 (25)<br>300.0269 (100)<br>272.0320(5)<br>151.0021 (10) | C16H12O7 | 2.13 | 39 | | 21 | 14_1, 14_2 | isorhamnetin [2] | MoNA [spectrum FiehnHILIC001279] |
| | 536.1894 | 31.15 | 535.1810 | | 535.1799 (5)<br>373.1280 (5)<br>341.1019 (10)<br>313.1075 (10)<br>209.0809 (10)<br>163.0386 (100)<br>119.0485 (30)<br>101.0227 (25) | C26H232O12 | 0.05 | | 34 | | 19_1, 19_2 | UNKNOWN [4] | |
| 20 | 594.1373 | 31.73 | 593.1294 | | 593.1295 (35)<br>447.0928 (5)<br>285.0400 (100)<br>163.0383 (3)<br>145.0280 (10) | C30H26O13 | 0.71 | | | 27 | 9_1, 9_2 | luteolin-O-coumaroyl-O hexoside [3] | Fragmentation and MS$^n$ experiments |

| | | | | | | | | | | | | | |
|---|---|---|---|---|---|---|---|---|---|---|---|---|---|
| **21** | **270.0528** | **32.50** | **269.0452** | | **269.0452 (100)<br>225.0548 (10)<br>151.0022 (10)<br>149.0225 (5)<br>117.0328 (5)** | **$C_{15}H_{10}O_5$** | **2.68** | | **25** | | **15_1, 15_2** | **apigenin** [1] | **authentic reference** |
| **22** | ***358.105*** | ***32.64*** | ***357.098*** | | ***357.0980 (45)<br>342.0743 (60)<br>327.0509 (100)<br>313.0718 (10)<br>299.0561 (20)*** | ***$C_{19}H_{18}O_7$*** | ***3.03*** | ***8*** | | ***7*** | ***5_1, 5_2; 14_1, 14_2*** | ***7-hydroxy-5,3´,4´,5´-tetramethoxyflavone*** [1] | ***isolated & identified*** |
| **23** | **300.0634** | **32.79** | **299.0555** | | **299.0555 (30)<br>284.0320 (100)<br>256.0370 (5)** | **$C_{16}H_{12}O_6$** | **1.56** | | **26** | | **25_1, 25_2** | **diosmetin/ chrysoeriol** [2] | **MoNA [spectrum Spectrum VF-NPL-QEHF014481/MetaboBASE0344 ]** |
| | 584.0955 | 32.89 | 583.0881 | | 583.0880 (50)<br>568.0641 (50)<br>539.0956 (5)<br>497.0888 (10)<br>421.0568 (15)<br>417.0614 (20)<br>406.0330 (30)<br>375.0509 (100)<br>333.0406 (5)<br>257.0074 (10)<br>207.0287 (15)<br>192.0052(20)<br>163.0386 (5) | $C_{31}H_{20}O_{12}$ | 0.82 | | | 23 | 5_1, 5_2; 14_1, 14_2 | UNKNOWN [4] | |
| **24** | **328.225** | **33.76** | **327.2169** | | **327.2168 (100)<br>291.1952 (15)<br>239.1278 (10)<br>229.1434 (45)<br>221.1170 (15)<br>211.1327 (60)<br>183.1377 (10)<br>171.1010 (70)<br>137.0955 (10)<br>97.0641 (10)<br>85.0277 (25)** | **$C_{18}H_{32}O_5$** | **-0.70** | | **23** | | **31_1, 31_2** | **trihydroxyocta-decadienoic acid isomer I** [2] | **literature** [18] |

| | | | | | | | | | | | | | |
|---|---|---|---|---|---|---|---|---|---|---|---|---|---|
| **25** | **328.2250** | **33.98** | **327.2169** | | **327.2169 (100)<br>291.1957 (15)<br>239.1279 (10)<br>229.1435 (55)<br>221.1171 (15)<br>211.1327 (80)<br>183.1377 (10)<br>171.1010 (25)<br>97.0641 (10)<br>85.0277 (10)** | **C18H32O5** | **-0.70** | **19** | **1** | | **29_1, 29_2; 31_1, 31_2** | **trihydroxyoctadecadienoic acid isomer II** [2] | **literature** [18] |
| **26** | ***538.0900*** | ***35.07*** | ***537.0825*** | | ***537.0823 (35)<br>443.0406 (15)<br>417.0614 (30)<br>399.0504(15)<br>375.0506 (100)*** | ***C30H18O10*** | ***1.56*** | | **17** | **1** | **5_1, 5_2; 14_1, 14_2** | ***cupressuflavone*** [0] | ***isolated & identified*** |
| **27** | ***568.1006*** | ***35.29*** | ***567.0928*** | | ***567.0930 (100)<br>473.0503 (15)<br>447.0719 (25)<br>403.0407 (15)<br>417.0623 (20)<br>405.0614 (70)<br>390.0381 (35)<br>375.0508 (100)*** | ***C31H20O11*** | ***1.13*** | | **24** | **8** | **5_1, 5_2; 14_1, 14_2** | ***3´-methoxycupressuflavone*** [0] | ***isolated & identified*** |
| **28** | **330.2406** | **35.43** | **329.2325** | | **329.2325 (100)<br>311.2224 (5)<br>293.2117 (5)<br>229.1435 (45)<br>211.1327 (65)<br>193.1217 (5)<br>183.1376 (10)<br>171.1011 (30)<br>139.1112 (10)<br>178.1110 (5)<br>99.0798 (10)** | **C18H34O5** | **-0.88** | | **32** | | **29_1, 29_2** | **trihydroxyocta-decenoic acid** [2] | **literature** [18] |
| **29** | ***598.1111*** | ***35.51*** | ***597.1036*** | | ***597.1038 (80)<br>473.0500 (10)<br>447.0724 (25)<br>405.0614 (100)<br>390.0381 (50)*** | ***C32H22O12*** | ***1.49*** | 36 | **20** | **10** | **5_1, 5_2; 14_1, 14_2** | ***3´, 3´´-dimethoxycupressuflavone*** [0] | ***isolated & identified*** |

| | | | | | | | | | | | | | |
|---|---|---|---|---|---|---|---|---|---|---|---|---|---|
| | 306.1831 | 35.91 | 305.1751 | | 305.1752 (25)<br>249.1487 (75)<br>205.1586 (15)<br>135.0798 (100)<br>125.0955 (20)<br>1234.0795 (10)<br>121.0640 (5)<br>97.0640<br>79.0534 (5)<br>71.0485 (19 | C18H26O4 | -0.67 | | 33 | | 20_1, 20_2; 31_1, 31_2 | UNKNOWN [4] | |
| | | 36.912 | 1041.4863 $[M+CF_3COO]^-$ | | 671.3763 (15)<br>603.3882 (20)<br>112.9838 (100) | C49H76O20F3 | -1.63 | 29 | | | 1_1, 2_2; 30_1, 30_2 | TFA adduct of akebiasaponin D (30) [3] | |
| **30** | **928.5032** | **36.92** | **927.4937** | | **603.3904 (100)**<br>**323.1013 (2)** | **C47H76O18** | **1.76** | **2** | | | **1_1, 1_2; 30_1, 30_2** | **Akebia saponin D** [2] | **literature** [19], [20] |
| **31** | **444.2049** | **36.99** | **443.1969** | | **383.1758 (15)**<br>**353.1624 (3)**<br>**292.1215 (5)**<br>**262.1239 (10)**<br>**248.0948 (10)**<br>**222.0913 (5)**<br>**201.0658 (5)**<br>**120.0431 (15)**<br>**59.0122 (100)** | **C27H28O4N2** | **-0.48** | | **9** | | **21_1, 21_2; 25_1, 25_2** | **aurantiamide acetate** [2] | **literature** [21] |
| | 1076.1800 | 37.227 | 1075.1698 $[2M-H]^-$ | | 537.0812 (100)<br>443.0395 (20)<br>417.0606 (25)<br>375.0500 (75) | C60H36O20 | -2.2 | | 28 | | 21_2; 31_1 | amentoflavone dimer | |
| **32** | **538.0900** | **37.24** | **537.0817** | | **537.0815 (50)**<br>**443.0398 (25)**<br>**417.0609 (35)**<br>**399.0500 (20)**<br>**375.0504 (100)**<br>**331.0608 (15)**<br>**203.0343 (3)**<br>**159.0436 (5)**<br>**117.0328 (5)** | **C30H18O10** | **-0.93** | | **4** | **31** | **(20_1), 20_2** | **amentoflavone** [1] | **authentic reference** |

| | | | | | | | | | | | | | |
|---|---|---|---|---|---|---|---|---|---|---|---|---|---|
| **33** | **552.1056** | **40.28** | **551.0975** | | **551.0976 (30)<br>375.0505 (100)<br>331.0607 (10)<br>307.0608 (3)<br>173.0597 (5)** | **C31H20O10** | **-0.53** | | 18 | **25** | **9_1, 9_2; 26_1, 26_2** | **podocarpusflavone A** [1] | **authentic reference** |
| **34** | **538.0900** | **40.96** | **537.0808** | | **537.0811 (100)<br>493.0918 (5)<br>469.0914 (10)<br>385.0700 (5)<br>285.0396 (3)<br>284.0320 (5)<br>269.0450 (3)<br>151.0018 (5)** | **C30H18O10** | **-2.54** | | **12** | | **32_1, (32_2)** | **hinokiflavone** [2]**/ochnaflavone** [2] | **literature** [22] |
| **35** | **538.0900** | **41.43** | **537.082** | | **537.0817 (100)<br>493.0919 (5)<br>469.0921 (10)<br>385.0711 (5)<br>285.0396 (2)<br>284.0324 (5)<br>269.0453 (3)<br>151.0020 (5)** | **C30H18O10** | **-0.38** | | **29** | | **19_1, 19_2; 29_1, 29_2** | **hinokiflavone** [2]**/ochnaflavone**[2] | **literature** [22] |
| **36** | **294.4298** | **41.93** | **293.2117** | | **293.2116 (75)<br>275.2011 (100)<br>235.1694 (60)<br>231.2119 (10)<br>211.1330 (10)<br>195.1378 (7)<br>183.1377 (35)<br>177.1633 85)<br>171.1011 (35)<br>121.1006 (25)** | **C18H30O3** | **2.8** | | **5** | | **25_1, 25_2; 31_1, 31_2** | **oxo-octadecadienoic acid** [2] | **literature** [23] |
| **37** | **766.4503** | **41.95** | **765.4404** | | **765.4413 (70)<br>603.3382 (100)<br>585.3773 (25)<br>525.3582 (5)<br>471.3465 (50)<br>423.3230 (10)<br>113.0227 (10)<br>101.0226 (20)<br>59.0121 (20)** | **C41H66O13** | **-2.81** | **34** | | | **1_1, 1_2; 30_1, 30_2** | **Akebia saponin C** [2] | **literature** [24] |

| | | | | | | | | | | | | | |
|---|---|---|---|---|---|---|---|---|---|---|---|---|---|
| 38 | 604.3975 | 42.77 | 603.3885 | | 603.3885 (100)<br>427.2431 (1)<br>391.8430 (1) | C35H56O8 | -1.99 | 16 | | | 1_1, 1_2; 30_1, 30_2 | Akebia saponin PA [2] | literature [24] |
| 39 | 676.3670 | 43.50 | 721.3638 [M+HCOO]- | | 415.1450 (15)<br>397.1338 (20)<br>277.2164 (100)<br>253.0917 )5)<br>235.0813 (15)<br>179.0541 (5)<br>161.0436 (5)<br>143.0331 (5)<br>125.0225 (10)<br>119.0330 (10)<br>101.0226 (30)<br>89.0226 (25)<br>71.0121 (20)<br>59.0121 (30) | C33H56O14 | -0.72 | 26 | | | 30_2; 36_1, 36_2 | gingerglycolipid A isomer [2] | literature [25] |
| 40 | 517.3168 | 44.07 | 562.3136 [M+HCOO]- | 562.314 [M+HCOO]-; 502.29 [M-CH3]- | 502.2927 (15)<br>277.3264 (100)<br>224.0685 (15) | C26H48NO7P | -1.59 | 23 | | | 25_1; 29_1; 30_2 | LPC 18:3 [2,f] | MoNA [spectrum LipidBlast454264]; LDA[3] (supplementary figure S7) |
| | 372.3240 | 46.83 | 371.3154 | | 371.3154 (40)<br>353.3055 (5)<br>311.2944 (20)<br>183.1738 (5)<br>59.0121 (100) | C22H44O4 | -1.82 | 3 | | | 35_1 | UNKNOWN [4] | |
| 41 | 602.4758 | 47.19 | 601.4681 | | 601.4683 (40)<br>541.4470 (5)<br>499.4365 (20)<br>115.0747 (5)<br>59.0123 | C34H66O8 | 0.33 | 5 | 14 | 4 | 5_1, 5_2; 14_1, 14_2 | trihydroxy-monoacetoxy-dotriacontanoic acid I [3] | n.a. |
| 42 | 602.4758 | 47.55 | 601.4682 | | 601.4684 (60)<br>583.4583 (10)<br>541.4466 (10)<br>499.4361 (25)<br>427.3806 (3)<br>297.2806 (3)<br>241.8599 (3) | C34H66O8 | 0.43 | 15 | | 13 | 5_1, 5_2; 14_1, 14_2 | trihydroxy-monoacetoxy-dotriacontanoic acid II [3] | n.a. |

| | | | | | | | | | | | | | |
|---|---|---|---|---|---|---|---|---|---|---|---|---|---|
| | | | | | **115.0748 (5)<br>59.0123 (100)** | | | | | | | | |
| **43** | ***644.4860*** | ***47.96*** | ***643.4689*** | | ***643.4792 (25)<br>601.4709 (3)<br>583.4568 (10)<br>523.4394 (3)<br>499.4388 (10)<br>299.4382 (2)<br>59.0123 (100)*** | ***C36H68O9*** | ***0.58*** | 6 | **22** | **5** | **5_1, 5_2; 14_1, 14_2** | ***trihydroxy-diacetoxy-dotriacontanoic acid I*** [2] | ***isolated & partly identified*** |
| | 400.3553 | 48.09 | 399.3467 | | 399.3467 (45)<br>381.3349 (5)<br>339.2357 (20)<br>59.0121 (100) | C24H48O4 | -0.44 | 22 | | | 35_1; 36_1, 36_2 | UNKNOWN [4] | |
| **44** | ***644.4860*** | ***48.24*** | ***643.4788*** | | ***643.4790 (35)<br>625.4689 (5)<br>583.4576 (15)<br>523.4355 (2)<br>499.4371 (10)<br>283.2639 (2)<br>115.0747 (2)<br>59.0123 (100)*** | ***C36H68O9*** | ***0.39*** | 17 | **31** | **9** | **5_1, 5_2; 14_1, 14_2** | ***trihydroxy-diacetoxy-dotriacontanoic acid II*** [2] | ***isolated & partly identified*** |
| **45** | **482.2645** | **48.42** | **481.2559** | | **481.2551 (35)<br>253.2163 (35)<br>245.0422 (100)<br>227.0315 (15)<br>152.9941 (100)<br>78.9571 (10)** | **C22H42O9P** | **-0.37** | **35** | | | **25_1, 25_2; 30_1, 30_2** | **LPG 16:1** [2] | **literature** [26] |
| **46** | ***644.4860*** | ***48.45*** | ***643.4786*** | | ***643.4788 (30)<br>601.4670 (5)<br>583.4584 (10)<br>499.4364 (10)<br>481.4247 (3)<br>59.0122 (100)*** | ***C36H68O9*** | ***0.11*** | 11 | **6** | **3** | **5_1, 5_2; 14_1, 14_2** | ***trihydroxy-diacetoxy-dotriacontanoic acid III*** [2] | ***isolated & partly identified*** |
| **47** | **630.5071** | **48.60** | **629.4995** | | **629.4994 (45)<br>611.4891 (3)<br>569.4760 (10)<br>527.4674 (20)<br>115.0748 (5)<br>59.0123 (100)** | **C36H70O8** | **1.20** | **32** | | **14** | **5_1, 5_2; 14_1, 14_2** | **tetrahydroxy-monoacetoxy-tetratriacontanoic acid I** [3] | **n.a.** |

| | | | | | | | | | | | | | |
|---|---|---|---|---|---|---|---|---|---|---|---|---|---|
| 48 | 556.2906 | 48.91 | 556.2917 | | 555.2827 (100)<br>299.0062 (25)<br>164.0847 (10)<br>94.9791 (5)<br>80.9634 (15) | C25H48O11S | -0.72 | | 21 | | 25_1; 30_2 | palmitoyl-sulfoquinovosyl-glycerol [2] | literature [27] |
| 49 | 630.5071 | 48.96 | 629.4994 | | 629.4995 (50)<br>611.4905 (5)<br>569.4794 (10)<br>527.4677 (20)<br>509.4564 (3)<br>325.3122 (3)<br>253.2528 (3)<br>187.1324 (3)<br>115.0750 (5)<br>59.0123 (100) | C36H70O8 | 1.10 | 13 | | 11 | 5_1, 5_2; 14_1, 14_2 | tetrahydroxy-monoacetoxy-tetratriacontanoic acid II [3] | n.a. |
| 50 | 686.4969 | 49.16 | 685.4894 | | 685.4892 (20)<br>643.4768 (5)<br>625.4685 (10)<br>583.4575 (3)<br>565.4472 (5)<br>499.4341 (3)<br>59.0122 (100) | C38H70O10 | 1.34 | 40 | | 18 | 5_1, 5_2 | dihydroxy-triacetoxy-dotriacontanoic acid [3] | n.a. |
| 51 | 672.5176 | 49.24 | 671.5097 | | 671.5100 (35)<br>653.5009 (3)<br>611.4897 (10)<br>551.4673 (3)<br>527.4663 (5)<br>59.0122 (100) | C38H72O9 | 0.57 | | | 17 | 3_1, 3_2; 5_1, 5_2; 14_1, 14_2 | trihydroxy-diacetoxy-tetratriacontanoic acid I [3] | n.a. |
| 52 | 592.2686 | 49.32 | 591.2602 | | 515.2440 (100)<br>500.220(25)<br>497.2335 (7)<br>487.2531 (7)<br>443.2235 (3) | C35H36N4O5 | -0.01 | | 7 | | 25_1, 25_2; 31_1, 31_2 | pheophorbide A [1] | authentic reference |
| 53 | 672.5176 | 49.52 | 671.5103 | | 671.5100 (40)<br>653.5009 (3)<br>611.4897 (10)<br>551.4673 (3)<br>527.678 (5)<br>311.2943 (3)<br>59.0122 (100) | C38H72O9 | 1.47 | 31 | | 15 | 3_1, 3_2; 5_1, 5_2; 14_1, 14_2 | trihydroxy-diacetoxy-tetratriacontanoic acid II [3] | n.a. |

| | | | | | | | | | | | | | |
|---|---|---|---|---|---|---|---|---|---|---|---|---|---|
| ***54*** | ***672.5176*** | ***49.75*** | ***671.5101*** | | ***671.5104 (25)<br>629.5004 (3)<br>611.4898 (10)<br>527.4669 (10)<br>59.0123 (100)*** | ***$C_{38}H_{72}O_9$*** | ***1.3*** | | ***10*** | ***6*** | ***3_1, 3_2; 5_1, 5_2; 14_1, 14_2*** | ***trihydroxy-diacetoxy-tetratriacontanoic acid III*** [2] | ***isolated and partly identified*** |
| **55** | **484.2801** | **49.77** | **483.272** | | **483.2719 (15)<br>255.2322 (100)<br>227.0315 (5)<br>152.9942 (25)<br>78.9572 (5)** | **$C_{22}H_{44}O_9P$** | **0.46** | **7** | | | **25_1, 25_2; 29_2; 36_1, 36_2** | **LPG 16:0** [2] | **literature** [26] |
| **56** | **714.5282** | **50.34** | **713.5208** | | **713.5201 (20)<br>671.5075 (5)<br>653.5010 (10)<br>593.4786 (5)<br>527.4651 (3)<br>59.0122 (100)** | **$C_{40}H_{74}O_{10}$** | **1.38** | | | **24** | **3_1, 3_2; 5_1, 5_2; 14_1, 14_2** | **dihydroxy-triacetoxy-tetratriacontanoic acid** [3] | **n.a.** |
| | 700.5489 | 50.86 | 699.5407 | | 699.5401 (30)<br>639.5221 (10)<br>555.4976 (10)<br>59.0122 (100) | $C_{40}H_{76}O_9$ | 0.21 | | | 28 | 3_1, 3_2; 5_1, 5_2; 14_1, 14_2 | UNKNOWN [4] | |
| **57** | **713.5435** | **51.99** | **712.5349** | | **712.5335 (15)<br>550.4824 (35)<br>532.4717 (45)<br>306.2426 (10)<br>295.2581 (25)<br>278.2468 (25)<br>253.2148 (15)<br>225.2213 (35)<br>119.0334 (25)<br>113.0226 (35)<br>101.0227 (65)<br>89.0226 (85)<br>71.0120 (70)<br>59.0121 (100)** | **$C_{40}H_{75}O_9N$** | **-2.03** | **21** | | | **1_1, 1_2** | **soyacerebroside (HexCer 18:2;O2/16:0;O)** [2] | **literature** [23], [28] |
| **58** | **384.3603** | **52.47** | **383.3535** | | **383.3523 (100)<br>365.3411 (3)<br>337.3470 (75)** | **$C_{24}H_{48}O_3$** | **1.4** | | **19** | | **24_1, 24_2** | **hydroxytetracosanoic acid** [2] | **literature** [29] |
| | | 53.94 | 473.2813 | | 473.2811 (100)<br>457.2520 (3)<br>205.1589 (3) | ? | | 27 | | | 36_1, 36_2 | UNKNOWN [4] | |

| | | | | | | | | | | | | | |
|---|---|---|---|---|---|---|---|---|---|---|---|---|---|
| **59** | **669.5907** | **54.33** | **668.5819** | **668.580 [M-H]$^-$; 714.590 [M+HCOO]$^-$** | **668.5800 (20)<br>372.2744 (100)<br>354.2632 (10)<br>336.2557 (10)<br>306.2430 (15)<br>265.2164 (35)<br>253.2159 (10)<br>130.0492 (85)<br>118.0491 (100)<br>100.0386 (30)<br>75.0070 (70)** | **C40H79O6N** | **-0.77** | **39** | | | **33_1, 33_2; 36_1, 36_2** | **Cer 18:1; O3/22:0;O2** [2,f] | **literature** [30], **LDA**[4] **(supplementary figure S8)** |
| | | 55.43 | 766.5795 [M+CF3COO]- | | 112.9837 (100)<br>68.9940 (50) | C42H79O7NF3 | -1.1 | 18 | | | 30_1; 34_1; 36_1, 36_2 | TFA adduct of 60 [3] | |
| **60** | **653.5958** | **55.44** | **652.5867** | **652.590 [M-H]$^-$<br>688.560 [M+Cl]$^-$** | **652.5869 (90)<br>634.5752 (5)<br>410.3625 (35)<br>398.3631 (50)<br>380.3522 (15)<br>355.3208 (90)<br>337.3098 (20)<br>309.3154 (100)<br>282.2429 (10)<br>265.2161 (25)<br>253.2167 (35)<br>223.2051 (10)<br>88.0386 (10)** | **C40H79O5N** | **-1.11** | **14** | | | **1_1, 1_2; 34_1** | **Cer 18:1;O3/22:0; O** [2,f] | **literature** [30], **LDA**[4] **(supplementary figure S9)** |
| **61** | **697.6220** | **55.60** | **696.6129** | **696.610 [M-H]$^-$; 742.619 [M+HCOO]$^-$;** | **696.6117 (20)<br>372.2746 (100)<br>354.2651 (10)<br>336.2528 (15)<br>306.2430 (20)<br>265.2165 (35)<br>253.21654 (15)<br>130.0492 (85)<br>118.0492 (100)100.0386 (30) 75.0099 (80)** | **C42H83O6N** | **-1.07** | **4** | **16** | | **33_1, 33_2; 36_1, 36_2** | **Cer18:1;O3/24:0; O2** [2, f] | **literature** [30], **LDA**[4] **(supplementary figure S10)** |

| | | | | | | | | | | | | | |
|---|---|---|---|---|---|---|---|---|---|---|---|---|---|
| | **810.6071** | **55.60** | **810.6058 [M+CF3COO]-** | | **112.9837 (100)<br>68.9940 (50)** | **C44H83O8NF3** | **-0.94** | **12** | **35** | | **25_1, 25_2; 33_1, 33_2; 36_1, 36_2** | **TFA adduct of 61** [3] | |
| **62** | **667.6115** | **56.15** | **780.5953 [M+CF3COO]-** | **666.600 [M-H]$^-$; 712.610 [M+HCOO]-; 702.580 [M+Cl]$^-$** | **666.6024 (100)<br>648.5941 (10)<br>424.3790 (40)<br>412.3785 (50)<br>394.3656 (15)<br>369.3362 (95)<br>351.3263 (20)<br>323.3309 (100)<br>282.2424 (15)<br>265.2163 (25)<br>253.2163 (35)<br>223.2056 (10)<br>88.0387 (15)** | **C41H81O5N** | **-1.02** | **30** | | | **25_1, 25_2; 30_2; 36_1, 36_2** | **Cer 18:1;O3/23:0;O** [2,f] | **literature** [30], **LDA**[4] **(supplementary figure S11)** |
| **63** | **681.6271** | **56.95** | **680.6178** | **680.620 [M-H]$^-$; 726.624 [M+HCOO]$^-$; 716.598 [M+Cl]$^-$** | **680.6177 (85)<br>438.3945 (35)<br>426.3944 (45)<br>383.3522 (90)<br>365.3412 (20)<br>337.3467 (100)<br>282.2436 (10)<br>265.2167 (25)<br>253.2163 (35)<br>88.0387 (10)** | **C42H83O5N** | **-1.4** | **1** | | | **1_1, 1_2** | **Cer18:1;O3/24:0;O** [2,f] | **literature** [30], **LDA**[4] **(supplementary figure S12)** |
| **64** | **683.6428** | **57.83** | **796.6273 [M+CF3COO]$^-$** | **682.630 [M-H]$^-$; 728.640 [M+HCOO]$^-$; 718.610 [M+Cl]$^-$** | **682.6344 (100)<br>438.3947 (25)<br>426.3950 (45)<br>383.3524 (80)<br>365.3428 (15)<br>337.3471 (100)<br>284.2592 (10)<br>267.2326 (25)<br>255.2320 (30)<br>225.2214 (5)<br>88.0387 (10)** | **C42H85O5N** | **-0.36** | **20** | | | **25_1, 25_2; 36_1, 36_2** | **Cer18:0;O3/24:0;O** [2,f] | **literature** [30], **LDA** [4] **(supplementary figure S13)** |

| | | | | | | | | | | | | | |
|---|---|---|---|---|---|---|---|---|---|---|---|---|---|
| **65** | **709.6584** | **58.85** | **822.6422 [M+CF3COO]-** | **708.650 [M-H]$^-$; 754.650 [M+HCOO]$^-$; 744.627 [M+Cl]$^-$** | **708.6490 (95)<br>690.6365 (5)<br>466.4243 (35)<br>454.4252 (55)<br>436.4153 (10)<br>411.3831 (90)<br>393.3730 (15)<br>365.3776 (100)<br>282.2426 (10)<br>265.2165 (25)<br>253.2161 (30)<br>232.9139 (10)<br>174.9546 (10)<br>164.9258 (10)<br>88.0387 (10)** | **C44H87O5N** | **-1.5** | **37** | | | **34_1; 36_1, 36_2; 25_1, 25_2** | **Cer18:1;O3/26:0; O[2,f]** | **literature** [30], **LDA**[4] **(supplementary figure S14)** |

**References:**


1. Sumner, L.W.; Amberg, A.; Barrett, D.; Beale, M.H.; Beger, R.; Daykin, C.A.; Fan, T.W.M.; Fiehn, O.; Goodacre, R.; Griffin, J.L.; et al. Proposed minimum reporting standards for chemical analysis: Chemical Analysis Working Group (CAWG) Metabolomics Standards Initiative (MSI). *Metabolomics* **2007**, *3*, 211–221, doi:10.1007/s11306-007-0082-2.

2. Blaženović, I.; Kind, T.; Ji, J.; Fiehn, O. Software tools and approaches for compound identification of LC-MS/MS data in metabolomics. *Metabolites* **2018**, *8*, doi:10.3390/metabo8020031.

3. Hartler, J.; Triebl, A.; Ziegl, A.; Trötzmüller, M.; Rechberger, G.N.; Zeleznik, O.A.; Zierler, K.A.; Torta, F.; Cazenave-Gassiot, A.; Wenk, M.R.; et al. Deciphering lipid structures based on platform-independent decision rules. *Nat. Methods* **2017**, *14*, 1171–1174, doi:10.1038/nmeth.4470.

4. Hartler, J.; Armando, A.M.; Trötzmüller, M.; Dennis, E.A.; Köfeler, H.C.; Quehenberger, O. Automated Annotation of Sphingolipids including Accurate Identification of Hydroxylation Sites Using MS$^n$ Data. *Anal. Chem.* **2020**, *92*, 14054–14062, doi:10.1021/acs.analchem.0c03016.

5. Liebisch, G.; Fahy, E.; Aoki, J.; Dennis, E.A.; Durand, T.; Ejsing, C.S.; Fedorova, M.; Feussner, I.; Griffiths, W.J.; Köfeler, H.; et al. Update on LIPID MAPS classification, nomenclature, and shorthand notation for MS-derived lipid structures. *J. Lipid Res.* **2020**, *61*, 1539–1555, doi:10.1194/jlr.S120001025.

6. Zhang, S.H.; Hu, X.; Shi, S.Y.; Huang, L.Q.; Chen, W.; Chen, L.; Cai, P. Typical ultraviolet spectra in combination with diagnostic mass fragmentation analysis for the rapid and comprehensive profiling of chlorogenic acids in the buds of Lonicera macranthoides. *Anal. Bioanal. Chem.* **2016**, *408*, 3659–3672, doi:10.1007/s00216-016-9450-y.

7. Jaiswal, R.; Müller, H.; Müller, A.; Karar, M.G.E.; Kuhnert, N. Identification and characterization of chlorogenic acids, chlorogenic acid glycosides and flavonoids from Lonicera henryi L. (Caprifoliaceae) leaves by LC-MSn. *Phytochemistry* **2014**, *108*, 252–263, doi:10.1016/j.phytochem.2014.08.023.

8. Rösch, D.; Krumbein, A.; Mügge, C.; Kroh, L.W. Structural investigations of flavonol glycosides from sea buckthorn (Hippophaë rhamnoides) pomace by NMR spectroscopy and HPLC-ESI-MS n. *J. Agric. Food Chem.* **2004**, *52*, 4039–4046, doi:10.1021/jf0306791.

9. Qin, Y.; Gao, B.; Shi, H.; Cao, J.; Yin, C.; Lu, W.; Yu, L.; Cheng, Z. Journal of Pharmaceutical and Biomedical Analysis Characterization of flavonol mono- , di- , tri- and tetra- O -glycosides by ultra-performance liquid chromatography-electrospray ionization-quadrupole time-of-flight mass spectrometry and its application f. *J. Pharm. Biomed. Anal.* **2017**, *142*, 113–124, doi:10.1016/j.jpba.2017.05.007.

10. Amaral, J.G.; Bauermeister, A.; Pilon, A.C.; Gouvea, D.R.; Sakamoto, H.T.; Gobbo-Neto, L.; Lopes, J.L.C.; Lopes, N.P. Fragmentation pathway and structural characterization of new glycosylated phenolic derivatives from Eremanthus glomerulatus less (Asteraceae) by electrospray ionization tandem mass spectrometry. *J. Mass Spectrom.* **2017**, *52*, 783–787, doi:10.1002/jms.3982.

11. Shengyun, D.; Yuqi, W.; Fei, W.; Jiayu, Z. metabonomics analysis for the rapid detection and mechanistic understanding of sulfur fumigation of Chinese herbal medicines †. **2019**, 31150–31161, doi:10.1039/c9ra05032a.

12. Cai, Z.; Liao, H.; Wang, C.; Chen, J.; Tan, M.; Mei, Y.; Wei, L.; Chen, H.; Yang, R.; Liu, X. A comprehensive study of the aerial parts of Lonicera japonica Thunb. based on metabolite profiling coupled with PLS-DA. *Phytochem. Anal.* **2020**, 1–15, doi:10.1002/pca.2943.

13. Sun, H.; Liu, M.; Lin, Z.; Jiang, H.; Niu, Y.; Wang, H.; Chen, S. Comprehensive identification of 125 multifarious constituents in Shuang-huang-lian powder injection by HPLC-DAD-ESI-IT-TOF-MS. *J. Pharm. Biomed. Anal.* **2015**, *115*, 86–106, doi:10.1016/j.jpba.2015.06.013.

14. Kite, G.C.; Veitch, N.C. Assigning glucose or galactose as the primary glycosidic sugar in 3- O -mono-, di- and triglycosides of kaempferol using negative ion electrospray and serial mass spectrometry. *Rapid Commun. Mass Spectrom.* **2009**, *23*, 3125–3132, doi:10.1002/rcm.4217.

15. Yang, W.Z.; Qiao, X.; Bo, T.; Wang, Q.; Guo, D.A.; Ye, M. Low energy induced homolytic fragmentation of flavonol 3-O-glycosides by negative electrospray ionization tandem mass spectrometry. *Rapid Commun. Mass Spectrom.* **2014**, *28*, 385–395, doi:10.1002/rcm.6794.

16. Seo, O.N.; Kim, G.S.; Park, S.; Lee, J.H.; Kim, Y.H.; Lee, W.S.; Lee, S.J.; Kim, C.Y.; Jin, J.S.; Choi, S.K.; et al. Determination of polyphenol components of Lonicera japonica Thunb. using liquid chromatography-tandem mass spectrometry: Contribution to the overall antioxidant activity. *Food Chem.* **2012**, *134*, 572–577, doi:10.1016/j.foodchem.2012.02.124.

17. Gouvea, D.R.; Meloni, F.; Ribeiro, A. de B.B.; Lopes, J.L.C.; Lopes, N.P. A new HPLC-DAD-MS/MS method for the simultaneous determination of major compounds in the crude extract of Lychnophora salicifolia Mart. (Brazilian arnicão) leaves: Application to chemical variability evaluation. *Anal. Chim. Acta* **2012**, *748*, 28–36, doi:10.1016/j.aca.2012.08.027.

18. Han, B.; Xin, Z.; Ma, S.; Liu, W.; Zhang, B.; Ran, L.; Yi, L.; Ren, D. Comprehensive characterization and identification of antioxidants in Folium Artemisiae Argyi using high-resolution tandem mass spectrometry. *J. Chromatogr. B Anal. Technol. Biomed. Life Sci.* **2017**, *1063*, 84–92,

doi:10.1016/j.jchromb.2017.08.021.

19. Wang, Y.; He, Y.; Dai, Z.; Ma, S. Simultaneous determination of five triterpenoid saponins in different parts of Lonicera macranthoides by RRLC-MS/MS method. *Pharmazie* **2016**, *71*, 306–310, doi:10.1691/ph.2016.6012.

20. Gao, W.; Yang, H.; Qi, L.W.; Liu, E.H.; Ren, M.T.; Yan, Y.T.; Chen, J.; Li, P. Unbiased metabolite profiling by liquid chromatography-quadrupole time-of-flight mass spectrometry and multivariate data analysis for herbal authentication: Classification of seven Lonicera species flower buds. *J. Chromatogr. A* **2012**, *1245*, 109–116, doi:10.1016/j.chroma.2012.05.027.

21. Calderón, A.I.; Hodel, A.; Wolfender, J.L.; Gupta, M.P.; Correa, M.; Hostettmann, K. LC-DAD-MS-based metabolite profiling of three species of Justicia (Acanthaceae). *Nat. Prod. Res.* **2013**, *27*, 1335–1342, doi:10.1080/14786419.2012.738207.

22. Yao, H.; Chen, B.; Zhang, Y.; Ou, H.; Li, Y.; Li, S.; Shi, P.; Lin, X. Analysis of the total biflavonoids extract from Selaginella doederleinii by HPLC-QTOF-MS and its in vitro and in vivo anticancer effects. *Molecules* **2017**, *22*, 325, doi:10.3390/molecules22020325.

23. Geng, P.; Harnly, J.M.; Chen, P. Differentiation of Whole Grain from Refined Wheat (T. aestivum) Flour Using Lipid Profile of Wheat Bran, Germ, and Endosperm with UHPLC-HRAM Mass Spectrometry. *J. Agric. Food Chem.* **2015**, *63*, 6189–6211, doi:10.1021/acs.jafc.5b01599.

24. Lü, S.; Zhao, S.; Zhao, M.; Guo, Y.; Li, G.; Yang, B.; Wang, Q.; Kuang, H. Systematic screening and characterization of prototype constituents and metabolites of triterpenoid saponins of Caulopphyllum robustum Maxim using UPLC-LTQ Orbitrap MS after oral administration in rats. *J. Pharm. Biomed. Anal.* **2019**, *168*, 75–82, doi:10.1016/j.jpba.2019.02.005.

25. Jiménez-Sánchez, C.; Lozano-Sánchez, J.; Rodríguez-Pérez, C.; Segura-Carretero, A.; Fernández-Gutiérrez, A. Comprehensive, untargeted, and qualitative RP-HPLC-ESI-QTOF/MS2 metabolite profiling of green asparagus (Asparagus officinalis). *J. Food Compos. Anal.* **2016**, *46*, 78–87, doi:10.1016/j.jfca.2015.11.004.

26. Cho, K.; Kim, Y.; Wi, S.J.; Seo, J.B.; Kwon, J.; Chung, J.H.; Park, K.Y.; Nam, M.H. Nontargeted metabolite profiling in compatible pathogen-inoculated tobacco (Nicotiana tabacum L. cv. Wisconsin 38) using UPLC-Q-TOF/MS. *J. Agric. Food Chem.* **2012**, *60*, 11015–11028, doi:10.1021/jf303702j.

27. Li, M.; Si, D.; Fu, Z.; Sang, M.; Zhang, Z.; Liu, E.; Yang, W.; Gao, X.; Han, L. Enhanced identification of the in vivo metabolites of Ecliptae Herba in rat plasma by integrating untargeted data-dependent MS 2 and predictive multiple reaction monitoring-information dependent acquisition-enhanced product ion scan. *J. Chromatogr. B Anal. Technol. Biomed. Life Sci.* **2019**, *1109*, 99–111, doi:10.1016/j.jchromb.2019.02.001.

28. Rozema, E.; Popescu, R.; Sonderegger, H.; Huck, C.W.; Winkler, J.; Krupitza, G.; Urban, E.; Kopp, B. Characterization of glucocerebrosides and the active metabolite 4,8-sphingadienine from Arisaema amurense and Pinellia ternata by NMR and CD spectroscopy and ESI-MS/CID-MS. *J. Agric. Food Chem.* **2012**, *60*, 7204–7210, doi:10.1021/jf302085u.

29. Islam, S.; Alam, M.B.; Ahmed, A.; Lee, S.; Lee, S.H.; Kim, S. Identification of secondary metabolites in Averrhoa carambola L. bark by high-resolution mass spectrometry and evaluation for α-glucosidase, tyrosinase, elastase, and antioxidant potential. *Food Chem.* **2020**, *332*, 127377,

doi:10.1016/j.foodchem.2020.127377.

30. Valsecchi, M.; Mauri, L.; Casellato, R.; Ciampa, M.G.; Rizza, L.; Bonina, A.; Bonina, F.; Sonnino, S. Ceramides as possible nutraceutical compounds: Characterization of the ceramides of the moro blood orange (citrus sinensis). *J. Agric. Food Chem.* **2012**, *60*, 10103–10110, doi:10.1021/jf3027414.

**Supplementary table S5:** $^{13}C$ and $^{1}H$ NMR-shifts of compounds **26, 27, 29** and of apigenin (pyridine-$d_5$, 700 and 175 MHz, respectively)

| position | 26 δC (ppm) | δH (ppm) | 27 δC (ppm) | δH (ppm) | 29 δC (ppm) | δH (ppm) | apigenin δC (ppm) | δH (ppm) |
|---|---|---|---|---|---|---|---|---|
| 2 | 164.5 | - | 164.3 | - | 164.3 | - | 164.6 | - |
| 3 | 103.6 | 6.89 s | 103.4 | 6.96 s | 103.4 | 6.95 s | 104.0 | 6.93 s |
| 4 | 183.0 | - | 183.1 | - | 183.0 | - | 182.8 | - |
| 4a | 105.0 | - | 104.9 | - | 104.8 | - | 105.0 | - |
| 5 | 162.8 | - | 162.7 | - | 162.6 | - | 163.2 | - |
| 6 | 100.7 | 7.04 s | 100.7 | 7.00 s | 100.9 | 7.01 s | 100.1 | 6.76 br s |
| 7 | 166.6 | - | 166.7 | - | n. d. | - | 165.9 | - |
| 8 | 101.2 | - | 101.0 | - | 101.3 | - | 94.9 | 6.84 br s |
| 8a | n. d. | - | n.d | - | n. d. | - | 158.5 | - |
| 1´ | 122.5 | - | 122.6 | - | 122.5 | - | 122.3 | - |
| 2´ | 128.7 | 7.84 d (8.5 Hz) | 109.4 | 7.40 br s | 109.4 | 7.37 br s | 129.0 | 7.94 d (9.0 Hz) |
| 3´ | 116.9 | 7.09 d (8.5 Hz) | 148.8 | - | 148.8 | - | 116.9 | 7.23 d (9.0 Hz) |
| 4´ | 162.5 | - | 152.2 | - | 152.2 | - | 162.7 | - |
| 5´ | 116.9 | 7.09 d (8.5 Hz) | 116.8 | 7.13 d (8.3 Hz) | 116.9 | 7.12 d (8.5 Hz) | 116.9 | 7.23 d (9.0 Hz) |
| 6´ | 128.7 | 7.84 d (8.5 Hz) | 120.9 | 7.50 d (8.3 Hz) | 121.0 | 7.50 d (8.5 Hz) | 129.0 | 7.94 d (9.0 Hz) |
| 2´´ | 164.5 | - | 164.6 | - | 164.3 | - | - | - |
| 3´´ | 103.6 | 6.89 s | 103.4 | 6.90 s | 103.4 | 6.95 s | - | - |
| 4´´ | 183.0 | - | 183.1 | - | 183.0 | - | - | - |
| 4a´´ | 105.0 | - | 104.9 | - | 104.8 | - | - | - |
| 5´´ | 162.8 | - | 162.7 | - | 162.6 | - | - | - |
| 6´´ | 100.7 | 7.04 s | 100.7 | 7.01 s | 100.9 | 7.01 s | - | - |
| 7´´ | 166.6 | - | n. d. | - | n. d. | - | - | - |
| 8´´ | 101.2 | - | 101.0 | - | 101.3 | - | - | - |
| 8a´´ | n. d. | - | n. d. | - | n. d. | - | - | - |
| 1´´´ | 122.5 | - | 122.6 | - | 122.5 | - | - | - |
| 2´´´ | 128.7 | 7.84 d (8.5 Hz) | 128.6 | 7.83 d (8.6 Hz) | 109.4 | 7.37 br s | - | - |
| 3´´´ | 116.9 | 7.09 d (8.5 Hz) | 116.8 | 7.10 d (8.6 Hz) | 148.8 | - | - | - |
| 4´´´ | 162.5 | - | 162.6 | - | 152.2 | - | - | - |
| 5´´´ | 116.9 | 7.09 d (8.5 Hz) | 116.8 | 7.10 d (8.6 Hz) | 116.9 | 7.12 d (8.5 Hz) | - | - |
| 6´´´ | 128.7 | 7.84 d (8.5 Hz) | 128.6 | 7.83 d (8.6 Hz) | 121.0 | 7.50 d (8.5 Hz) | - | - |
| 3´$CH_3$ | - | - | 55.7 | 3.81 s | 56.0 | 3.81 s | - | - |
| 3´´´-$CH_3$ | - | - | - | - | 56.0 | 3.81 s | - | - |

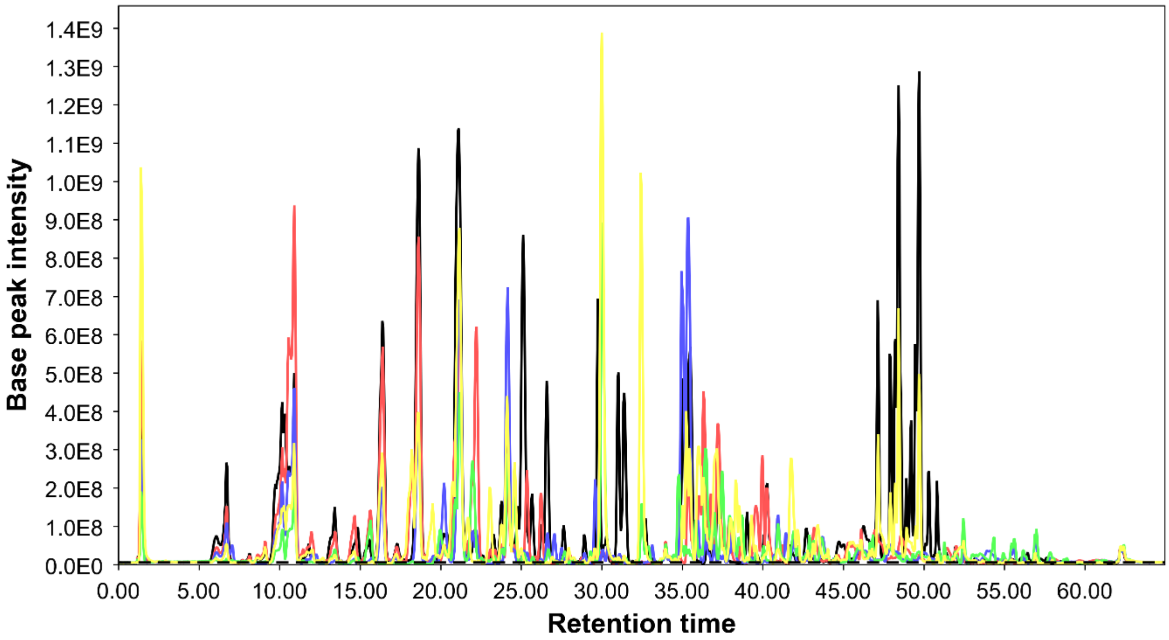


**Figure S1.** Superposition of HESI negative ion mode base peak chromatograms (*m/z* 133-2000) of five representative ethanolic leaf extracts from different Lonicera species. black: L3_1 (*L. hypoglauca*); red: L10_1 (*L. confusa*); blue: L32_1 (L. *acuminata*); green: L24_1 (*L. japonica*); yellow: L15_1 (*L. macrantha*). Superposition of LC-HRMS raw data was generated using MzMine 2 (doi:10.1186/1471-2105-11-395.)

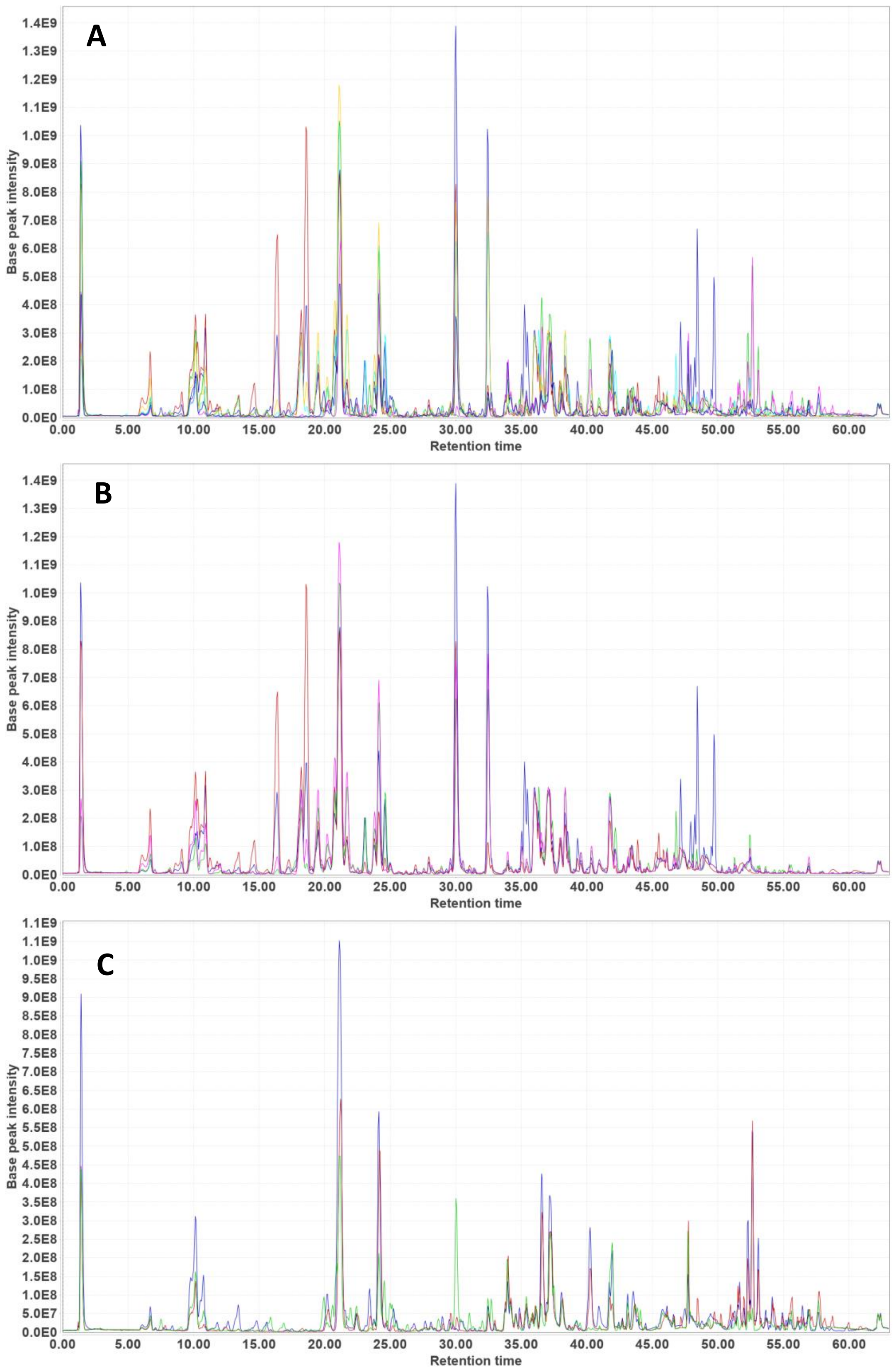


**Figure S2:** Superposition of UHPLC-HRMS HESI negative ion mode base peak chromatograms (*m/z* 132-2000) of **(A)** all *Lonicera macrantha* samples (L12, 15, 16, 17, 21, 31, 33), **(B)** *L. macrantha* samples of cluster 1 ([t1]/[t2] = -0.5/-0.5; samples L12, 15, 16, 17), **(C)** *L. macrantha* samples of cluster 2 (t1]/[t2] = -0.5/+0.5; samples 31, 31, 33) in PCA score scatter plot [t1]/[t2] (Figure 1). Generated with MzMine 2 (doi:10.1186/1471-2105-11-395.)

.

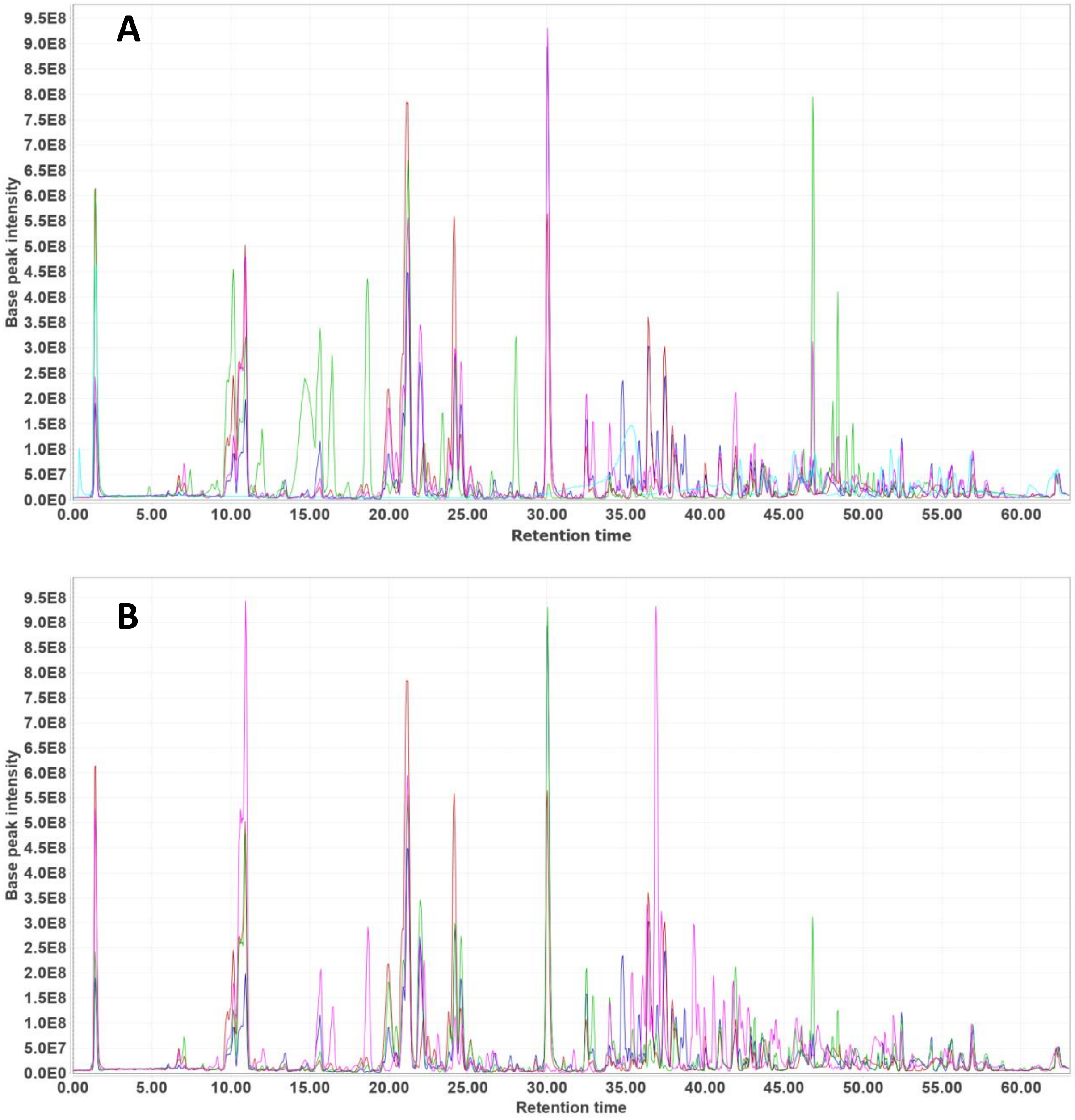


**Figure S3**: Superposition of UHPLC-HRMS HESI negative ion mode base peak chromatograms (*m/z* 132-2000) of **(A)** all *Lonicera japonica* samples (L23, 24, 25, 35, 36) and **(B)** *L. japonica* leaf samples only (L23, 24, 25, 36). Generated with MzMine 2 (doi:10.1186/1471-2105-11-395.)

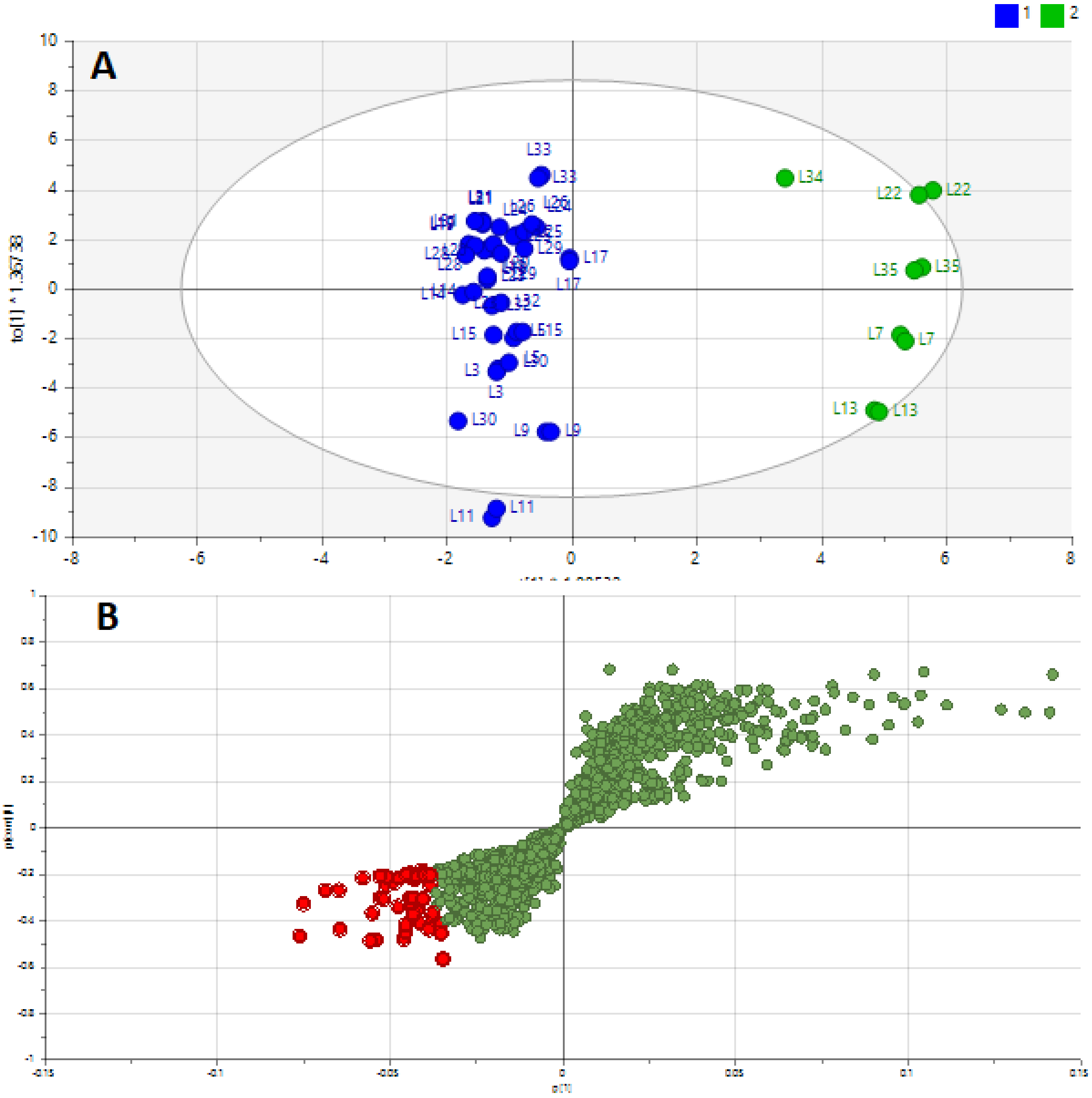


**Figure S4:** OPLS-DA model of CD-processed UHPLC-HRMS data in correlation with activity on transactivation of an NF-κB-driven luciferase reporter gene in TNF-α- stimulated HEK293/NF-κB-luc cells. **A:** score scatter plot; blue: highly active samples; green: samples with low activity; moderately active samples were excluded from the model. **B:** S-Plot of the OPLS-DA model. Variables most likely correlated with bioactivity are marked in red and listed in Table 2 and Supplementary Table S4.

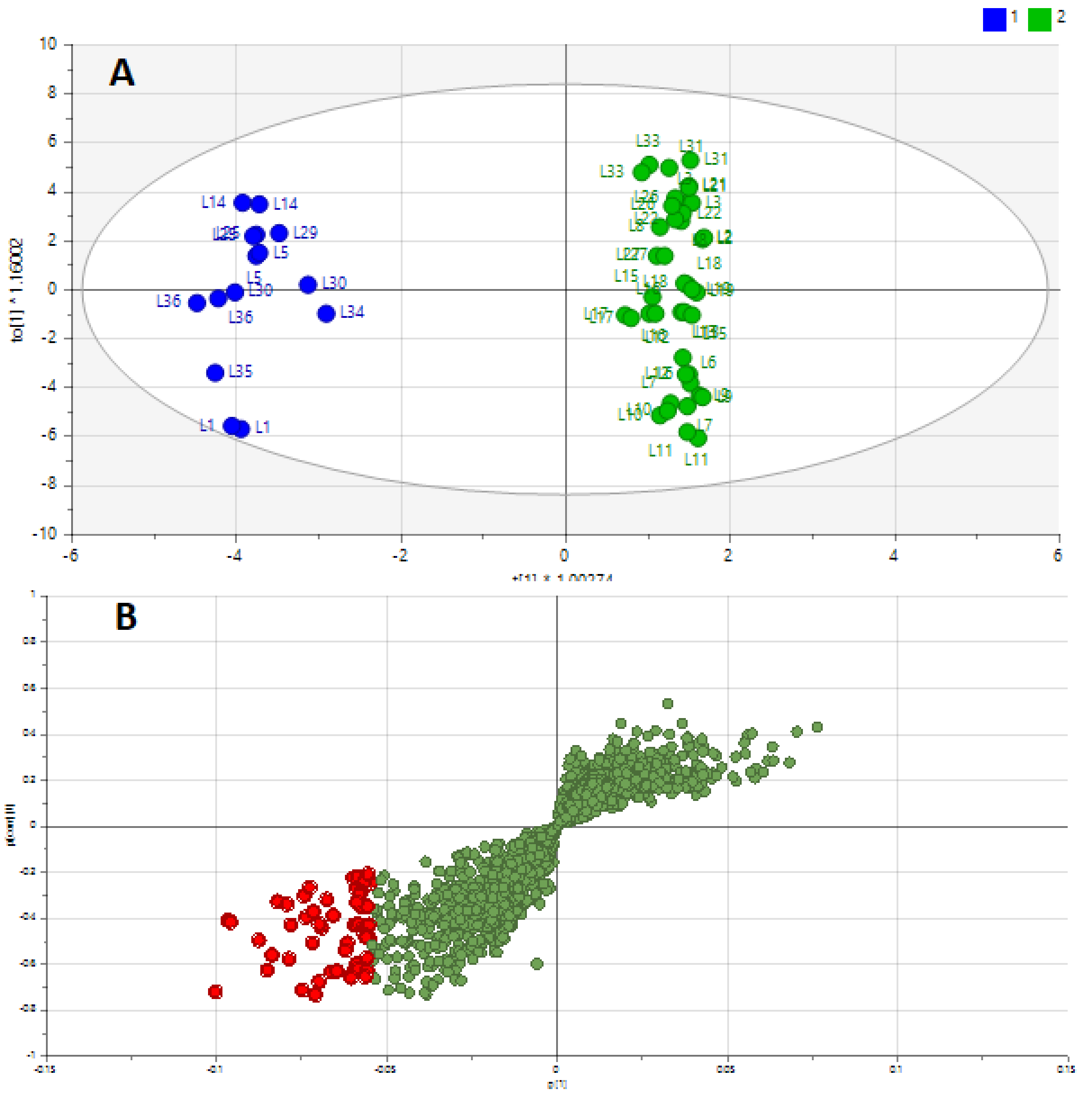


**Figure S5:** OPLS-DA model of CD-processed UHPLC-HRMS data in correlation with activity on IL-8 expression in LPS-stimulated HUVECtert cells. **A:** score scatter plot; blue: active samples; green: inactive samples; moderately active samples were excluded from the model. **B:** S-Plot of the OPLS-DA model. Variables most likely correlated with bioactivity are marked in red and listed in Table 2 and Supplementary Table S4.

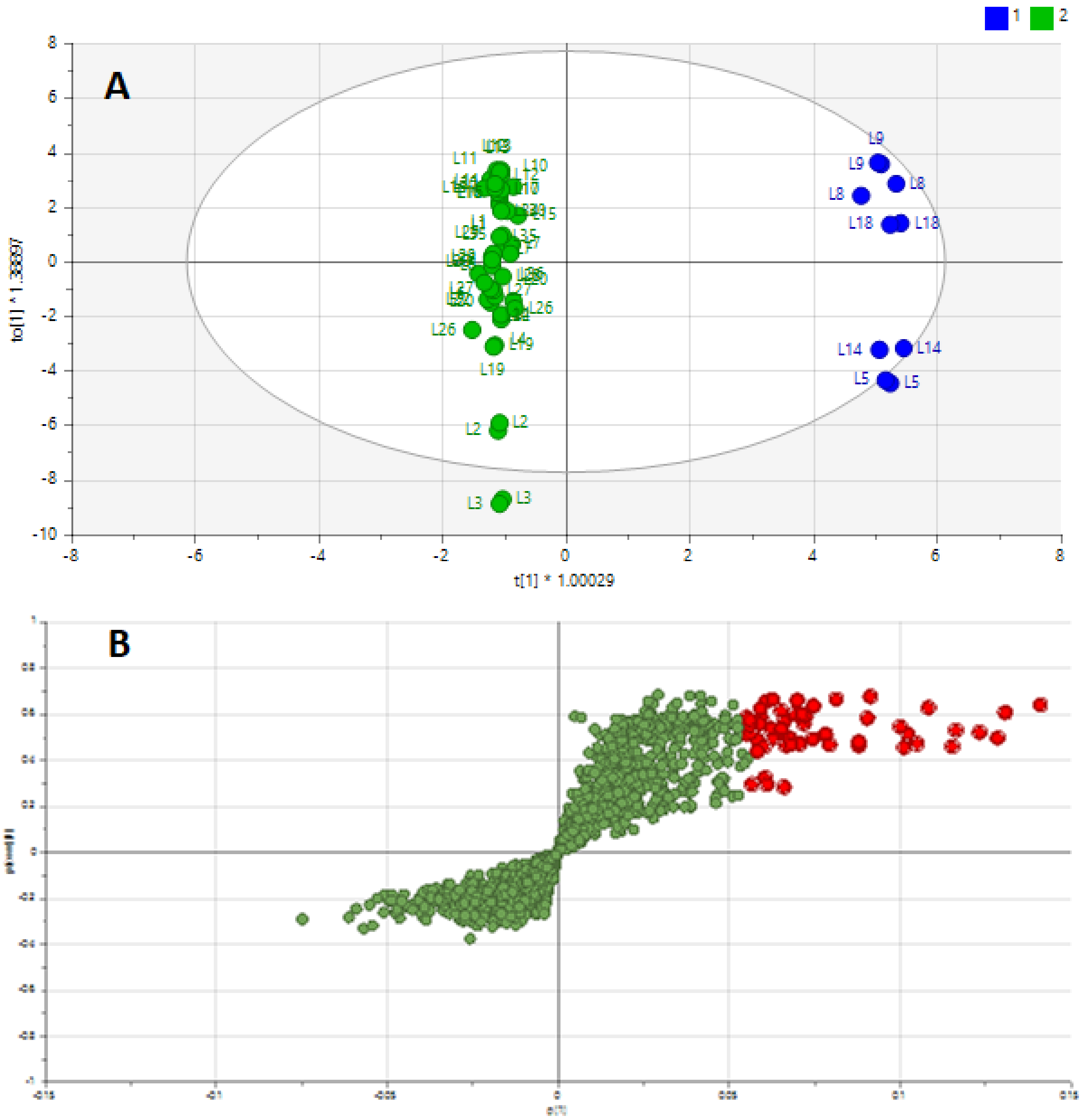


**Figure S6:** OPLS-DA model of CD-processed UHPLC-HRMS data in correlation with activity on NO production in LPS/IFNγ- stimulated RAW 264.7 mouse macrophages. **A:** score scatter plot; blue: active samples; green: inactive samples; moderately active samples were excluded from the model**. B**: S-Plot of the OPLS-DA model. Variables most likely correlated with bioactivity are marked in red and listed in Table 2 and Supplementary Table S4.

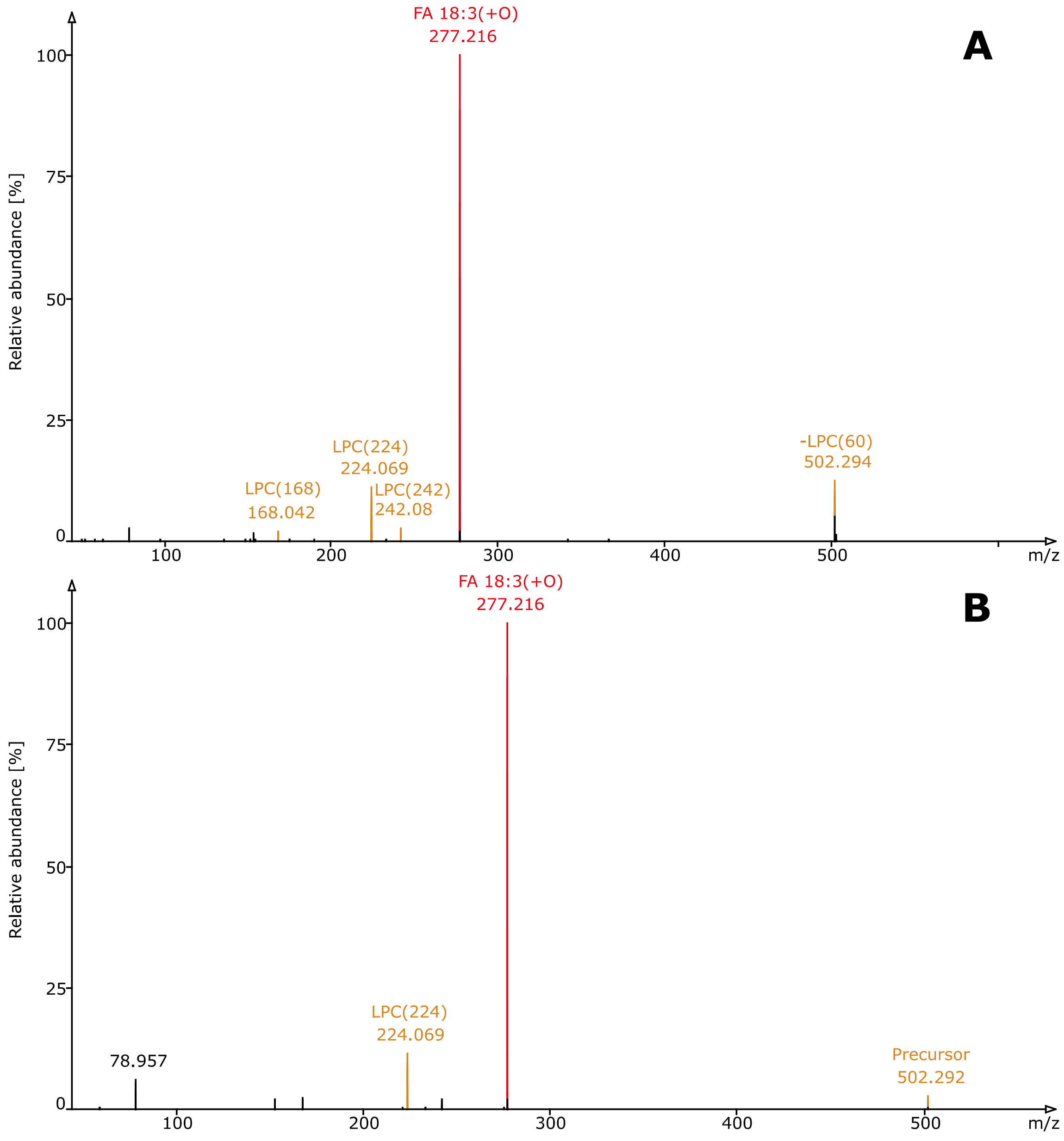


**Supplementary Figure S7:** MS/MS spectra of LPC 18:3 (**40**), assigned with LDA. **A**, precursor: *m/z* 562.314 [M+HCOO]$^-$, retention time: 44.18 min, file: Lonicera-L25-fl-1_1; **B**, precursor *m/z* 502.290 [M-$CH_3$]$^-$, retention time: 44.08 min; file: Lonicera-L25-fl-1_1

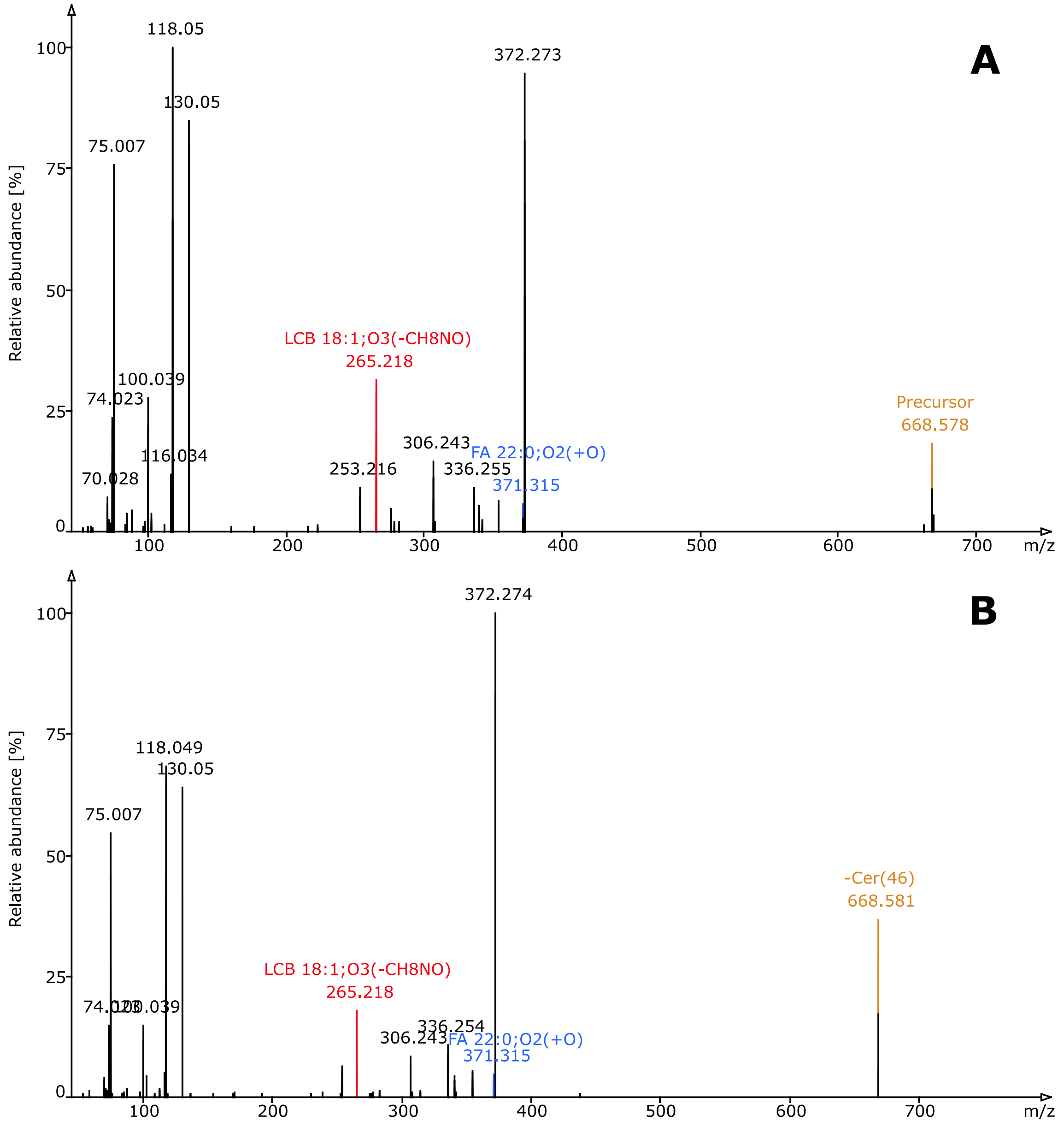


**Supplementary Figure S8:** MS/MS spectra of Cer 18:1;O3/22:0;O2 (**59**), assigned with LDA. **A**, precursor: *m/z* 668.580 [M-H]$^{-}$, retention time: 54.32 min, file: Lonicera-jap-fl-1_1; **B**, precursor *m/z* 714.590 [M+HCOO]$^{-}$, retention time: 54.27 min, file: Lonicera-jap-fl-1_1

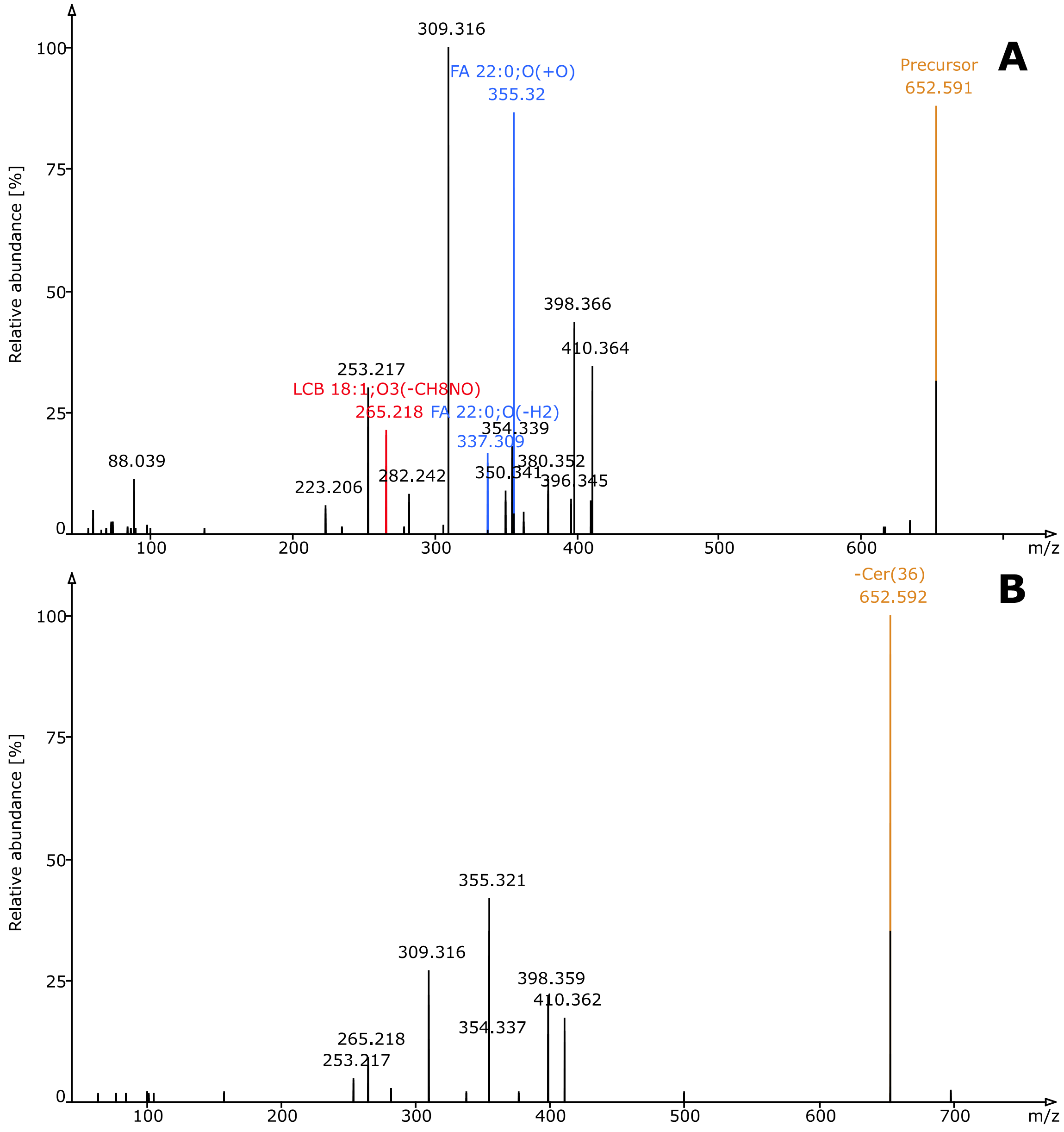


**Supplementary figure S9**: MS/MS spectra of Cer 18:1;O3/22:0;O (**60**), assigned with LDA. **A**, precursor: *m/z* 652.590 $[M-H]^-$, retention time: 55.50 min, file: Lonicera-jap-fl-1_1; **B**, precursor *m/z* 688.560 $[M+Cl]^-$, retention time: 55.49 min; file: Lonicera-jap-fl-1_1

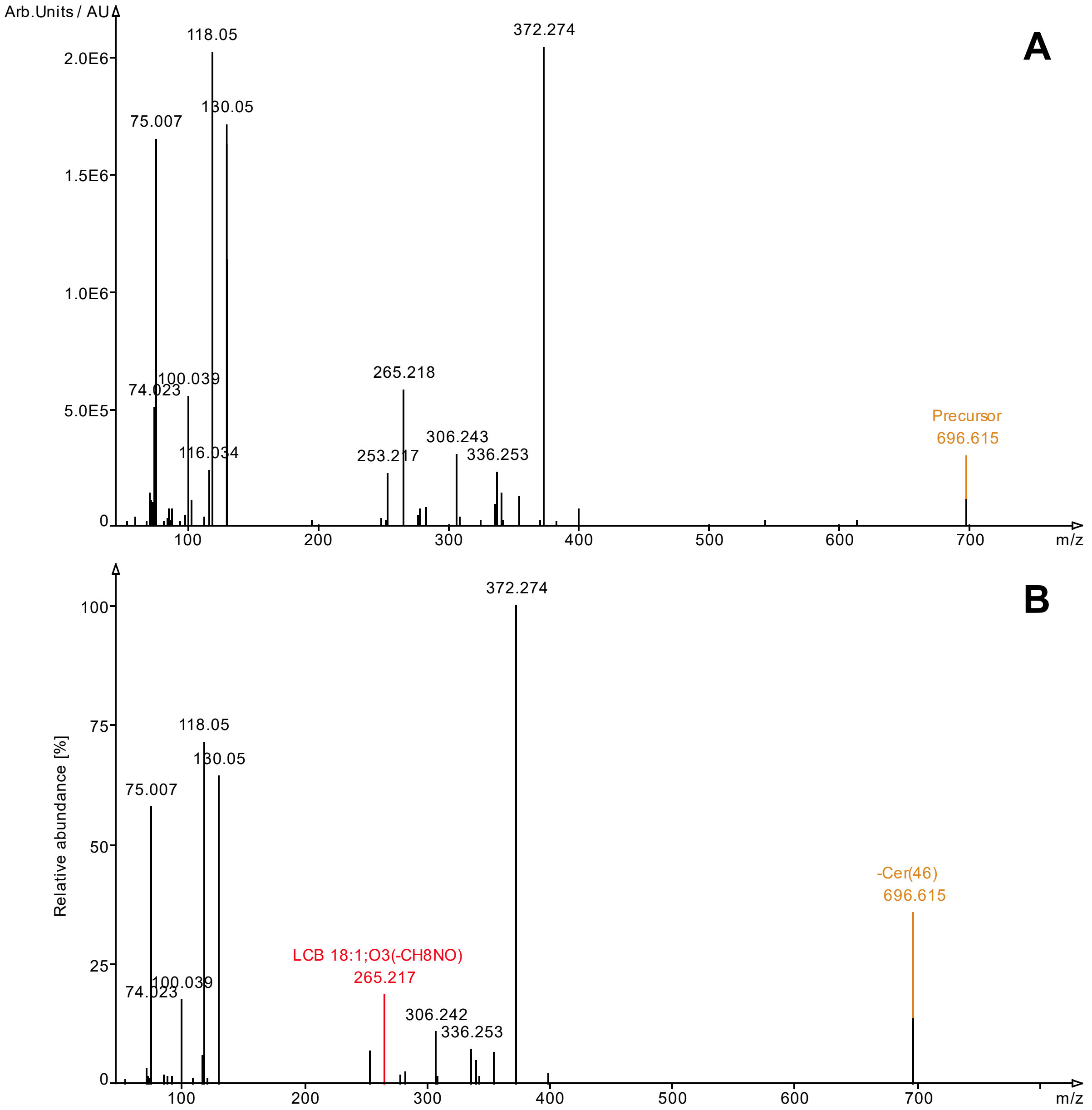


**Supplementary figure S10:** MS/MS spectra of Cer 18:1;O3/24:0;O2 (**61**), assigned with LDA. **A**, precursor: *m/z* 696.610 [M-H]$^-$, retention time: 55.55 min, file: Lonicera-jap-fl-1_1; **B**, precursor *m/z* 742.619 [M+HCOO]$^-$, retention time: 55.64 min, file: Lonicera-jap-fl-1_1

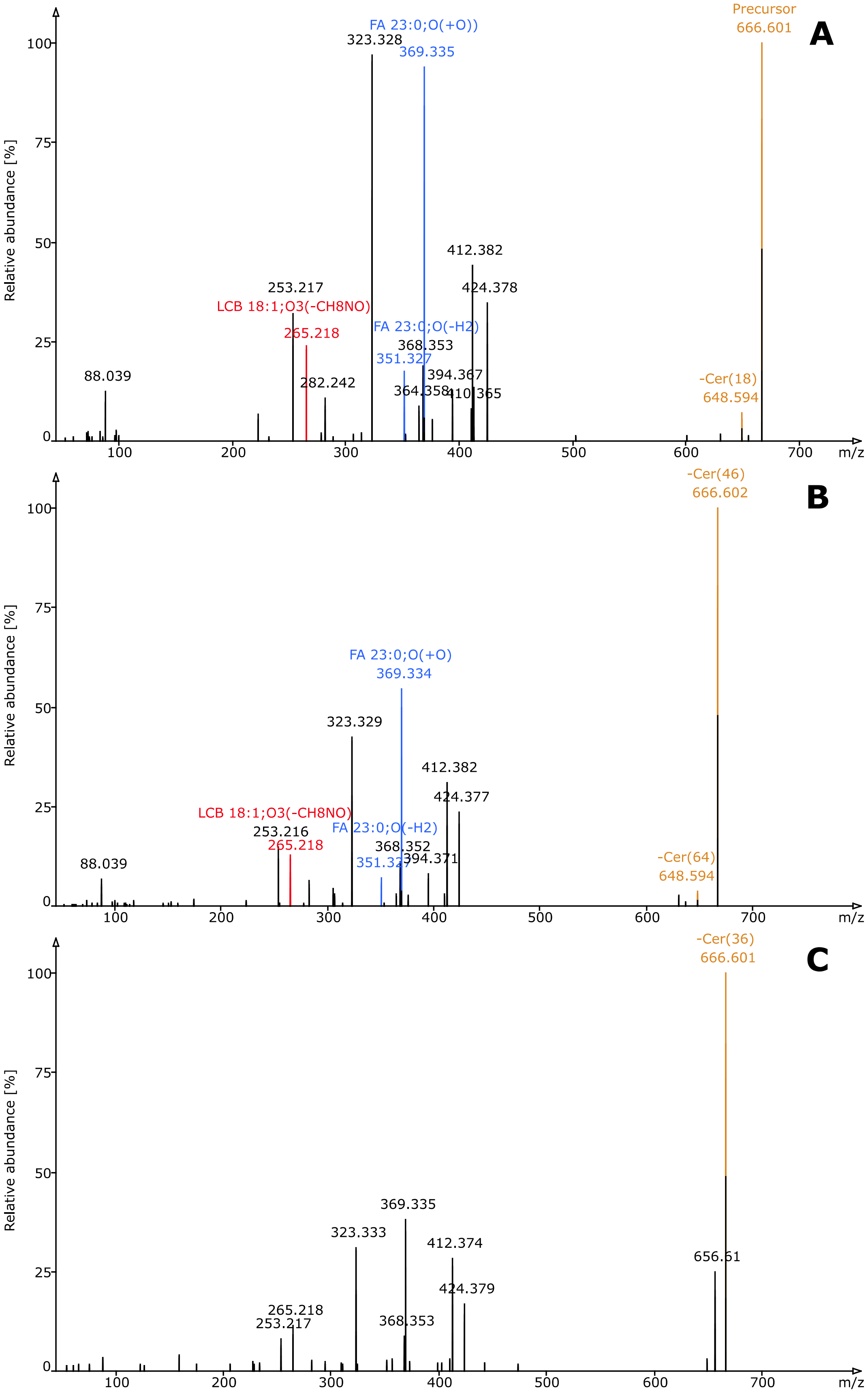


**Supplementary Figure S11:** MS/MS spectra of Cer 18:1;O3/23:0;O; (**62**), assigned with LDA. **A**, precursor: *m/z* 666.600 [M-H]-, retention time: 56.20 min, file: Lonicera-jap-fl-1_1; **B**, precursor *m/z* 712.610 [M+HCOO]- , retention time: 56.18 min, file: Lonicera-jap-fl-1_1; **C**: precursor: 702.580 [M+Cl]-, retention time: 56.19 min, file: Lonicera-jap-fl-1_1

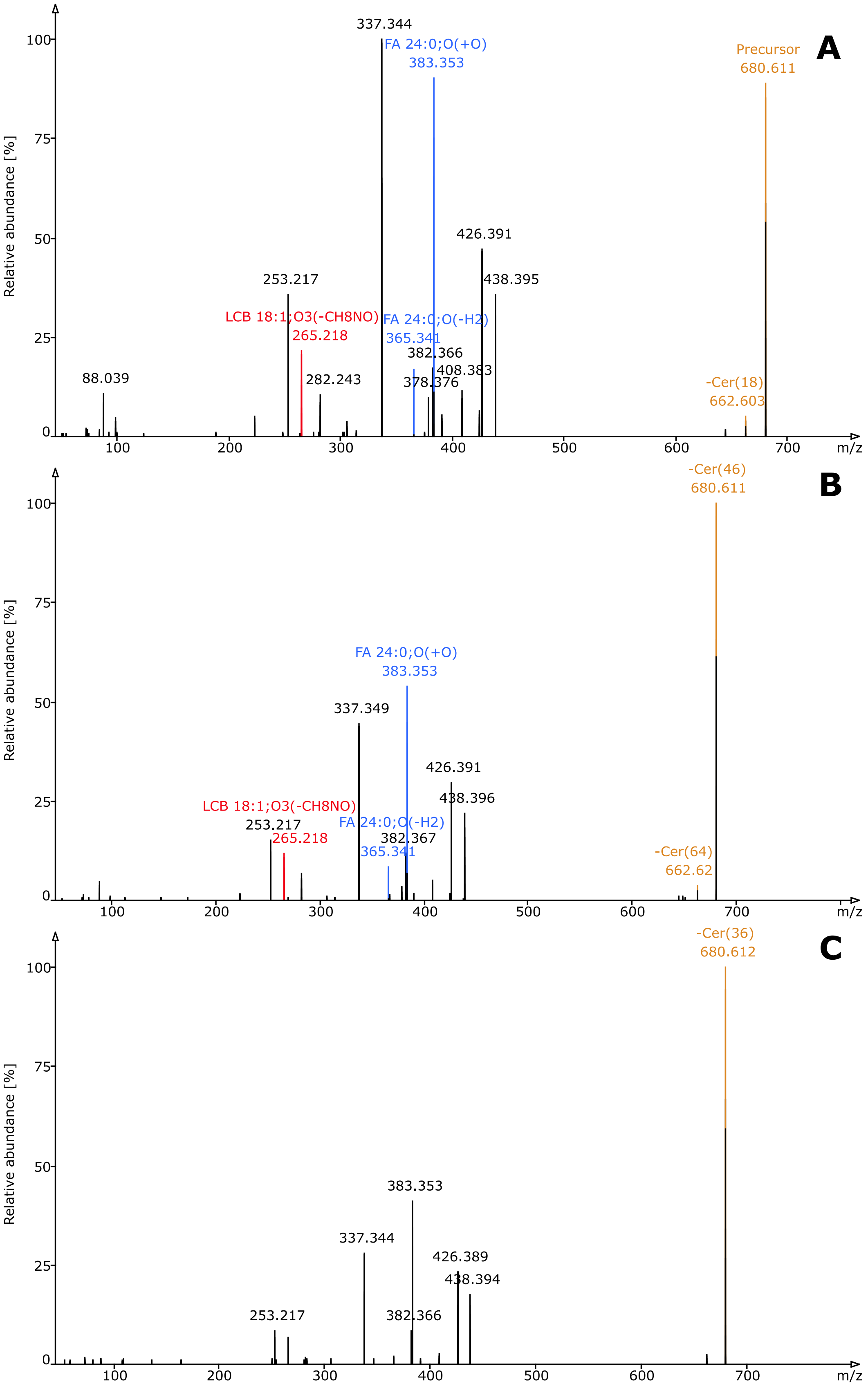


**Supplementary Figure S12**: MS/MS spectra of Cer 18:1;O3/24:0;O; (**63**), assigned with LDA. **A**, precursor: *m/z* 680.620 [M-H]$^-$, retention time: 56.98 min; file: Lonicera-jap-fl-1_1; **B**, precursor *m/z* 726.624 [M+HCOO]$^-$, retention time: 56.98 min, file: Lonicera-jap-fl-1_1; **C**: precursor: 716.598 [M+Cl]$^-$, retention time: 56.92 min; file: Lonicera-jap-fl-1_1

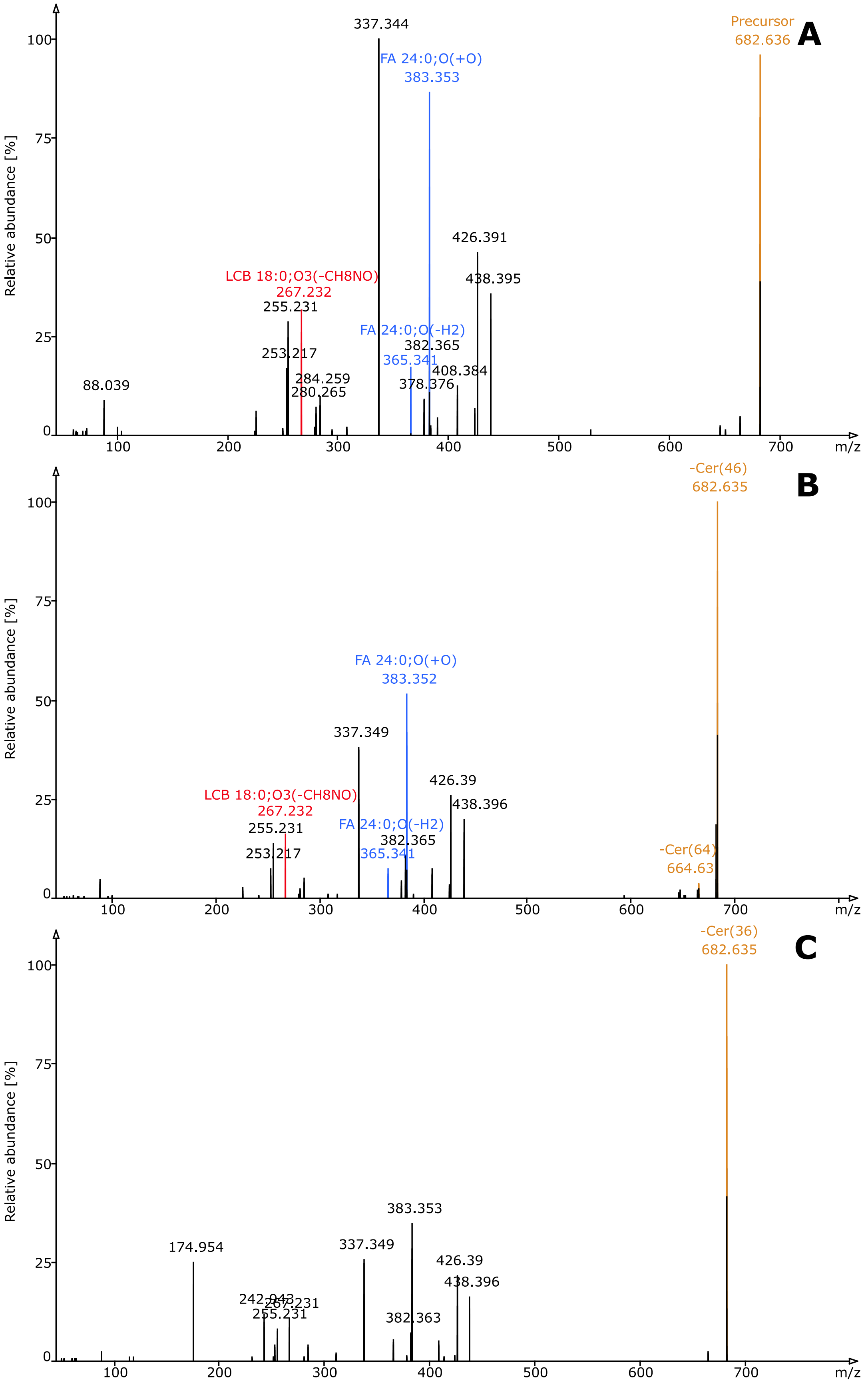


**Supplementary Figure S13:** MS/MS spectra of Cer 18:0;O3/24:0;O (**64**), assigned with LDA. **A**, precursor: *m/z* 682.630 [M-H]$^-$, retention time: 57.95 min, file: Lonicera-jap-fl-1_1; **B**, precursor *m/z*

728.640 [M+HCOO]⁻, retention time: 57.88 min, file: Lonicera-jap-fl-1_1; **C**: precursor: *m/z* 718.610 [M+Cl]⁻, retention time: 57.73 min; file: Lonicera-jap-fl-1_1

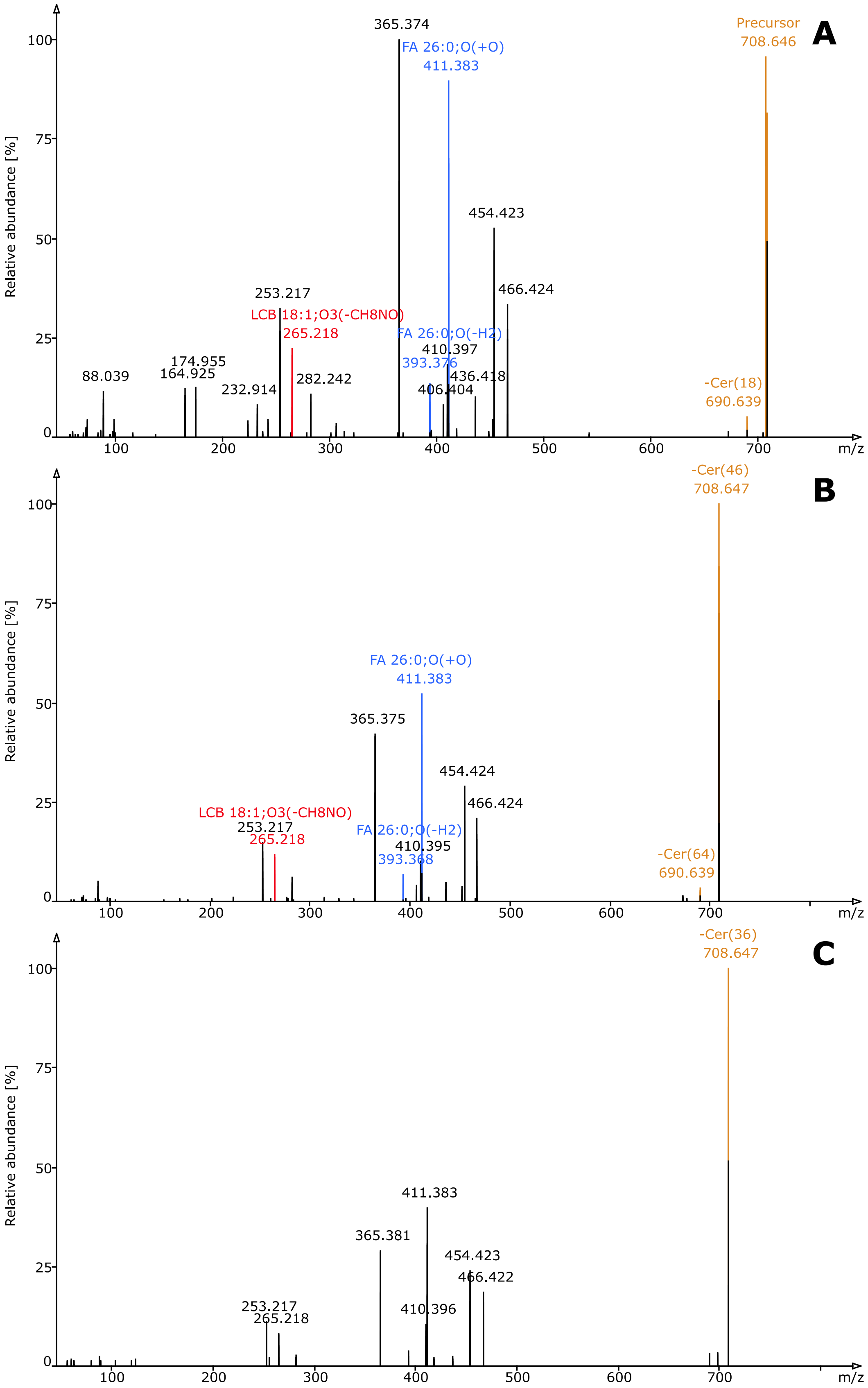


**Supplementary Figure S14:** MS/MS spectra of Cer 18:1;O3/26:0;O (**65**), assigned with LDA. **A**, precursor: *m/z* 708.650 [M-H]⁻, retention time: 58.88; file: Lonicera-jap-fl-1_1; **B**, precursor *m/z* 754.650

[M+HCOO]-, retention time: 58.87 min; file: Lonicera-jap-fl-1_1; **C**: precursor: *m/z* 744.627 [M+Cl]-, retention time: 58.86 min; file: Lonicera-jap-fl-1_1

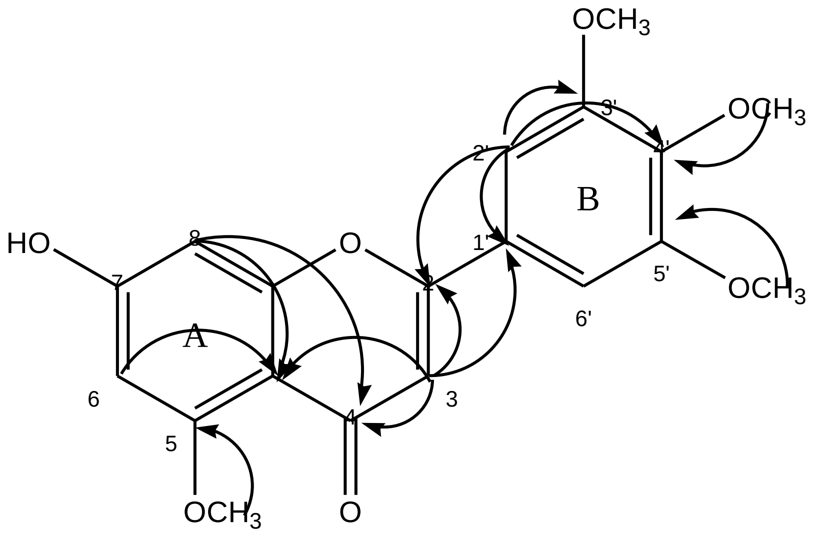


**22**

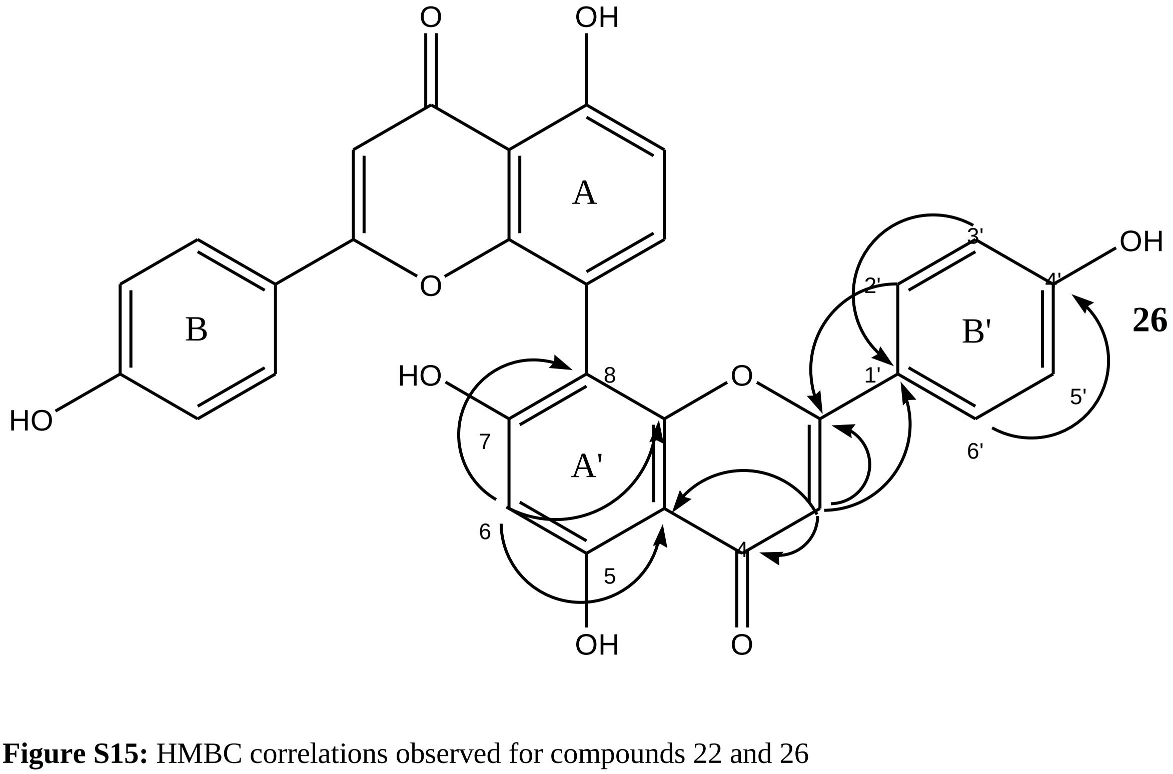


**26**

**Figure S15:** HMBC correlations observed for compounds 22 and 26

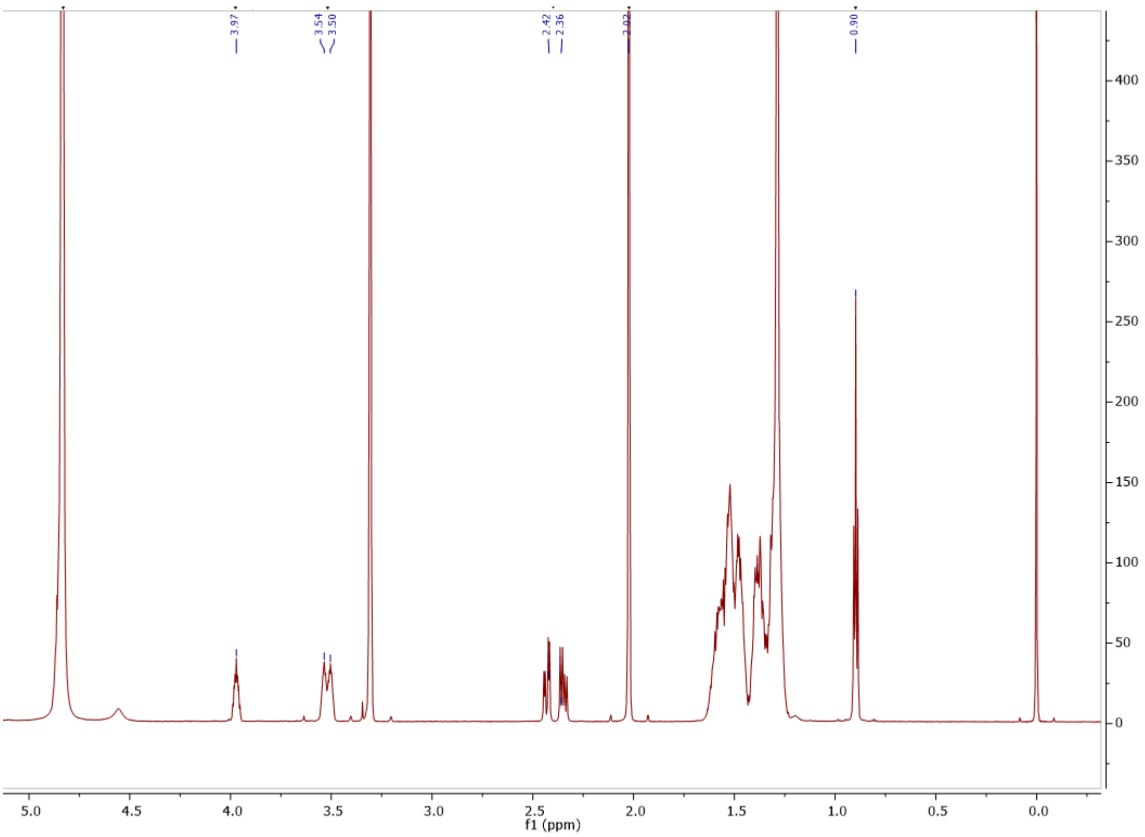


**Supplementary figure S16:** $^{1}$H NMR spectrum of compound **46** in MeOH-$d_4$ (700 MHz, TMS, 298 K)

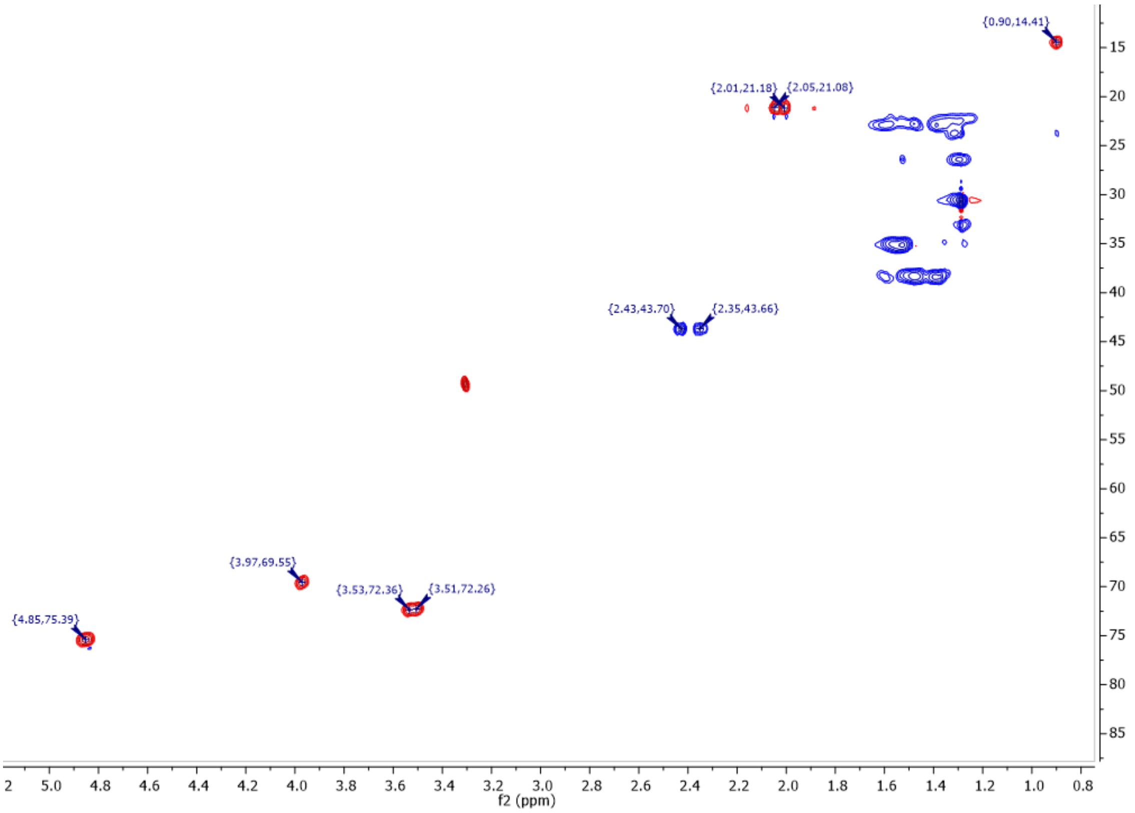


**Supplementary figure S17:** HSQC spectrum of compound **46** in MeOH-$d_4$ (TMS, 298 K)

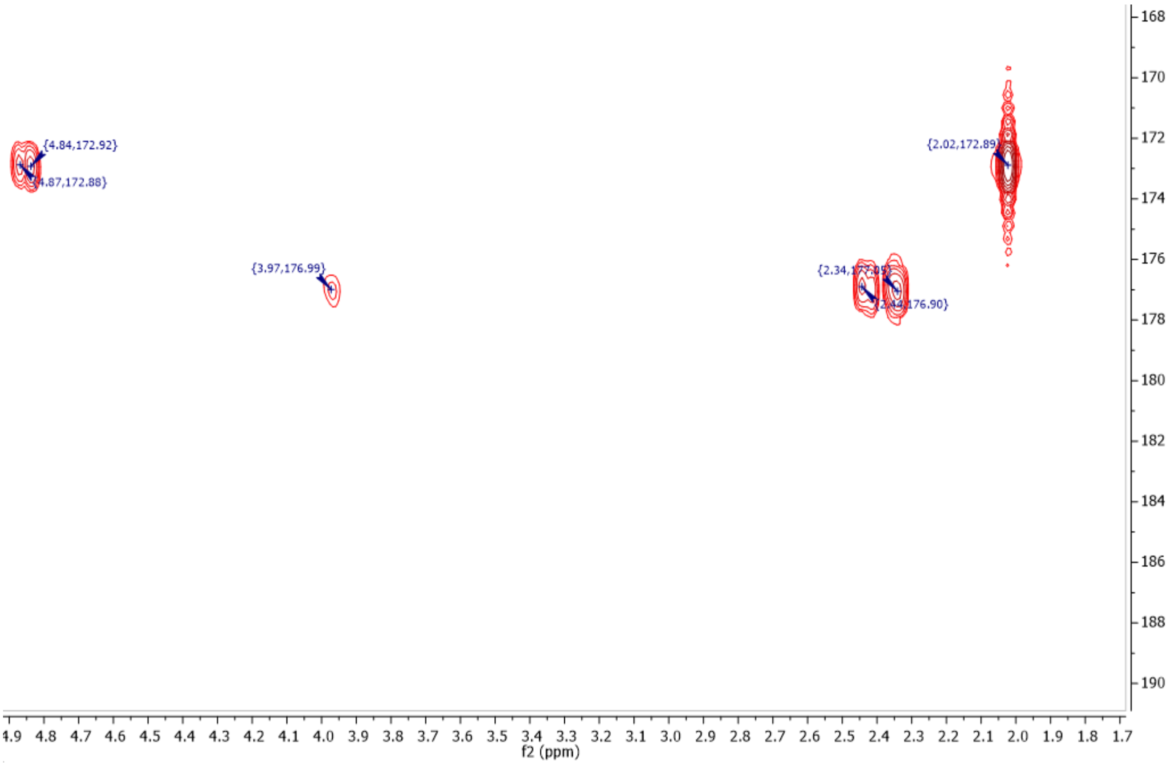


**Supplementary figure S18:** Correlations to carbonyl functions in the HMBC spectrum of compound **46** in MeOH-d$_4$ (TMS, 298 K)

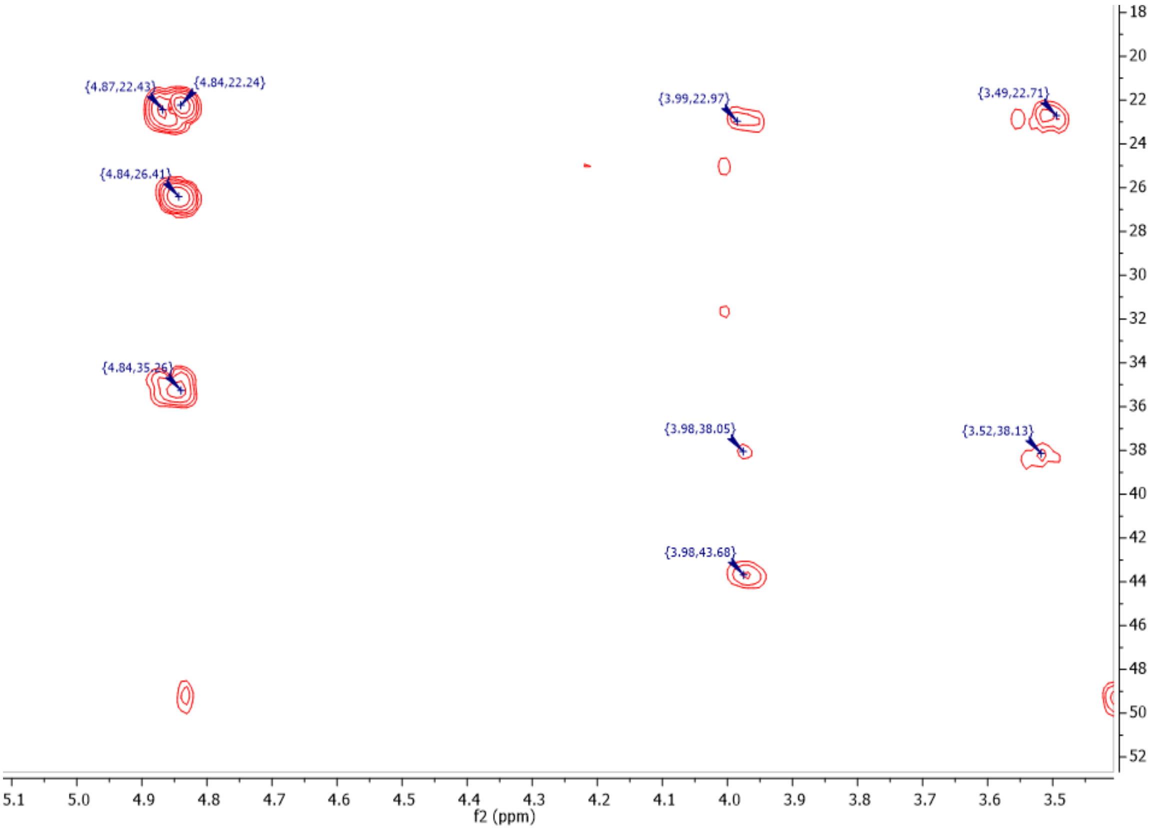


**Supplementary figure S19:** Non-carbonyl HMBC correlations of CHOH and CHOCOCH$_3$ groups in the HMBC spectrum of compound **46** in MeOH-d$_4$ (TMS, 298 K)

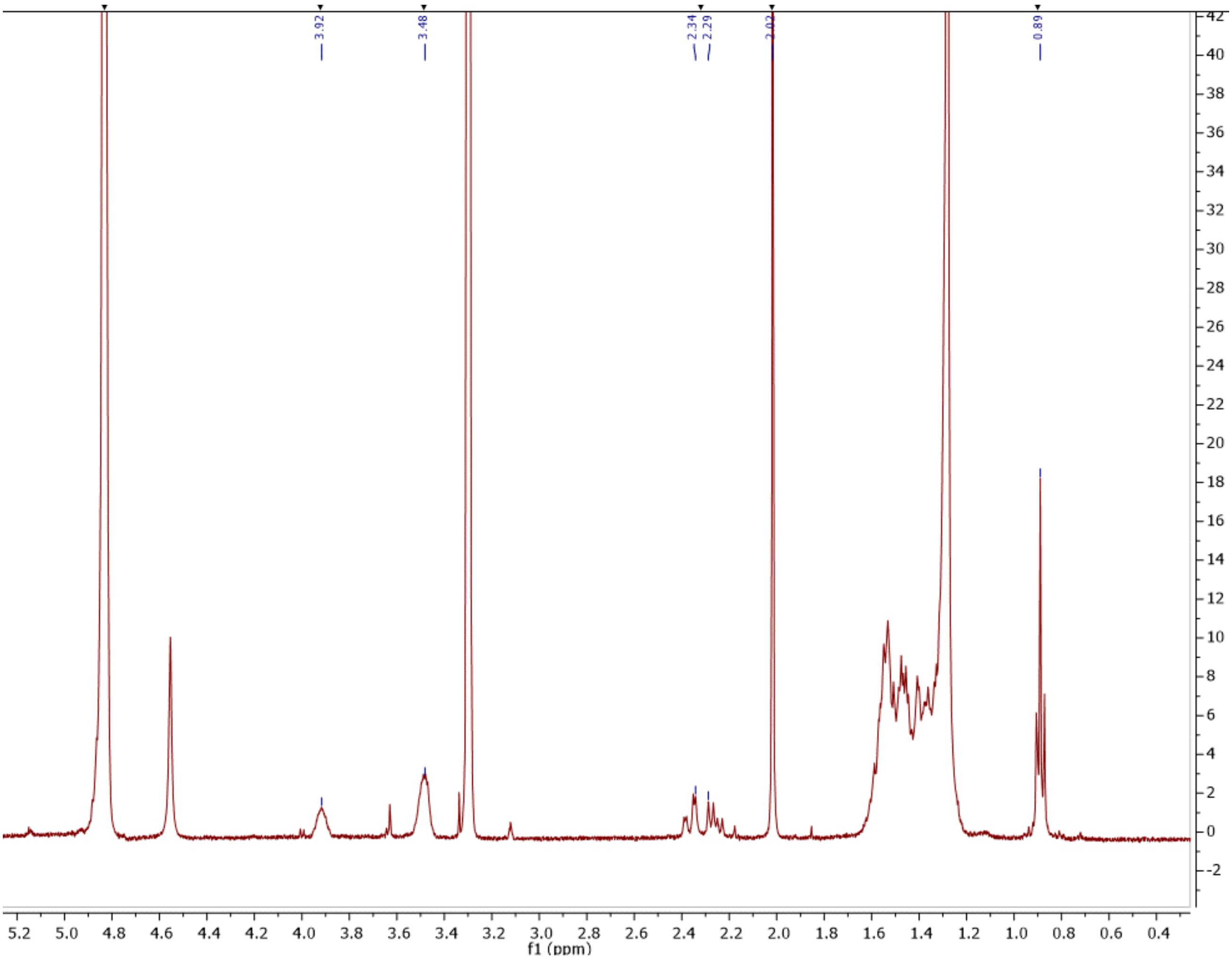


**Supplementary figure S20:** $^{1}$H NMR spectrum of compound **43** in MeOH-$d_4$ (700 MHz, TMS, 298 K)

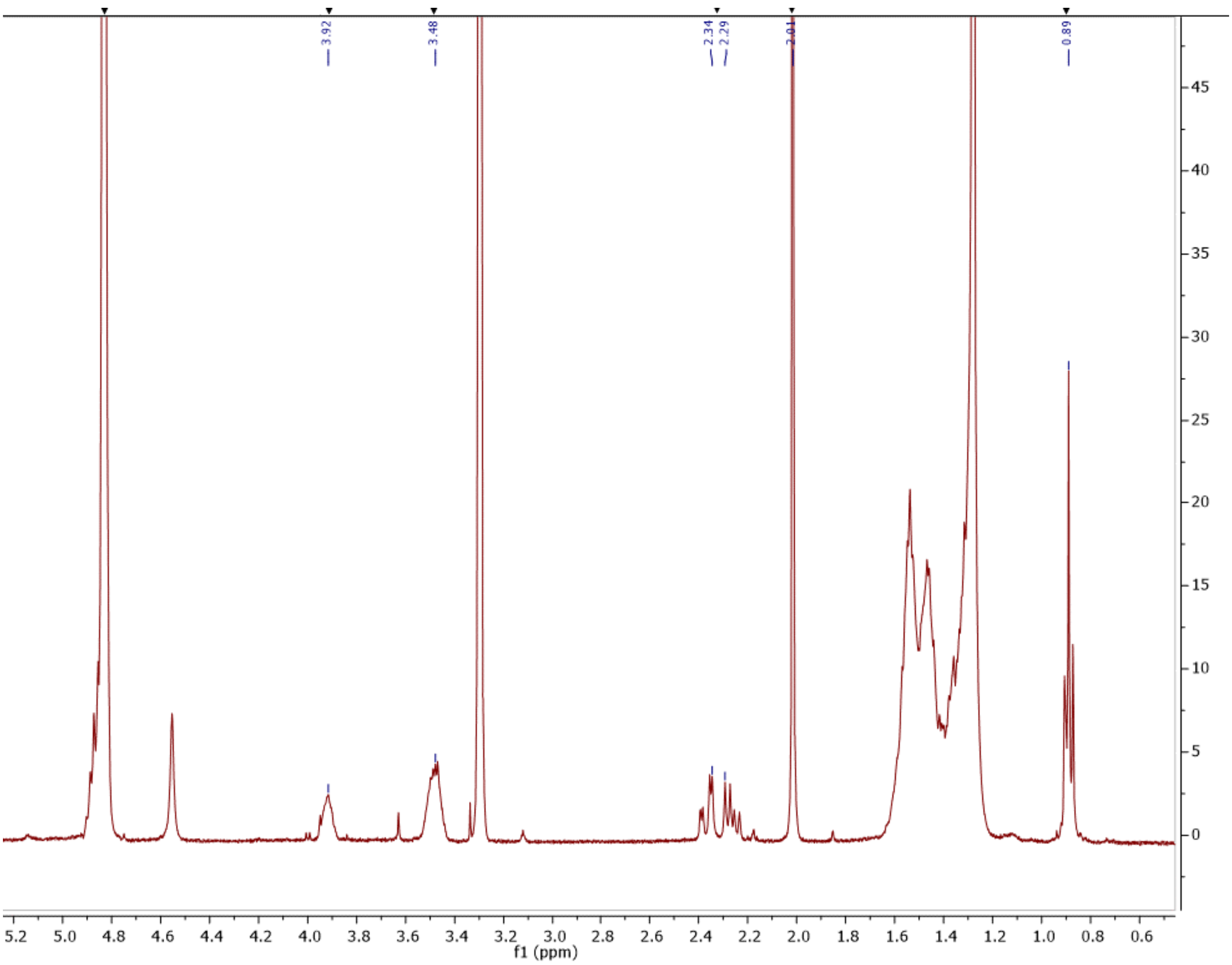


**Supplementary figure S21:** $^{1}H$ NMR spectrum of compound **44** in MeOH-$d_4$ (700 MHz, TMS, 298 K)

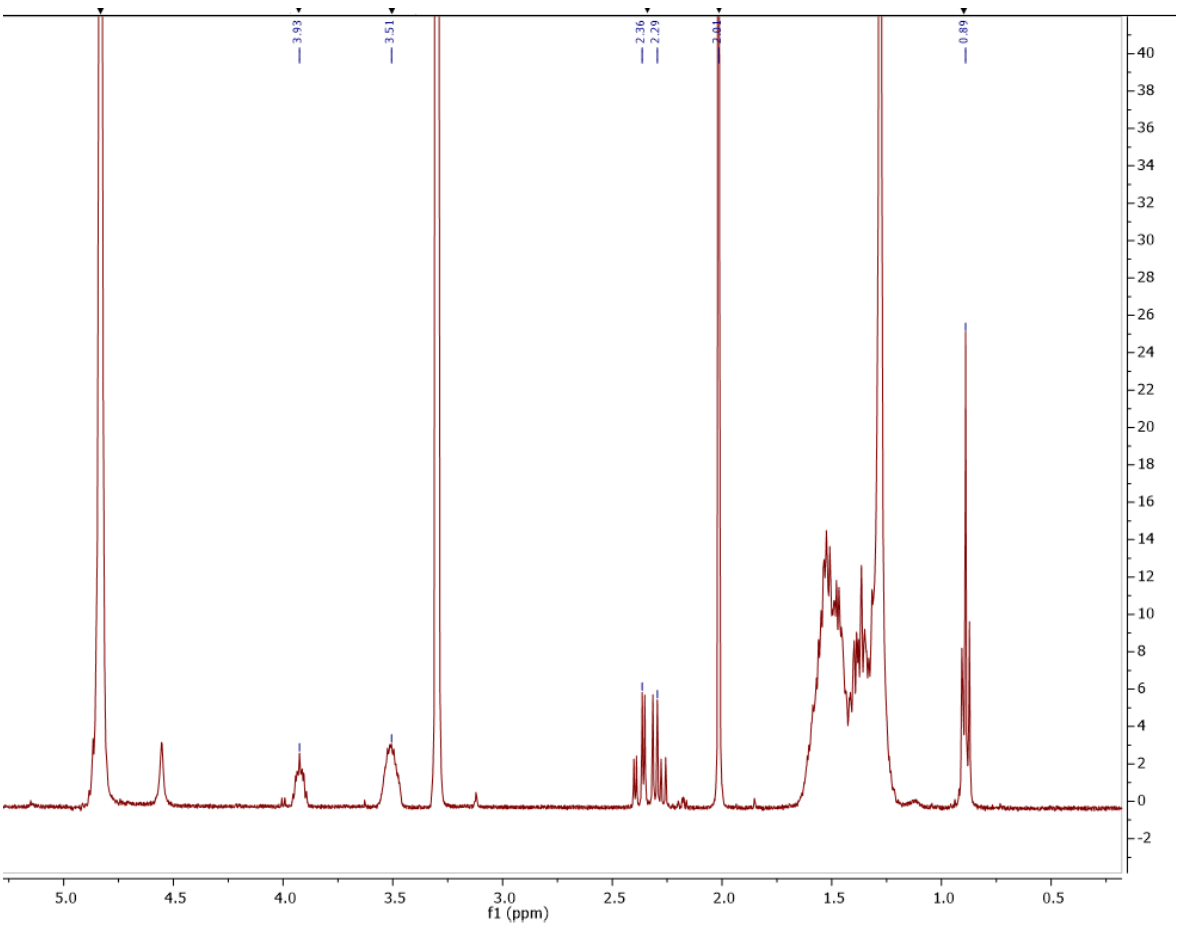


**Supplementary figure S22:** $^{1}H$ NMR spectrum of compound **54** in MeOH-$d_4$ (700 MHz, TMS, 298 K)
